# Synthesis and Anti-Proliferative Activity of 5-Benzoyl and 5-Benzylhydroxy Derivatives of 3-Amino-2-Arylcarboxamido-Thieno[2-3-*b*]Pyridines

**DOI:** 10.3390/ijms241411407

**Published:** 2023-07-13

**Authors:** Bailey Morphet, Shaun W. P. Rees, Natalie A. Haverkate, Hamid Aziz, Euphemia Leung, Lisa I. Pilkington, David Barker

**Affiliations:** 1 School of Chemical Sciences, University of Auckland, Auckland 1010, New Zealandshaun.rees@auckland.ac.nz (S.W.P.R.); nhav422@aucklanduni.ac.nz (N.A.H.); 2 Department of Chemistry, Quaid-I-Azam University, Islamabad 45320, Pakistan; hamidaziz@chem.qau.edu.pk; 3 Department of Chemistry, Rawalpindi Women University, Rawalpindi 46300, Pakistan; 4 Auckland Cancer Society Research Centre, Department of Molecular Medicine and Pathology, University of Auckland, Auckland 1023, New Zealand; e.leung@auckland.ac.nz; 5 Te Pūnaha Matatini, Auckland 1142, New Zealand; 6 The MacDiarmid Institute for Advanced Materials and Nanotechnology, Victoria University of Wellington, Wellington 6012, New Zealand

**Keywords:** PI-PLC, thieno[2-3-*b*]pyridines, anti-proliferative, structure–activity relationship, heterocycles

## Abstract

3-Amino-2-arylcarboxamido-thieno[2-3-*b*]pyridines have been previously described as having potent anti-proliferative activity against MDA-MB-231 and HCT116 cancer cell lines. The mechanism by which these molecules prevent cancer cell growth is proposed to be through interfering with phospholipid metabolism via inhibition of PI-PLC, along with other cellular processes. Previously, 5-cinnamyl derivatives of these thieno[2-3-*b*]pyridines have been shown to have enhanced anti-proliferative activity compared to compounds lacking this moiety, indicating a tethered aromatic ring is important for this western region of the pharmacophore. Herein, we report the synthesis and biological evaluation of a library of 40 novel thieno[2-3-*b*]pyridine analogues containing shorter benzoyl or secondary benzyl alcohol tethers at the 5-position, in addition to various substituents on the two phenyl rings present on the molecule. Compounds bearing alcohol functionality had improved efficacy compared to their benzoyl counterparts, in addition to a 2-methyl-3-halogen substitution on the 2-arylcarboxamide ring being important for maximising anti-proliferative activity. The most potent molecules **7h** and **7i** demonstrated IC_50_ concentrations of 25–50 nM against HCT116 and MDA-MB-231 cells, a similar level of activity as previous thienopyridine compounds bearing cinnamyl moieties, suggesting that these novel derivatives with shorter tethers were able to maintain potent anti-proliferative activity, while allowing for a more concise synthesis.

## 1. Introduction

The 3-amino-2-arylcarboxamido-thieno[2,3-*b*]pyridines are a family of drug-like molecules that are well established in the literature for inducing potent anti-proliferative effects in triple-negative breast cancer (MDA-MB-231) and colorectal cancer (HCT116) cell lines [1,2,3,4]. It is postulated that thieno[2,3-*b*]pyridines induce anti-neoplastic action through disrupting phospholipid metabolism by the inhibition of phosphoinositide phospholipase C (PI-PLC). PI-PLC has been suggested as the primary biological target of thieno[2,3-*b*]pyridine compounds, as treatment with these compounds induces cell morphology changes and membrane blebbing that mimics PI-PLC knockdown cells [4]. It has also been shown that treatment of breast cancer cells (MDA-MB-231) with a previous thieno[2,3-*b*]pyridine lead compound induced growth reduction in addition to causing cells to shift from lipid to glucose metabolism, providing further evidence that these compounds act through inferring with lipid metabolism [2]. Additional studies into thienopyridine-containing compounds have identified a range of other potential cellular targets, including TDP-1 [5], P2Y_12_ receptors [6], Adenosine A_2A_ receptors [7], as well as potentially inhibiting microtubule assembly [8,9]. However, at this time, PI-PLC appears to be the most robust biological target for explaining how this particular family of thieno[2-3-*b*]pyridines induce their potent anti-proliferative activity.

**Figure 1:**
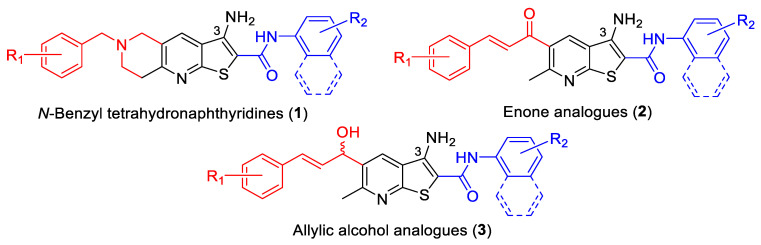
Examples of previous thieno[2-3-*b*]pyridine series investigated for anti-proliferative activity.

Recent SAR investigations into the optimal pharmacophore for this family of molecules have focussed on various related thieno[2,3-*b*]pyridine scaffolds such as the *N*-benzyl tetrahydronaphthyridines (**1**) [1], cinnamyl containing enones (**2**) [1], and allylic alcohols (**3**) [1] (Figure 1). These studies have identified that, for the western fragment of the molecule (red), compounds bearing enone and allylic alcohol linkers significantly outperformed those with tetrahydronaphthyridine rings tethering the thieno[2-3-*b*]pyridine ring to the phenyl ring [1]. It was also found that electron-rich phenyl substitutions (R_1_) such as alkoxy and hydroxy groups improved the activity of these compounds [1]. Molecular docking studies suggested that the phenyl ring of the western fragment sits in a lipophilic alcove of the PI-PLC active site, while the enone and allyl alcohol motifs were able to form hydrogen bonds with the active site via the ketone and hydroxy motif, respectively [1]. In regard to the eastern fragment (blue), it has been identified that the optimal substitution of this 2-arylcarboxamide ring (R_2_) is di-substitution at the 2- and 3-positions with bulky lipophilic groups such as 2-methyl-3-chloro or a naphthalene ring bridged at these positions [1]. It has also been shown that changing the amide linker between the eastern phenyl ring and thieno[2-3-*b*]pyridine core leads to complete loss of anti-proliferative activity [10]. Finally, it has been established that the central thieno[2-3-*b*]pyridine core possesses the optimal arrangement of heteroatoms and that functionalisation of the 3-amino group leads to significant loss of activity, suggesting this group must remain as a primary amine for maximal efficacy [10].

**Figure 2:**
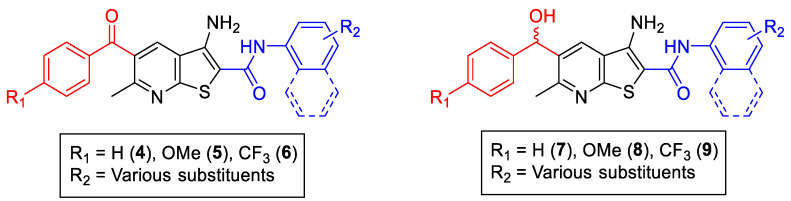
Benzoyl (**4**–**6**) and alcohol (**7**–**9**) thieno[2-3-*b*]pyridine analogues proposed for synthesis and anti-proliferative assessment in this study.

One region of the molecule yet to be optimised is the length of the of the linker separating the pyridine ring from the western phenyl ring. Previous studies have focused on a three-atom linker; however, varying the length of this linker has the potential to improve the binding interactions made by the phenyl ring, in addition to potentially allowing for new interactions by the phenyl substituents that were previously inaccessible. Additionally, these new derivatives would require less steps to synthesise, as they would not require the aldol condensation step used to install the enone motif on previous thieno[2-3-*b*]pyridine series (**2** and **3**). For this reason, a series of benzoyl analogues were proposed, to investigate what effect shortening the alkyl tether between the thienopyridine core and western phenyl ring has on anti-proliferative activity (Figure 2). These analogues would be synthesised with no phenyl substituent (**4**, R_1_ = H) in addition to a collection of compounds with 4-methoxy substitution (**5**, R_1_ = 4-OMe) as these electron-rich alkoxy groups have previously been determined to improve activity [1]. Additionally, a series of compounds with 4-trifluoromethyl substitution (**6**, R_1_ = 4-CF_3_) were targeted for study, to provide contrasting electron-withdrawing effects to the western phenyl ring. Concerning the substituents on the eastern 2-arylcarboxamide fragment (R_2_), a range of common medicinal chemistry substituents will be used, including alkoxy and halogen groups as well as 2,3-disubstitution bulky lipophilic groups as previous studies also suggest these groups are optimal for maximising the anti-proliferative activity [1]. Following synthesis of benzoyl analogues **4**–**6**, these compounds would be reduced to give alcohol derivatives **7**–**9**, as alcohol moieties on the linker have been illustrated to have improved activity over ketones, e.g., enones (**2**) vs. allylic alcohols (**3**) [1].

Ultimately, the aim of this study is to ascertain the optimal length of the linker separating the western phenyl ring from the thienopyridine core in order to refine and optimise the active pharmacophore for this class.

## 2. Results and Discussion

### 2.1. Synthesis of Benzoylthieno[2,3-*b*]Pyridines and Alcohol Derivatives

**Scheme 1:**
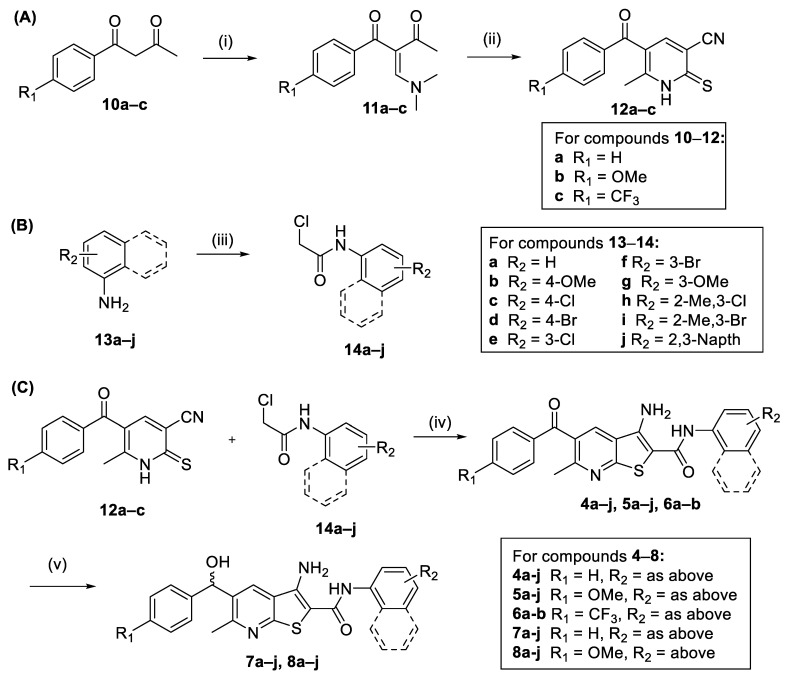
Synthesis of the theino[2,3-*b*]pyridine analogues investigated in this study. (**A**) Synthesis of carbonitrile fragments **12a**–**c**; (i) **10a**–**c** (1 eq.), DMF-DMA (1.2 eq.), l-proline (cat.), 1,4-dioxane, 80 °C, 2 h, 85–91%; (ii) **11a**–**c** (1eq.), 2-cyanothioacetamide (1 eq.), NaH (2 eq.), DMF, r.t., 24 h; 83–91%. (**B**) Synthesis of 2-chloroacetamide fragments **14a**–**j**; (iii) **13a**–**j** (1 eq.), chloroacetyl chloride (1.2 eq.), NEt_3_ (1 eq.), CH_2_Cl_2_, 0 °C, 1 h, 55%-quant. (**C**) Coupling of carbonitriles **12a**–**c** and 2-chloroacetamides **14a**–**j** to give benzoyl analogues **4a**–**j**, **5a**–**j** and **6a**–**b**, followed by reduction to give alcohols **6a**–**j** and **7a**–**j**; (iv) carbonitrile **12a**–**c** (1 eq.), 2-chloroacetamide **14a**–**j** (1 eq.), Na_2_CO_3_ (1.5 eq.), EtOH, reflux, 2 d, 8–66%; and (v) **4a**–**j** or **5a**–**j** (1 eq.), NaBH_4_ (2 eq.), THF/MeOH (5:1), r.t., 2 h, 40%-quant.

The synthetic strategy to access the benzoylthieno[2-3-*b*]pyridines and their alcohol derivatives focused on a convergent synthesis of two distinct regions; carbonitrile fragments **12a**–**c** (A) and an array of 2-chloroacetamide fragments **14a**–**j** (B, Scheme 1). These fragments could then be coupled using previously described methods [1,11], affording the desired library of benzoylthieno[2-3-*b*]pyridines **4a**–**j**, **5a**–**j** and **6a**–**b**, which could then be reduced to give alcohol derivatives **6a**–**j** and **7a**–**j** (C).

Synthesis of carbonitrile fragments began from benzoylacetones **10a**–**c**, which were treated with DMF.DMA and catalytic l-proline in 1,4-dioxane at 80 °C for 2 h to afford enamines **11a**–**c** [12]. Following this, **11a**–**c** were reacted with 2-cyanothioacetamide and NaH to afford the desired carbonitrile fragments **12a**–**c** in 66–91% yield. Carbonitriles **12a**, **12b** and **12c** were isolated as inseparable 5:1, 8:1 and 2:1 mixtures with their respective isomers **12ai**, **12bi** and **12ci**, which were then used as the crude mixture in the following reactions. A proposed mechanism for the synthesis of **12a**–**c** and their respective isomers has been provided in the Supplementary Information (Figure S43).

Next, synthesis of the 2-chloroacetamide fragments **14a**–**j** was achieved in one step using previously described methods [10], via reacting a variety of anilines **13a**–**j** with chloroacetyl chloride and triethylamine, affording **14a**–**j** in yields ranging from 55% to quantitative. Following synthesis of carbonitrile fragments **12a**–**b** and 2-chloroacetamide fragments **14a**–**j** these intermediates were coupled together to afford benzoylthieno [2,3-*b*]pyridines **4a**–**j**, **5a**–**j** in 20–66% yield. The carbonitrile fragments **12a** and **12b** were used in these reactions as mixtures with their isomers **12ai** and **12bi**; however, only the desired products **4a**–**j** and **5a**–**j** were obtained after purification by recrystallization. Two benzoyl analogues **6a**–**b** bearing 4-CF_3_ substituents were also synthesised using carbonitrile **12c**, but it was found that **6a**–**b** were afforded in poor yields of 8–47% and were difficult to purify due to **12c** being in a 2:1 ratio with its isomer. Furthermore, initial biological screening of **6a**,**b** showed these compounds did not demonstrate better activity than **4a**–**b** and **5a**,**b** (see Section 2.2.1), so this 4-CF_3_ substituted series was discontinued.

Finally, the benzoyl motif in **4a**–**j** and **5a**–**j** was reduced using NaBH_4_ in THF/MeOH (5:1) for 2 h, giving desired alcohols **7a**–**j** and **8a**–**j** in 40%-quantitative yields, using extensive aqueous washing as the purification step [13]. Various attempts were made to reduce the ketone in a stereoselective manner via chiral BINAL-H reductants and CBS-reduction conditions [14,15,16]; however, these methods proved to be incompatible with the pro-chiral substrate, and the alcohols **7a**–**j** and **8a**–**j** were tested as racemic mixtures instead. This ultimately concluded the synthesis portion of the study, which resulted in a library of 42 novel thieno [2,3-*b*]pyridine analogues to be assessed for anti-proliferative activity.

### 2.2. Anti-Proliferative Assessment of Novel Thieno[2-3-*b*]Pyridines ***4a**–**j***, ***5a**–**j***, ***6a**,**b***, ***7a**–**j*** and ***8a**–**j***

To determine the anti-proliferative activity of novel benzoylthieno[2,3-*b*]pyridine analogues **4a**–**j**, **5a**–**j**, **6a**–**b**, **7a**–**j** and **8a**–**j**, a ^3^H thymidine incorporation assay was carried out using colorectal cancer (HCT116) and triple-negative breast cancer (MDA-MB-231) cell lines [17]. Identical techniques and cell lines have been utilised to examine the anti-cancer activity of previous thieno[2,3-*b*]pyridine series (**1**–**3**), so this method of assessment would allow direct comparison between these previous iterations and the novel analogues synthesised in this study [1]. Results are reported as a relative percentage of cell growth compared to vehicle following a 1 μM dose with each novel thieno[2,3-*b*]pyridine analogue. Compounds exhibiting greater than 85% inhibition of cell growth in either cell line had their IC_50_ concentrations determined in both cell lines to ascertain which analogues had the most potent dose–response relationship.

#### 2.2.1. Anti-Proliferative Activity of Benzoylthieno[2-3-*b*]Pyridine Analogues **4a**–**j**, **5a**–**j** and **6a**,**b**

The ^3^H thymidine incorporation assay determined that six of the benzoyl analogues were capable of inhibiting HCT116 cell growth by >85% (**4h**, **4i**, **5a**, **5h**, **5i** and **5j**), with four also illustrating the ability to inhibit MDA-MB-231 cell growth by >85% (**5a**, **5h**, **5i** and **5j**) (Table 1).

Regarding the substitution of the phenyl ring on the eastern fragment (R_2_) of the molecule, compounds bearing 2,3 di-substitution such as 2,3-Cl (**h**), 2,3-Br (**i**) and 2,3-naphthyl (**j**) largely demonstrated the greatest anti-proliferative activity. This result is consistent with previous iterations of the thieno[2,3-*b*]pyridine compounds [1,11], further demonstrating that this eastern fragment of the molecular scaffold is highly optimised at this point.

For the western fragment, compounds bearing a *para*-methoxy (R_1_ = OMe) group such as **5a**, **5h**, **5i** and **5j** were more active than their non-substituted counterparts (R_1_ = H), which also agrees with previous observations that alkoxy groups on this ring improve activity [1]. The most potent compound from the benzoyl group was **5i**, which exhibited IC_50_ concentrations of 120–130 nM across both cell lines, followed by **5h** and **5j** with IC_50_ concentrations in the 200–350 nM range. This level of potency is on par with the most potent enone-containing compounds (**2a**) from previous investigations (100–200 nM) [1], suggesting that shortening the linker region does not have a negative effect on the anti-proliferative activity of these thieno[2-3-*b*]pyridine compounds.

#### 2.2.2. Anti-Proliferative Activity of Alcohol Thieno[2-3-*b*]Pyridine Analogues **7a**–**j** and **8a**–**j**

Next, alcohol derivatives **7a**–**j** and **8a**–**j** were assessed for ^3^H thymidine incorporation, and it was immediately apparent that the alcohol-containing analogues had better overall anti-proliferative activity than their benzoyl counterparts (Table 2). Of the 20 alcohol derivatives evaluated, 13 demonstrated >85% inhibition of cell growth in both cell lines and 6 of those compounds (**7h**, **7i**, **8g**, **8h**, **8i** and **8j**) were able to reduce cell growth by >95% in both cell lines. The substituents on the active alcohol derivatives had similar substitution patterns on the eastern 2-arylcarboxamide ring (R_2_) as the benzoyl compounds, with 2,3-disubstitution (**8h**, **8i** and **8j**) again being optimal for activity. An interesting result was compounds bearing 3-methoxy substituents **7g** and **8g** also demonstrated a high degree of growth inhibition despite being inactive in the benzoyl series, which suggests that the alcohol motif may be able to positively influence the binding of these derivatives.

With concern to the dose–response relationship of alcohol derivatives **7a**–**j** and **8a**–**j**, di-substituted 2-arylcarboxamide rings (R_2_ = **h**, **i**, **j**) were once again the most potent inhibitors with IC_50_ concentrations in the 35–225 nM range. Furthermore, the potency of these molecules appeared independent of the substituent on the western phenyl ring, indicating that R_1_ substitution is less important for the activity of alcohol derivatives than it was in the benzoyl series.

The most potent inhibitors regarding activity in both tested cell lines were **7i** and **8h**, which were the only compounds in this study to have IC_50_ concentrations < 100 nM in both cancer cell lines. Compound **7h** also performed well in the MDA-MB-231 cell lines, exhibiting an IC_50_ concentration of 49.9 ± 8.3 nM. It was observed that potent compounds **7h**, **7i** and **8h**, performed similarly to the most potent allylic alcohol derivatives **3a**–**c** [1], further demonstrating that shortening the linker region between the western phenyl ring and the thienopyridine core does not significantly reduce the efficacy of these molecules.

**Figure 3:**
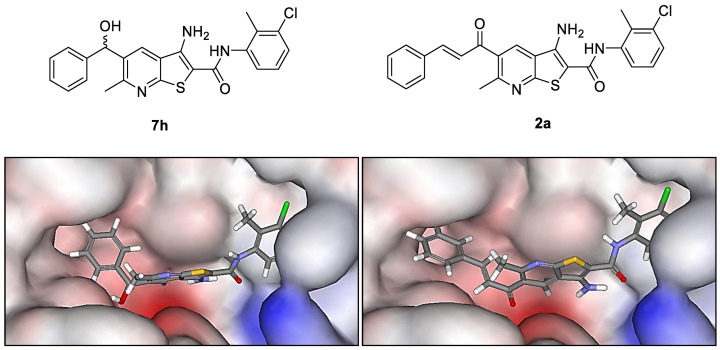

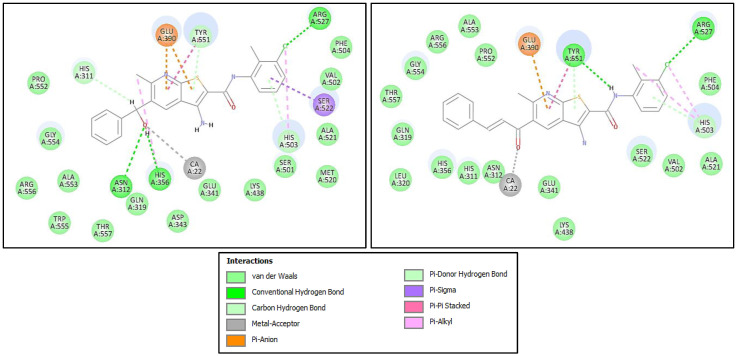
Predicted binding conformation of novel alcohol derivative **7h** (**left**) compared to previously reported cinnamyl compounds **2a** (**right**) in the PLC-δ_1_ binding pocket, showing similar interaction of benzoyl and cinnamyl groups in the lipophilic pocket.

Additionally, molecular docking studies using the PLC-δ_1_ active site (PDB: 1DJX) illustrated that the novel alcohol derivatives from this study bind in a comparable manner to analogous cinnamyl compounds (Figure 3). This includes key interactions to GLU-390 and TYR-551 via the conserved thienopyridine core and to ARG-527 and HIS-503 through the eastern 2-Me,3-Cl phenylcarboxamide ring. Furthermore, the western tethered aryl ring of **7h** shares many of the same lipophilic interactions as compound **2a**, such as van der Waals interactions with ALA-553, GLN-319, PRO-552 and GLY-554. These predicted interactions suggest that the shortened one-atom linker allows similar interactions with the PLC-δ_1_ active as previous thieno[2-3-*b*]pyridine iterations.

Overall this investigation has elucidated that thieno[2-3-*b*]pyridine compounds with shorter one-atom tethers are able to maintain highly potent anti-proliferative activity, opening up greater scope for further exploration into this tether region in future investigations, such as a two-atom acetate or *O*-benzyl tethers. Future investigations into the thieno[2-3-*b*]pyridine pharmacophore could also involve the stereoselective synthesis of alcohols **7h**, **7i** and **8h**, to derive if one enantiomer is more active than the other. This, however, will be a challenging task due to the electron-rich heteroatoms of the planar thienopyridine core coordinating with and disrupting the chiral reducing agents employed. Another potential change could be exploring how *O*-acylation of **7h**, **7i** and **8h** with various esters impacts the activity of these compounds, as recent investigations into *O*-acylation of similar thienopyridines has shown a positive impact on activity through a potential pro-drug pathway [11].

## 3. Materials and Methods

### 3.1. General Experimental Details

All reactions were performed under a nitrogen atmosphere in dry, freshly distilled solvents, unless otherwise noted. Solvents were dried using a solvent purifier (LC Technology Solutions Inc., Salisbury, MA, USA, SP-1 Standalone Solvent Purifier System). Flash chromatography was conducted using Silica Gel 60 (40–63 μm, 230–430 mesh ASTM) with the solvents specified in the experimental procedure for each given compound. Thin layer chromatography (TLC) was performed using Merck silica gel F 254 aluminium plates pre-coated with silica.

All NMR spectra were recorded on a Bruker Avance 400 MHz spectrometer at ambient temperature. Chemical shifts are reported relative to the solvent peak of chloroform (δ 7.26 for ^1^H and δ 77.0 for ^13^C) or DMSO (δ 2.50 for ^1^H and δ 39.5 for ^13^C). ^1^H NMR data are reported as chemical shift (δ), relative integral, multiplicity (s, singlet; d, doublet; dd, doublet of doublets; ddd, doublet of doublet of doublets; t, triplet; m, multiplet; and br, broad peak), coupling constant (*J*, Hz), and the assignment of the atom. ^13^C NMR data are reported as chemical shift (δ) and assignment of the atom. All NMR assignments were performed with HSQC and HMBC experiments. Compounds **4a**–**j**, **5a**–**j**, **6a**,**b**, **7a**–**j** and **8a**–**j** were insoluble in common deuterated solvents such as CDCl_3_, CD_3_OD, (CD_3_)_2_CO and only sparingly soluble in (CD_3_)_2_SO; therefore, it was difficult to obtain high-resolution NMR spectra for all synthesised compounds. The obtained spectra of these compounds are presented in the supplementary information file.

All melting points for solid compounds, given in degrees Celsius (°C), were measured using a Reicher-Kofler block and are uncorrected. Infrared (IR) spectra were recorded using a Perkin-Elmer Spectrum 1000 series Fourier Transform Infrared ATR spectrometer (Perkin Elmer, Waltham, MA, USA). Absorption maxima are expressed in wavenumbers (cm^−1^). Low-resolution and high-resolution mass spectrometry (HRMS) were carried out by either chemical ionization (CI) or electrospray ionization (ESI) on a MicroTOF-Q II mass spectrometer. For low-resolution mass spectrometry, prominent fragments are quoted in the form *a*(*b*) where *a* is the mass to charge ratio of the fragment and *b* is the percentage abundance relative to the base peak. Unless noted, chemical reagents were used as purchased. Experimental methods and full characterisation data for novel compounds, including copies of NMR spectra for all synthesised final compounds that underwent biological assessment can be found in the Supporting Information (Figures S1–S42).

### 3.2. General Synthetic Procedures

#### 3.2.1. General Procedure for Synthesis of Enamines **11a**–**c**

A mixture of 1,3-dicarbonyl **10a**–**c** (6.2 mmol, 1 equiv.), DMF-DMA (1.2 equiv.) and l-proline (0.1 equiv.) in 1,4-dioxane (8 mL) was stirred for 2 h at 80 °C. Following this, the reaction mixture was dried in vacuo and purified using column chromatography (4:1, EtOAc/petroleum ether) to give the desired compounds **11a**–**c**.

#### 3.2.2. General Procedure for Synthesis of Carbonitriles **12a**–**c**

A mixture of 2-cyanothioacetamide (0.5 g, 1 equiv.) and NaH (60% *w*/*w* in mineral oil, 2 equiv.) in DMF (10 mL) was stirred for 10 min at room temperature, followed by the addition of enamine **11a**–**c** (1 equiv.) in DMF (5 mL). The resulting mixture was stirred for 24 h, then acidified to pH 2–4 using 2M HCl and the resultant precipitate was collected using vacuum filtration affording a mixture of carbonitriles **12a**–**c** and their respective isomers **12ai**–**12ci**.

#### 3.2.3. General Procedure for Synthesis of 2-Chloroacetamides **14a**–**j**

Chloroacetyl chloride (1.2 equiv.) was added, dropwise over 15 min at 0 °C, to a solution of aniline **13a**–**j** (6 mmol, 1 equiv.) and NEt_3_ (1 equiv.) in CH_2_Cl_2_ (30 mL). The mixture was stirred for 1 h at 0 °C, then left overnight at room temperature. Following this, the reaction mixture was diluted with CH_2_Cl_2_, washed with 2M HCl (2 × 15 mL), H_2_O (20 mL), sat. aq. NaHCO_3_ (20 mL) and brine (15 mL) and then dried with Na_2_SO_4_. The solvent was removed under reduced pressure to give the desired 2-chloroacetamides **14a**–**j**, with no further purification required.

#### 3.2.4. General Procedure for Synthesis of Benzoyl Thieno[2-3-*b*]Pyridine Derivatives **4a**–**j**, **5a**–**j** and **6a**–**b**

A mixture of carbonitrile **10a**–**c** (0.4 mmol, 1 equiv.), chloroacetamide **12a**–**j** (1 equiv.) and Na_2_CO_3_ (1.5 equiv.) in EtOH (3 mL) was heated at reflux for two days. The mixture was then dried in vacuo to give a crude product which was recrystallised from MeOH to give benzoylthieno[2,3-*b*]pyridine derivatives **4a**–**j**, **5a**–**j** and **6a**–**b**.

#### 3.2.5. General Procedure for Synthesis of Alcohol Thieno[2-3-*b*]Pyridine Derivatives **7a**–**j** and **8a**–**j**

Sodium borohydride (0.01 g, 2 equiv.) was added to a solution of **4a**–**j** and **5a**–**j** (1 equiv.) in THF/MeOH (4:1, 6 mL). The mixture was stirred at room temperature for 2 h, and then water (2.5 mL) was added and extracted with ethyl acetate (3 × 20 mL). The combined organic extracts were then washed with H_2_O (10 mL) and brine (15 mL) and dried with MgSO_4_ to give the desired alcohols **7a**–**j** and **8a**–**j**, with no further purification required. This procedure has been previously shown to remove borohydride by-products and provide high-purity samples of structurally similar thieno[2-3-*b*]pyridines [11].

### 3.3. Cell Proliferation Testing

The synthesised thieno[2,3-*b*]pyridines **4a**–**j**, **5a**–**j**, **6a**,**b**, **7a**–**j** and **8a**–**j** were measured for their anti-proliferative activity using triple negative breast cancer MDA-MB-231 and colorectal cancer HCT-116 cell lines in a ^3^H thymidine incorporation assay. Compounds assessed were found to be >90% pure prior to biological testing. The method was used as previously described by Rees et al. and Leung et al. [17,18]. Briefly, cell lines were purchased from the American Type Culture Collection (ATCC), Manassas, VA, USA. Cells were grown in α-MEM containing 5% foetal bovine serum (FBS). Insulin/transferrin/selenium supplements were contained in all growth media, added according to the manufacturer’s instructions (Roche), as well as penicillin (100 U/mL) and streptomycin (100 μg/mL). Experiments were all performed on cells grown in their respective growth media.

#### Cell Proliferation Assay

~3000 cells were seeded in each well and incubated with thieno[2,3-*b*]pyridines for 72 h at 1 μM for single concentration testing and at varying concentrations for dose–response analysis. The experiments were using 0.04 µCi of ^3^H thymidine, which was added to each well 5 h prior to harvest. The cells were then harvested onto glass fibre filters using an automated TomTec harvester. The filters were incubated with Betaplate Scint, and thymidine incorporation was determined with a Trilux/Betaplate counter. The effects of the inhibitors on the incorporation of ^3^H thymidine into DNA were determined relative to the control samples, with the positive control being a previously known active compound (inhibitor **7n** from Reference 16) [19] and the negative control being cells treated with vehicle only. All experiments were performed three times using triplicate wells (Figures S44–S46).

### 3.4. Molecular Docking Experiments

Molecular docking experiments were performed using the mammalian PLC-δ1 crystal structure, which was obtained from the Protein Data Bank (PDB ID: 1DJX) with a resolution of 2.3 Å [20]. This PLC-δ_1_ crystal structure was used as the docking scaffold as it has previously shown utility at identifying novel PI-PLC inhibitors [21]. The GOLD suite (Version 2022.3.0) was used to prepare the crystal structure for docking by the addition of hydrogen atoms and removal of the co-crystallised ligand (D-myo-inositol-1,4,5-triphosphate, IP3). Basic amino acids were assumed to be protonated, and acidic amino acids deprotonated to closely resemble the physiological environment. The coordinates of the binding pocket were X = 126.257, Y = 38.394 and Z = 22.370, with a 10 Å radius. For each docked ligand, fifty docking runs were performed at 100% search efficiency using the ChemPLP algorithm to predict the optimal binding conformation. The ChemPLP algorithm was chosen as it has been demonstrated to be the most accurate scoring function in the GOLD suite for predicting the correct ligand binding pose [22].

## 4. Conclusions

In this study, a series of 20 benzoyl-containing thieno[2-3-*b*]pyridine compounds, with various substituents on the two aromatic rings present on the molecular scaffold, and their corresponding alcohol derivatives were synthesised. This library of 40 analogues was assessed for growth inhibition in HCT116 and MDA-MB-231 cancer cell lines, and it was found that the alcohol derivatives demonstrated more anti-proliferative activity than the benzoyl-containing compounds. The most potent inhibitor was **7i** with an IC_50_ concentrations of 31.6 ± 0.8 nM and 35.8 ± 0.8 nM in HCT116 and MDA-MB-231 cell lines, respectively. Compounds **7i** and **8h** also exhibited potent IC_50_ concentrations, further demonstrating that a 2-methyl-3-halogen substitution pattern on the eastern phenyl ring is optimal for this region of the pharmacophore. For the western phenyl ring, the presence of a 4-methoxy substituent improved the activity of benzoyl derivatives but had a negligible effect on the activity of alcohol derivatives. When comparing **7h** and **7i** to their most potent enone and allylic alcohol counterparts, the IC_50_ concentrations were similar, in the range of 25–50 nM. Overall, this study suggests that the benzoyl and alcohol derivatives **4a**–**j**, **5a**–**j**, **6a**,**b**, **7a**–**j** and **8a**–**j** explored in this study with shorter linker regions were comparable in their activity and were able to be afforded using a more straightforward synthetic protocol requiring less steps to synthesise than previous thieno[2-3-a]pyridines.

## Figures and Tables

**Table 1:** Relative ^3^H thymidine uptake by HCT116 and MDA-MB-231 cells after treatment with novel benzoyl analogues **4a**–**j**, **5a**–**j** and **6a**,**b** at 1 μM. IC_50_ concentrations were also reported for the most active compounds. Cinnamyl compounds (**2a**–**c**) are included for comparison [1]. Values are reported as mean ± s.e.m. of three individual experiments performed in triplicate.

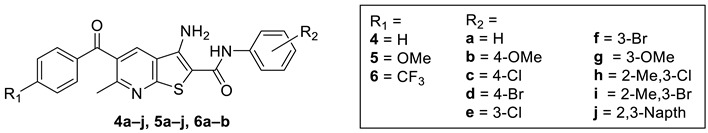				
Compound	Mean Relative Growth (%) 1 μM		IC_50_ (nM)	
	HCT-116	MDA-MB-231	HCT-116	MDA-MB-231
**4a**	116.8 ± 14.5	106.6 ± 11.2	-	-
**4b**	114.4 ± 6.1	108.4 ± 13.3	-	-
**4c**	113.9 ± 5.7	107.5 ± 6.2	-	-
**4d**	112.0 ± 3.1	109.4 ± 2.5	-	-
**4e**	109.7 ± 1.3	105.4 ± 1.2	-	-
**4f**	108.4 ± 3.6	106.3 ± 1.0	-	-
**4g**	108.9 ± 0.1	106.6 ± 1.3	-	-
**4h**	5.3 ± 1.0	20.4 ± 1.7	497.8 ± 10.9	541.8 ± 7.6
**4i**	5.3 ± 2.0	28.6 ± 6.4	513.3 ± 4.7	598.6 ± 64.4
**4j**	122.6 ± 17.8	108.7 ± 4.7	-	-
**5a**	6.4 ± 1.7	12.8 ± 1.9	719.4 ± 227.7	709.5 ± 162.5
**5b**	106.9 ± 5.0	106.9 ± 1.4	-	-
**5c**	107.2 ± 3.5	103.7 ± 6.7	-	-
**5d**	108.4 ± 1.7	96.9 ± 0.1	-	-
**5e**	96.9 ± 1.4	102.9 ± 4.1	-	-
**5f**	104.1 ± 1.3	105.8 ± 3.6	-	-
**5g**	116.3 ± 11.8	105.4 ± 9.5	-	-
**5h**	3.4 ± 1.2	10.2 ± 2.9	236.8 ± 98.0	303.5 ± 64.5
**5i**	1.7 ± 0.8	4.4 ± 0.1	120.9 ± 2.0	128.6 ± 5.2
**5j**	2.0 ± 0.1	7.6 ± 1.6	253.0 ± 115.8	353.9 ± 84.9
**6a**	101.8 ± 3.5	96.6 ± 3.0	-	-
**6b**	96.9 ± 6.0	97.3 ± 1.6	-	-
Examples of highly active cinnamyl compounds (**2a**–**c**) from previous generation [1]: 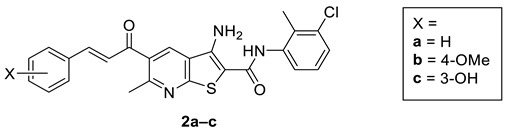				
**2a**	3.0	5.8	154	182
**2b**	1.6	2.6	461	436
**2c**	1.4	2.0	137	103

**Table 2:** Relative ^3^H thymidine uptake by HCT116 and MDA-MB-231 cells following treatment with novel alcohol derivatives **7a**–**j** and **8a**–**j** at 1 μM. Allylic alcohols (**3a**–**c**) are included for comparison [1]. IC_50_ concentrations were determined and reported for the compounds with the highest growth inhibition. Values are reported as mean ± s.e.m. of three individual experiments performed in triplicate.

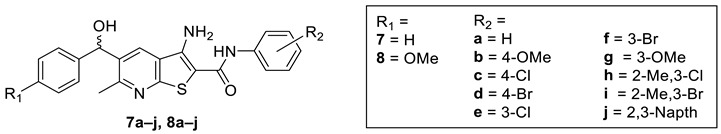				
Compound	Mean Relative Growth (%) 1 μM		IC50 (nM)	
	HCT-116	MDA-MB-231	HCT-116	MDA-MB-231
**7a**	3.0 ± 0.5	10.7 ± 2.8	468.1 ± 1.9	453.9 ± 3.2
**7b**	108.9 ± 9.7	104.6 ± 0.8	-	-
**7c**	105.5 ± 7.5	99.6 ± 7.5	-	-
**7d**	107.9 ± 5.3	101.4 ± 2.6	-	-
**7e**	107.9 ± 5.2	105.1 ± 1.3	-	-
**7f**	97.4 ± 10.3	87.8 ± 7.9	-	-
**7g**	3.7 ± 1.4	11.8 ± 2.6	193.1 ± 14.4	139.7 ± 30.7
**7h**	2.0 ± 0.8	4.8 ± 0.5	49.9 ± 8.3	111.1 ± 4.9
**7i**	0.6 ± 0.2	3.4 ± 0.1	31.6 ± 0.8	35.8 ± 0.8
**7j**	1.9 ± 0.5	4.7 ± 0.5	125.6 ± 3.8	157.8 ± 17.8
**8a**	0.8 ± 0.2	5.4 ± 0.2	122.3 ± 5.7	124.0 ± 12.0
**8b**	2.9 ± 0.4	14.3 ± 1.3	455.7 ± 43.9	591.0 ± 135.8
**8c**	97.6 ± 1.9	83.3 ± 1.3	-	-
**8d**	99.2 ± 0.5	89.1 ± 3.1	-	-
**8e**	1.5 ± 0.2	13.7 ± 0.9	378.3 ± 81.8	540.0 ± 16.3
**8f**	0.6 ± 0.1	8.1 ± 0.3	344.3 ± 82.6	551.3 ± 48.2
**8g**	0.4 ± 0.1	3.3 ± 0.4	111.0 ± 6.7	111.0 ± 12.5
**8h**	0.4 ± 0.1	3.0 ± 0.3	69.5 ± 11.5	78.6 ± 38.9
**8i**	0.2 ± 0.1	2.3 ± 0.3	51.0 ± 21.5	222.0 ± 119.1
**8j**	0.2 ± 0.1	2.4 ± 0.1	103.0 ± 19.0	119.5 ± 19.5
Examples of highly active allylic alcohols (3) from previous generation [1]: 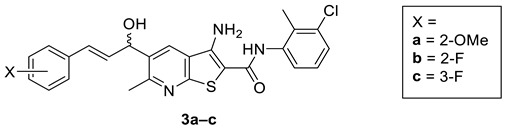				
**3a**	1.6	3.9	55	92
**3b**	1.1	2.9	32	36
**3c**	1.8	4.5	50	104

## Data Availability

The data are contained within the article.

## Supplementary Materials

The supporting information can be downloaded at: https://www.mdpi.com/article/10.3390/ijms241411407/s1, references [23,24,25,26,27,28] are cited in Supplementary Materials.

## References

[1] Haverkate N.A., Leung E., Pilkington L.I., Barker D. (2021). Tethered Aryl Groups Increase the Activity of Anti-Proliferative Thieno[2-3-*b*]Pyridines by Targeting a Lipophilic Region in the Active Site of PI-PLC. Pharmaceutics.

[2] Pervan M., Marijan S., Markotić A., Pilkington L.I., Haverkate N.A., Barker D., Reynisson J., Meić L., Radan M., Čikeš Čulić V. (2022). Novel Thieno [2-3-*b*]Pyridine Anticancer Compound Lowers Cancer Stem Cell Fraction Inducing Shift of Lipid to Glucose Metabolism. Int. J. Mol. Sci..

[3] Marijan S., Markotić A., Mastelić A., Režić-Mužinić N., Pilkington L.I., Reynisson J., Čulić V.Č. (2020). Glycosphingolipid Expression at Breast Cancer Stem Cells after Novel Thieno[2-3-*b*]Pyridine Anticancer Compound Treatment. Sci. Rep..

[4] Reynisson J., Jaiswal J.K., Barker D., D’Mello S.A.N., Denny W.A., Baguley B.C., Leung E.Y. (2016). Evidence That Phospholipase C Is Involved in the Antitumour Action of NSC768313, a New Thieno[2-3-*b*]Pyridine Derivative. Cancer Cell Int..

[5] Arabshahi H.J., van Rensburg M., Pilkington L.I., Jeon C.Y., Song M., Gridel L.-M., Leung E., Barker D., Vuica-Ross M., Volcho K.P. (2015). A Synthesis, in Silico, in Vitro and in Vivo Study of Thieno[2-3-*b*]Pyridine Anticancer Analogues. Med. Chem. Commun..

[6] Bernlochner I., Sibbing D., Gresele P., Born G.V.R., Patrono C., Page C.P. (2012). Thienopyridines and Other ADP-Receptor Antagonists. Antiplatelet Agents.

[7] Katritch V., Jaakola V.-P., Lane J.R., Lin J., IJzerman A.P., Yeager M., Kufareva I., Stevens R.C., Abagyan R. (2010). Structure-Based Discovery of Novel Chemotypes for Adenosine A2A Receptor Antagonists. J. Med. Chem..

[8] Eurtivong C., Semenov V., Semenova M., Konyushkin L., Atamanenko O., Reynisson J., Kiselyov A. (2017). 3-Amino-Thieno[2-3-*b*]Pyridines as Microtubule-Destabilising Agents: Molecular Modelling and Biological Evaluation in the Sea Urchin Embryo and Human Cancer Cells. Bioorg. Med. Chem..

[9] Romagnoli R., Baraldi P.G., Kimatrai Salvador M., Preti D., Aghazadeh Tabrizi M., Bassetto M., Brancale A., Hamel E., Castagliuolo I., Bortolozzi R. (2013). Synthesis and Biological Evaluation of 2-(Alkoxycarbonyl)-3-Anilinobenzo[b]Thiophenes and Thieno[2-3-*b*]Pyridines as New Potent Anticancer Agents. J. Med. Chem..

[10] van Rensburg M., Leung E., Haverkate N.A., Eurtivong C., Pilkington L.I., Reynisson J., Barker D. (2017). Synthesis and Antiproliferative Activity of 2-Chlorophenyl Carboxamide Thienopyridines. Bioorg. Med. Chem. Lett..

[11] Haverkate N.A., Leung E., Pilkington L.I., Barker D. (2022). Disruption of Crystal Packing in Thieno[2-3-*b*]Pyridines Improves Anti-Proliferative Activity. Molecules.

[12] Kumar D., Kommi D.N., Chopra P., Ansari M.I., Chakraborti A.K. (2012). L-Proline-Catalyzed Activation of Methyl Ketones or Active Methylene Compounds and DMF-DMA for Syntheses of (2E)-3-Dimethylamino-2- Propen-1-Ones. Eur. J. Org. Chem..

[13] Li D.-Y., Lou Y.-J., Xu J., Chen X.-Y., Lin X.-F., Wu Q. (2021). Electronic Effect-Guided Rational Design of Candida Antarctica Lipase B for Kinetic Resolution Towards Diarylmethanols. Adv. Synth. Catal..

[14] Noyori R., Tomino I., Tanimoto Y., Nishizawa M. (1984). Asymmetric Synthesis via Axially Dissymmetric Molecules. 6. Rational Designing of Efficient Chiral Reducing Agents. Highly Enantioselective Reduction of Aromatic Ketones by Binaphthol-Modified Lithium Aluminium Hydride Reagents. J. Am. Chem. Soc..

[15] Noyori R., Tomino I., Yamada M., Nishizawa M. (1984). Asymmetric Synthesis via Axially Dissymmetric Molecules. 7. Synthetic Applications of the Enantioselective Reduction by Binaphthol-Modified Lithium Aluminium Hydride Reagents. J. Am. Chem. Soc..

[16] Corey E.J., Helal C.J. (1998). Reduction of Carbonyl Compounds with Chiral Oxazaborolidine Catalysts: A New Paradigm for Enantioselective Catalysis and a Powerful New Synthetic Method. Angew. Chem. Int. Ed..

[17] Rees S.W.P., Leung E., Reynisson J., Barker D., Pilkington L.I. (2021). Development of 2-Morpholino-*N*-Hydroxybenzamides as Anti-Proliferative PC-PLC Inhibitors. Bioorg. Chem..

[18] Leung E., Kim J.E., Rewcastle G.W., Finlay G.J., Baguley B.C. (2011). Comparison of the Effects of the PI3K/MTOR Inhibitors NVP-BEZ235 and GSK2126458 on Tamoxifen-Resistant Breast Cancer Cells. Cancer Biol. Ther..

[19] Leung E., Pilkington L.I., van Rensburg M., Jeon C.Y., Song M., Arabshahi H.J., De Zoysa G.H., Sarojini V., Denny W.A., Reynisson J. (2016). Synthesis and Cytotoxicity of Thieno[2-3-*b*]Quinoline-2-Carboxamide and Cycloalkyl[b]Thieno[3,2-e]Pyridine-2-Carboxamide Derivatives. Bioorg. Med. Chem..

[20] Essen L.-O., Perisic O., Katan M., Wu Y., Roberts M.F., Williams R.L. (1997). Structural Mapping of the Catalytic Mechanism for a Mammalian Phosphoinositide-Specific Phospholipase C. Biochemistry.

[21] Reynisson J., Court W., O’Neill C., Day J., Patterson L., McDonald E., Workman P., Katan M., Eccles S.A. (2009). The Identification of Novel PLC-γ Inhibitors Using Virtual High Throughput Screening. Bioorg. Med. Chem..

[22] Liebeschuetz J.W., Cole J.C., Korb O. (2012). Pose Prediction and Virtual Screening Performance of GOLD Scoring Functions in a Standardized Test. J. Comput. Aided Mol. Des..

[23] Obydennov D.L., Goncharov A.O., Sosnovskikh V.Y. (2016). Preparative Synthesis of Ethyl 5-Acyl-4-Pyrone-2-Carboxylates and 6-Aryl-, 6-Alkyl-, and 5-Acylcomanic Acids on Their Basis. Russ. Chem. Bull..

[24] Abu-Shanab F.A., Hessen A.M., Mousa S.a.S. (2007). Dimethylformamide Dimethyl Acetal in Heterocyclic Synthesis: Synthesis of Polyfunctionally Substituted Pyridine Derivatives as Precursors to Bicycles and Polycycles. J. Heterocycl. Chem..

[25] Baraldi P.G., Preti D., Tabrizi M.A., Fruttarolo F., Saponaro G., Baraldi S., Romagnoli R., Moorman A.R., Gessi S., Varani K. (2007). N6-[(Hetero)Aryl/(Cyclo)Alkyl-Carbamoyl-Methoxy-Phenyl]-(2-Chloro)-5′-N-Ethylcarboxamido-Adenosines: The First Example of Adenosine-Related Structures with Potent Agonist Activity at the Human A2B Adenosine Receptor. Bioorg. Med. Chem..

[26] Pace V., Castoldi L., Holzer W. (2013). Addition of Lithium Carbenoids to Isocyanates: A Direct Access to Synthetically Useful N-Substituted 2-Haloacetamides. Chem. Commun..

[27] Wang G.-B., Wang L.-F., Li C.-Z., Sun J., Zhou G.-M., Yang D.-C. (2012). A Facile and Efficient Method for the Selective Deacylation of N-Arylacetamides and 2-Chloro-N-Arylacetamides Catalyzed by SOCl2. Res. Chem. Intermed..

[28] Ghosh K., Sen T. (2010). Naphthalene Appended 2,5-Diketopiperazine towards Fluorometric Response of Dihydrogenphosphate. J. Incl. Phenom. Macrocycl. Chem..

