# Supplementary material for: Synthesis and Anti-Proliferative Activity of 5-Benzoyl and 5-Benzylhydroxy Derivatives of 3-Amino-2-Arylcarboxamido-Thieno[2-3-b]Pyridines
Source: Int J Mol Sci. 2023 Jul 13;24(14):11407. doi: 10.3390/ijms241411407 (PMC10380547; doi:10.3390/ijms241411407)
Supplement: Supplementary file 1 [file ijms-24-11407-s001.zip › ijms-2448661-supplementary.pdf]

Supporting Information for:

Synthesis and anti-proliferative activity of 5-benzoyl and 5-benzylhydroxy derivatives of 3-amino-2-arylcarboxamido-thieno[2-3-*b*]pyridines.

Bailey Morphet[1], Shaun W. P. Rees[1], Natalie A. Haverkate[1], Hamid Aziz[2,3], Euphemia Leung[4], Lisa I. Pilkington[1,5], David Barker[1,6*].*

[1]School of Chemical Sciences, University of Auckland, Auckland 1010, New Zealand.

[2]Department of Chemistry, Quaid-I-Azam University, Islamabad 45320, Pakistan.

[3] Department of Chemistry, Rawalpindi Women University, Rawalpindi Pakistan.

[4]Auckland Cancer Society Research Centre and Department of Molecular Medicine and Pathology, University of Auckland, Grafton, Auckland 1023, New Zealand.

[5]Te Pūnaha Matatini, Auckland 1142, New Zealand.

[6]The MacDiarmid Institute for Advanced Materials and Nanotechnology, Victoria University of Wellington, Wellington 6012, New Zealand.

Corresponding authors: lisa.pilkington@auckland.ac.nz; d.barker@auckland.ac.nz.

Table of Contents:

Synthetic procedures and compound characterisation data S2 – S52

^{1}H and ^{13}C NMR spectra for **4a-j, 5a-j, 6a-b, 7a-j** and **8a-j** S53 – S94

Proposed mechanism for the formation of **12a-c** and **12ai-ci** S95

Dose-Response curves for compounds **5i**, **7h**, **7i** and **8h**. S96

References S98

Synthetic procedures and compound characterisation data:

(*E*)-2-((Dimethylamino)methylene)-1-phenylbutane-1,3-dione 11a

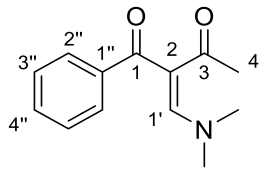

The reaction was carried out following General procedure 3.2.1 using 1,3 di-carbonyl **10a** (1.0 g, 6.17 mmol), L-proline (71 mg, 0.617 mmol) and DMF-DMA (0.98 mL, 7.4 mmol) in 1,4-dioxane (8 mL) to give the *title compound* **11a** (1.04 g, 78%) as a red oil. R_f = 0.13 (4:1 EtOAc/Petroleum ether). δ_H (400 MHz, $(CD_3)_2SO$) 2.05 (3H, s, H-4), 2.73-2.89 (6H, br s, $N(CH_3)_2$), 7.46 (2H, tt, *J* = 8.1, 1.4 Hz, H-3"), 7.58 (1H, tt, *J* = 7.3, 1.6 Hz, H-4"), 7.70-7.73 (3H, m, H-1' and H-2"). The ^{1}H NMR data was in agreement with the literature values.[1]

(*E*)-2-((Dimethylamino)methylene)-1-(4'-methoxyphenyl)butane-1,3-dione 11b

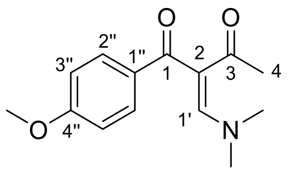

The reaction was carried out following General procedure 3.2.1 using 1,3 di-carbonyl **10b** (1.38 g, 7.18 mmol), L-proline (83 mg, 0.718 mmol) and DMF-DMA (1.14 mL, 8.62 mmol) in 1,4-dioxane (10 mL) to give the *title compound* **11b** (1.51 g, 85%), as an orange solid. m.p. 124-126 °C (Lit. 126-127 °C).[1] R_f = 0.41 (19:1 CH_2Cl_2/MeOH). δ_H (400 MHz, $(CD_3)_2SO$) 2.02 (3H, s, H-4), 2.72-2.89 (6H, br s, $N(CH_3)_2$), 3.82 (3H, s, 4"-OCH_3), 7.00 (2H, dt, *J* = 8.9, 2.4 Hz, H-2" or H-3"), 7.65 (1H, s, H-1'), 7.71(2H, dt, *J* = 8.9, 2.4 Hz, H-2" or H-3"). The ^{1}H NMR data agreed with the literature values.[1]

(*E*)-2-((Dimethylamino)methylene)-1-(4'-(trifluoromethyl)phenyl)butane-1,3-dione 11c

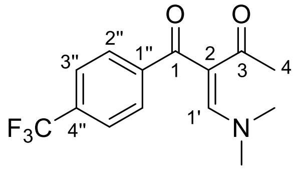

The reaction was carried out following General procedure 3.2.1 using 1,3 di-carbonyl **10c** (2.15 g, 9.32 mmol) and L-proline (0.107 g, 0.932 mmol) in DMF-DMA (1.63 mL, 11.2 mmol) in 1,4-dioxane (10 mL) to give the *title compound* **11c** (1.03 g, 39 %) as a red oil. R_f = 0.42 (19:1, CH_2Cl_2/MeOH). δ_H (400 MHz, $(CD_3)_2SO$) 2.11 (3H, s, H-4), 2.73-2.89 (6H, br s, $N(CH_3)_2$), 7.78 (1H, s, H-1'), 7.80 (2H, d, *J* = 8.4 Hz, H-3''), 7.86 (2H, d, *J* = 8.4 Hz, H-2''). δ_C (100 MHz, $(CD_3)_2SO$) 27.3 (C-4), 110.8 (C-2), 125.4 (C-3''), 129.2 (C-2''), 143.6 (C-1''), 156.9 (C-1'), 194.3 (C-1), 194.4 (C-3).

5-Benzoyl-6-methyl-2-thioxo-1,2-dihydropyridine-3-carbonitrile 12a

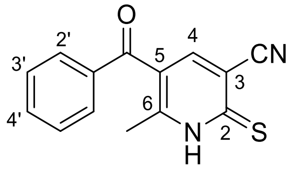

The reaction was carried out following General procedure 3.2.2 using enamine **11a** (0.974 g, 4.48 mmol), sodium hydride (60% w/w in mineral oil, 0.36 g, 8.96 mmol) and 2-cyanothioacetamide (0.45 g, 4.48 mmol) in DMF (15 mL) to give the *title compound* **12a** (0.95 g, 83%) as a brown solid. δ_H (400 MHz, $(CD_3)_2SO$) 2.45 (3H, s, 6-CH_3), 7.56 (2H, t, *J* = 7.9 Hz, H-3'), 7.70 (1H, d, *J* = 7.9 Hz, H-4'), 7.78 (2H, d, *J* = 7.9 Hz, H-2'), 8.03 (1H, s, H-4), 14.39 (1H, br s, NH). The ^1H NMR data was in agreement with the literature values.[2]

5-(4'-Methoxybenzoyl)-6-methyl-2-thioxo-1,2-dihydropyridine-3-carbonitrile 12b

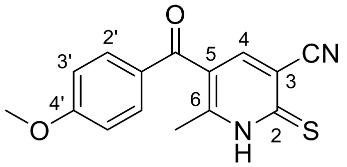

The reaction was carried out following General procedure 3.2.2 using enamine **11b** (0.808 g, 3.27 mmol), sodium hydride (60% w/w in mineral oil, 0.26 g, 6.54 mmol) and 2-cyanothioacetamide (0.33 g, 3.27 mmol) in DMF (12.5 mL) to give the *title compound* **12b** (0.84 g, 91%) as a brown solid. δ_H (400 MHz, $(CD_3)_2SO$) 2.40 (3H, s, 6-CH_3), 3.86 (3H, s, 4'-OCH_3), 7.08 (2H, d, *J* = 8.8 Hz, H-3'), 7.78 (2H, dt, *J* = 8.8, 2.4 Hz, H-2'), 8.03 (1H, s, H-4), 14.35 (1H, br s, NH). δ_C (100 MHz, $(CD_3)_2SO$) 18.2 (6-CH_3), 55.7 (4'-OCH_3), 113.1 (C-3), 113.8 (C-3'), 116.6 (CN), 122.6 (C-5), 129.2 (C-1'), 132.3 (C-2'), 144.1 (C-4), 155.3 (C-6), 163.7 (C-4'), 178.7 (C-2), 190.5 (5-CO). ν_{max} (ATR)/cm^{-1} 3373 (N-H amine), 2929 (C-H alkane), 2224 (CN), 1646 (C=O carbonyl), 1591 (C=C aromatic), 1437 (-C-H bending), 1247 (C-N aromatic), 1178 (C-O ether). *m/z* (ESI^+): 307 (MNa^+, 100%), 285 (13%), 259 (30%), 227 (22%), 159 (14%), 128 (10%), 101 (34%). HRMS (ESI^+) found (MNa^+): 307.0504 $C_{15}H_{12}N_2NaOS$ requires 307.0512.

6-Methyl-2-thioxo-5-(4'-(trifluoromethyl)benzoyl)-1,2-dihydropyridine-3-carbonitrile 12c

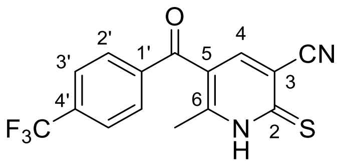

The reaction was carried out following General procedure 3.2.2 using enamine **11c** (1.03 g, 3.62 mmol), sodium hydride (60% w/w in mineral oil, 0.28 g, 7.25 mmol) and 2-cyanothioacetamide (0.36 g, 3.62 mmol) in DMF (10 mL) to give the *title compound* **12c** (0.77 g, 66 %) as a brown solid. m.p. decomp. at 194 °C. ν_{max} (ATR)/cm^{-1} 3166 (N-H amine), 2927 (C-H alkane), 2225 (CN), 1646 (C=O carbonyl), 1579 (C=C aromatic), 1436 (-C-H bending), 1321 (C-F), 1246 (C-N aromatic). δ_H (400 MHz, $(CD_3)_2SO$) 2.51 (3H, s, 6-CH_3), 7.88-7.94 (4H, d, *J* = 8.7 Hz, H-2' and H-3'), 8.03 (1H, s, H-4), 14.4 (1H, br s, NH). δ_C (100 MHz, $(CD_3)_2SO$) 18.6 (6-CH_3), 113.1 (C-3), 116.4 (CN), 125.8 (C-3'), 121.5 (C-5), 130.4 (C-2'), 140.7 (C-1'), 144.6 (C-4), 157.1 (C-6), 179.3 (C-2), 191.23 (5-CO). C-4'', and 4''-CF_3 not observed. *m/z* (ESI^+): 345 (MNa^+, 73%), 280 (82%), 229 (100%), 179 (77%), 165 (65%), 142 (69%). HRMS (ESI^+) found (MNa^+): 345.0289 $C_{15}H_9F_3N_2NaOS$ requires 345.0291.

2-Chloro-*N*-phenylacetamide 14a

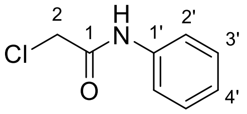

The reaction was carried out following General procedure 3.2.3 using aniline **13a** (1.46 mL, 16.0 mmol), Et_3N (2.23 mL, 16.0 mmol) and chloroacetyl chloride (1.53 mL, 19.2 mmol) in CH_2Cl_2 (80 mL) to give the *title compound* **14a** (2.59 g, 90%) as a white solid. m.p. 135-137 °C (Lit. 134-137 °C).[3] δ_H (400 MHz, $CDCl_3$) 4.21 (2H, s, H-2), 7.19 (1H, t, *J* = 7.6 Hz, H-4'), 7.39 (2H, t, *J* = 7.8 Hz, H-3'), 7.55 (2H, d, *J* = 7.6 Hz, H-2') and 8.24 (1H, br s, NH). The ^{1}H NMR data was equivalent to the literature values.[3]

2-chloro-N-(4-methoxyphenyl)acetamide 14b

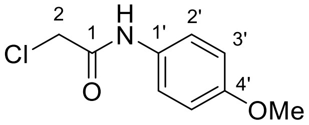

The reaction was carried out following General procedure 3.2.3 using aniline **13b** (0.54 mL, 4.67 mmol), Et_3N (0.65 mL, 4.67 mmol) and chloroacetyl chloride (0.45 mL, 5.6 mmol) in CH_2Cl_2 (23.2 mL) to give the *title compound* **14b** (0.82 g, 96%) as a beige solid. m.p. 169-170 °C (Lit. 163-165 °C).[3] δ_H (400 MHz, $CDCl_3$) 3.80 (3H, s, 4'-OMe), 4.17 (2H, s, H-2), 6.88 (2H, d, *J* = 8.5 Hz, H-3'), 7.44 (2H, d, *J* = 8.5 Hz, H-2'), 8.22 (1H, br s, NH). The ^{1}H NMR data agreed with the literature values.[3]

2-Chloro-*N*-(4'-chlorophenyl)acetamide 14c

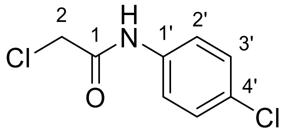

The reaction was carried out following General procedure 3.2.3 using aniline **13c** (0.98 mL, 7.84 mmol), Et_3N (1.09 mL, 7.84 mmol) and chloroacetyl chloride (0.75 mL, 9.41 mmol) in CH_2Cl_2 (39 mL) to give the *title compound* **14c** (0.88 g, 55%) as a brown solid. m.p. 146-148 °C (Lit. 170-171 °C).[3] δ_H (400 MHz, $CDCl_3$) 4.19 (2H, s, H-2), 7.32 (2H, dt, *J* = 8.9, 2.5 Hz, H-2' or H-3'), 7.51 (2H, dt, *J* = 8.9, 2.5 Hz, H-2' or H-3'), 8.22 (1H, br s, NH). The ^{1}H NMR data agreed with the literature values.[3]

2-Chloro-*N*-(4'-bromophenyl)acetamide 14d

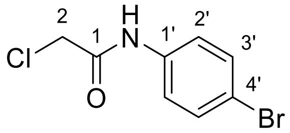

The reaction was carried out following General procedure 3.2.3 using aniline **13d** (0.67 mL, 5.81 mmol), Et_3N (0.81 mL, 5.81 mmol) and chloroacetyl chloride (0.56 mL, 6.98 mmol) in CH_2Cl_2 (28.9 mL) to give the *title compound* **14d** (1.25 g, 98%) as a light yellow solid. m.p. 181-183 °C (Lit. 180 °C).[3] δ_H (400 MHz, $CDCl_3$) 4.19 (2H, s, H-2), 7.44-7.49 (4H, m, H-2' and H-3'), 8.21 (1H, br s, NH). The ^{1}H NMR data was in agreement with the literature values.[3]

2-Chloro-*N*-(3'-chlorophenyl)acetamide 14e

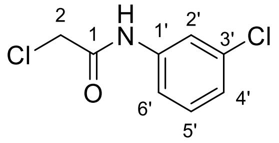

The reaction was carried out following General procedure 3.2.3 using aniline **12e** (0.83 mL, 7.84 mmol), Et_3N (1.09 mL, 7.84 mmol) and chloroacetyl chloride (0.75 mL, 9.4 mmol) in CH_2Cl_2 (39 mL) to give the *title compound* **14e** (1.32 g, 83%) as a white solid. m.p. 100-101 °C (Lit. 100°C).[4] δ_H (400 MHz, $CDCl_3$) 4.19 (2H, s, H-2), 7.15 (1H, ddd, *J* = 8.1, 2.1, 0.9 Hz, H-4' or H-6'), 7.28 (1H, t, *J* = 8.1 Hz, H-5'), 7.40 (1H, ddd, *J* = 8.1, 2.1, 0.9 Hz, H-4' or H-6'), 7.68 (1H, t, *J* = 2.1 Hz, H-2'), 8.22 (1H, br s, NH). The ^{1}H NMR data agreed with the literature values.[4]

2-Chloro-*N*-(3'-bromophenyl)acetamide 14f

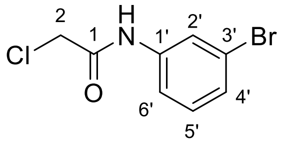

The reaction was carried out following General procedure 3.2.3 using aniline **12f** (0.63 mL, 5.81 mmol), Et_3N (0.81 mL, 5.81 mmol) and chloroacetyl chloride (0.56 mL, 6.98 mmol) in CH_2Cl_2 (28.9 mL) to give the *title compound* **14f** (1.36 g, 95%), as a grey solid. m.p. 114-115 °C. δ_H (400 MHz, $CDCl_3$) 4.19 (2H, s, H-2), 7.22 (1H, t, *J* = 8.0 Hz, H-5'), 7.31 (1H, ddd, *J* = 8.1, 2.0, 1.0 Hz, H-4' or H-6'), 7.47 (1H, ddd, *J* = 8.1, 2.0, 1.0 Hz, H-4' or H-6'), 7.81 (1H, t, *J* = 2.0 Hz, H-2'), 8.22 (1H, br s, NH). The ^{1}H NMR data agreed with the literature values.[5]

2-Chloro-*N*-(3'-methoxyphenyl)acetamide 14g

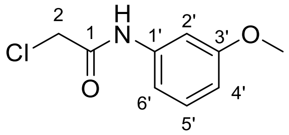

The reaction was carried out following General procedure 3.2.3 using aniline **13g** (0.91 mL, 8.12 mmol), Et_3N (1.13 mL, 8.12 mmol) and chloroacetyl chloride (0.78 mL, 9.74 mmol) in CH_2Cl_2 (40.3 mL) to give the *title compound* **14g** (1.63 g, quant.) as a brown solid. m.p. 94-95 °C (Lit. 93 °C).[4] δ_H (400 MHz, $CDCl_3$) 3.84 (3H, s, 3'-OCH_3), 4.21 (2H, s, H-2), 6.35 (1H, dd, J = 8.2, 2.3 Hz, H-4' or H-6'), 7.06 (1H, ddd, J = 8.2, 2.3, 0.8 Hz, H-4' or H-6'), 7.28 (1H, t, J = 8.2 Hz, H-5'), 7.31 (1H, t, J = 2.3 Hz, H-2'), 8.23 (1H, br s, NH). The ^{1}H NMR data agreed with the literature values.[4]

2-Chloro-*N*-(3'-chloro-2'-methylphenyl)acetamide 14h

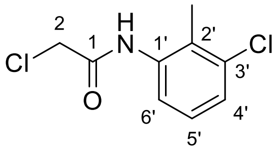

The reaction was carried out following General procedure 3.2.3 using aniline **13h** (0.84 mL, 7.06 mmol), Et_3N (0.98 mL, 7.06 mmol) and chloroacetyl chloride (0.68 mL, 8.48 mmol) in CH_2Cl_2 (35.1 mL) to give the *title compound* **14h** (1.42 g, 92%) as a dark grey solid. m.p. 133-135 °C. δ_H (400 MHz, $CDCl_3$) 2.35 (3H, s, 2'-CH_3), 4.24 (2H, s, H-2), 7.17 (1H, t, J = 8.0 Hz, H-5'), 7.26 (1H, d, J = 8.0 Hz, H-4'), 7.34 (1H, d, J = 8.0 Hz, H-6'), 8.25 (1H, br s, NH). δ_C (100 MHz, $CDCl_3$) 14.5 (2'-CH_3), 43.2 (C-2), 121.8 (C-6'), 127.1 (C-4'), 127.3 (C-5'), 128.3 (C-2'), 135.2 (C-1'), 135.9 (C-3'), 164.1 (C-1). ν_{max} (ATR)/cm^{-1} 3265 (N-H amide), 2955 (C-H alkane), 1665 (C=O amide), 1579 (C=C aromatic), 1438 (-C-H bending), 1264 (C-N aromatic), 819 (C-Cl). *m/z* (ESI^+): 242 ($^{35}ClMNa^+$, 62%), 240 ($^{35}ClMNa^+$, 100%). HRMS (ESI^+) found ($^{37}ClMNa^+$): 241.9924 $C_9H_9{}^{37}Cl_2NNaO$ requires 241.9925. Found ($^{35}ClMNa^+$): 239.9953 $C_9H_9{}^{35}Cl_2NNaO$ requires 239.9953.

2-Chloro-*N*-(3'-bromo-2'-methylphenyl)acetamide 14i

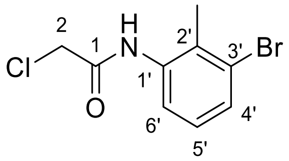

The reaction was carried out following General procedure 3.2.3 using aniline **13i** (0.66 mL, 5.37 mmol), Et_3N (0.75 mL, 5.37 mmol) and chloroacetyl chloride (0.51 mL, 6.45 mmol) in CH_2Cl_2 (26.7 mL) to give the *title compound* **14i** (1.31 g, 93%) as a white solid. m.p. 140-142 °C. δ_H (400 MHz, $CDCl_3$) 2.39 (3H, s, 2'-CH_3), 4.24 (2H, s, H-2), 7.10 (1H, t, *J* = 8.0 Hz, H-5'), 7.45 (1H, d, *J* = 8.0 Hz, H-4'), 7.76 (1H, d, *J* = 8.0 Hz, H-6'), 8.25 (1H, br s, NH). δ_C (100 MHz, $CDCl_3$) 17.7 (2'-CH_3), 43.2 (C-2), 122.6 (C-6'), 125.7 (C-3'), 127.7 (C-5'), 130.1 (C-2'), 130.5 (C-4'), 135.7 (C-1'), 164.1 (C-1). ν_{max} (ATR)/cm^{-1} 3261 (N-H amide), 2959 (C-H alkane), 1665 (C=O amide), 1576 (C=C aromatic), 1436 (-C-H bending), 1264 (C-N aromatic), 693 (C-Br). *m/z* (ESI^+): 286 ($^{81}Br^{37}ClMNa^+$, 100%), 285 ($^{81}Br^{35}ClMNa^+$ or $^{79}Br^{37}ClMNa^+$, 10%), 284 ($^{79}Br^{35}ClMNa^+$, 78%). HRMS (ESI^+) found ($^{81}Br^{37}ClMNa^+$): 285.9424 $C_9H_9{}^{81}Br\ {}^{37}ClNNaO$ requires 285.9426. Found ($^{81}Br^{35}ClMNa^+$ or $^{79}Br^{37}ClMNa^+$): 284.9478 $C_9H_9{}^{81}Br^{35}ClNNaO$ or $C_9H_9{}^{79}Br^{37}ClNNaO$ requires 284.9480. Found ($^{79}Br^{35}ClMNa^+$): 283.9443 $C_9H_9{}^{79}Br^{35}ClNNaO$ requires 283.9448.

2-Chloro-*N*-(naphthalen-1-yl)acetamide 14j

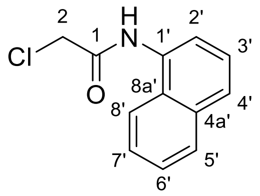

The reaction was carried out following General procedure 3.2.3 using aniline **13j** (0.45 mL, 3.49 mmol), Et_3N (0.49 mL, 3.49 mmol) and chloroacetyl chloride (0.33 mL, 4.19 mmol) in CH_2Cl_2 (26.7 mL) to give the *title compound* **14j** (0.70 g, 91%) as a white solid. m.p. 159-161 °C. (Lit. 163 °C).[6] δ_H (400 MHz, $CDCl_3$) 4.38 (2H, s, H-2), 7.48-7.60 (3H, m, 3 x Ar-H), 7.76 (1H, d, *J* = 8.0 Hz, Ar-H), 7.89 (2H, m, 2 x Ar-H), 7.99 (1H, d, *J* = 8.0 Hz, Ar-H) and 8.78 (1H, br s, NH). The ^1H NMR data is in accordance with literature values.[6]

3-Amino-5-benzoyl-6-methyl-*N*-phenylthieno[2,3-*b*]pyridine-2-carboxamide 4a

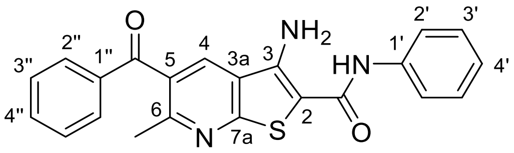

The reaction was carried out following General procedure 3.2.4 using carbonitrile **12a** (100 mg, 0.39 mmol), chloride **14a** (66 mg, 0.39 mmol) and sodium carbonate (56 mg, 0.53 mmol) in EtOH (3 mL) to give the *title compound* **4a** (99 mg, 66%) as a yellow solid. m.p. >230 °C. δ_H (400 MHz, $(CD_3)_2SO$) 2.58 (3H, s, 6-CH_3), 7.08 (1H, t, *J* = 7.8 Hz, H-4'), 7.32 (2H, t, *J* = 7.8 Hz, H-3'), 7.42 (2H, br s, NH_2), 7.60 (2H, t, *J* = 7.9 Hz, H-3"), 7.70 (2H, dd, *J* = 7.8, 0.7 Hz, H-2'), 7.75 (1H, t, *J* = 7.9 Hz, H-4"), 7.82 (2H, dd, *J* = 7.9, 1.2 Hz, H-2"), 8.56 (1H, s, H-4), 9.50 (1H, br s, NH). δ_C (100 MHz, $(CD_3)_2SO$) 23.4 (6-CH_3), 96.5 (C-2), 121.1 (C-2'), 123.2 (C-4'), 123.4 (C-3a), 128.4 (C-3'), 129.0 (C-3"), 129.6 (C-5), 130.0 (C-2"),131.3 (C-4), 133.9 (C-4"), 136.9 (C-1"), 138.9 (C-1'), 147.0 (C-3), 157.2 (C-6), 159.5 (C-7a), 163.8 (CONH), 196.2 (5-CO). ν_{max} (ATR)/cm^{-1} 3423 (N-H amide), 3313 (N-H amine), 2954 (C-H alkane), 1661 (C=O amide), 1638 (C=O carbonyl), 1585 (C=C aromatic), 1436 (-C-H bending), 1254 (C-N aromatic). *m/z* (ESI^+): 410 (MNa^+, 100%), 101 (13%). HRMS (ESI^+) found (MNa^+): 410.0925 $C_{22}H_{17}N_3NaO_2S$ requires 410.0934.

3-Amino-5-benzoyl-6-methyl-*N*-(4'-methoxyphenyl)-6-methylthieno[2,3-*b*]pyridine-2-carboxamide 4b

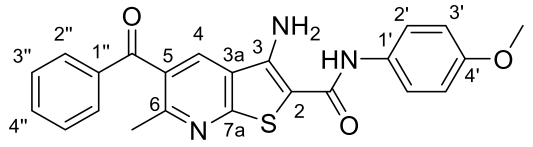

The reaction was carried out following General procedure 3.2.4 using carbonitrile **12a** (100 mg, 0.39 mmol), chloride **14b** (78 mg, 0.39 mmol) and sodium carbonate (56 mg, 0.53 mmol) in EtOH (3 mL) to give the *title compound* **4b** (58 mg, 36%) as a mustard solid. m.p. 192-195 °C. δ_H (400 MHz, $(CD_3)_2SO$) 2.57 (3H, s, 6-CH_3), 3.74 (3H, s, 4'-OCH_3), 6.90 (2H, dt, *J* = 9.0, 2.7 Hz, H-3'), 7.36 (2H, br s, NH_2), 7.57-7.62 (4H, m, H-2' and H-3''), 7.74 (1H, t, *J* = 8.0 Hz, H-4''), 7.82 (2H, dd, *J* = 8.0, 1.1 Hz, H-2''), 8.54 (1H, s, H-4), 9.38 (1H, br s, NH). δ_C (100 MHz, $(CD_3)_2SO$) 23.4 (6-CH_3), 55.2 (4'-CH_3), 113.5 (C-3'), 122.9 (C-2'), 123.3 (C-3a), 129.0 (C-3''), 129.6 (C-5), 130.0 (C-2''),131.2 (C-4), 132.0 (C-1'), 133.9 (C-4''), 136.8 (C-1''), 146.5 (C-3), 155.5 (C-4'), 157.0 (C-6), 159.4 (C-7a), 163.5 (CONH), 196.3 (5-CO). C-2 not observed. ν_{max} (ATR)/cm^{-1} 3425 (N-H amide), 3321 (N-H amine), 2939 (C-H alkane), 1661 (C=O amide), 1632 (C=O carbonyl), 1595 (C=C aromatic), 1451 (-C-H bending), 1246 (C-N aromatic), 1182 (C-O ether). *m/z* (ESI^+): 440 (MNa^+, 100%). HRMS (ESI^+) found (MNa^+): 440.1035 $C_{23}H_{19}N_3NaO_3S$ requires 440.1039.

3-Amino-5-benzoyl-*N*-(4'-chlorophenyl)-6-methylthieno[2,3-*b*]pyridine-2-carboxamide 4c

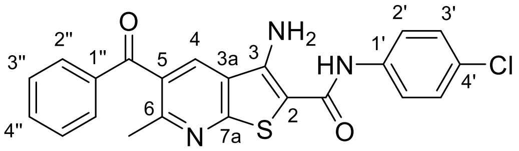

The reaction was carried out following General procedure 3.2.4 using carbonitrile **12a** (100 mg, 0.39 mmol), chloride **14c** (79 mg, 0.39 mmol) and sodium carbonate (56 mg, 0.53 mmol) in EtOH (3 mL) to give the *title compound* **4c** (64 mg, 40%) as a yellow solid. m.p. >230 °C. δ_H (400 MHz, $(CD_3)_2SO$) 2.57 (3H, s, 6-CH_3), 7.36 (2H, dt, *J* = 8.9, 2.3 Hz, H-3'), 7.44 (2H, br s, NH_2), 7.60 (2H, t, *J* = 8.1 Hz, H-3''), 7.73-7.76 (3H, m, H-2' and H-4''), 7.81 (2H, dd, *J* = 8.1, 1.1 Hz, H-2''), 8.56 (1H, s, H-4), 9.62 (1H, br s, NH). δ_C (100 MHz, $(CD_3)_2SO$) 23.4 (6-CH_3), 122.6 (C-2'), 123.2 (C-3a), 127.0 (C-4'), 128.3 (C-3'), 129.0 (C-3''), 129.7 (C-5), 130.0 (C-2''), 131.4 (C-4), 134.0 (C-4''), 136.8 (C-1''), 138.1 (C-1'), 147.3 (C-3), 157.3 (C-6), 159.6 (C-7a), 163.8 (CONH), 196.3 (5-CO). C-2 not observed. ν_{max} (ATR)/cm^{-1} 3433 (N-H amide), 3322 (N-H amine), 1660 (C=O amide), 1634 (C=O carbonyl), 1584 (C=C aromatic), 1440 (-C-H bending), 1242 (C-N aromatic), 805 (C-Cl). *m/z* (ESI^+): 446 ($^{37}ClMNa^+$, 37%), 444 ($^{35}ClMNa^+$, 100%), 217 (20%), 159 (21%), 101 (65%). HRMS (ESI^+) found ($^{37}ClMNa^+$): 446.0516 $C_{22}H_{16}{}^{37}ClN_3NaO_2S$ requires 446.0521. Found ($^{35}ClMNa^+$): 444.0546 $C_{22}H_{16}{}^{35}ClN_3NaO_2S$ requires 444.0544.

3-Amino-5-benzoyl-*N*-(4'-bromophenyl)-6-methylthieno[2,3-*b*]pyridine-2-carboxamide 4d

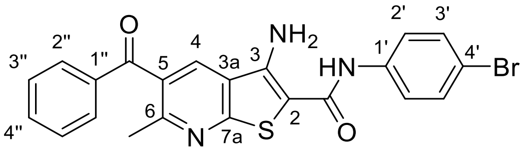

The reaction was carried out following General procedure 3.2.4 using carbonitrile **12a** (100 mg, 0.39 mmol), chloride **14d** (96 mg, 0.39 mmol) and sodium carbonate (56 mg, 0.53 mmol) in EtOH (3 mL) to give the *title compound* **4d** (70 mg, 39%) as a mustard solid. m.p. decomp. at 205 °C. δ_H (400 MHz, $(CD_3)_2SO$) 2.57 (3H, s, 6-CH_3), 7.45 (2H, br s, NH_2), 7.50 (2H, dt, J = 8.8, 2.4 Hz, H-3'), 7.60 (2H, t, J = 7.8 Hz, H-3''), 7.70 (2H, dt, J = 8.8, 2.4 Hz, H-2'), 7.75 (1H, d, J = 7.8 Hz, H-4''), 7.82 (2H, dd, J = 7.8, 1.1 Hz, H-2''), 8.57 (1H, s, H-4), 9.62 (1H, br s, NH). δ_C (100 MHz, $(CD_3)_2SO$) 23.4 (6-CH_3), 92.9 (C-2), 115.1 (C-4'), 122.9 (C-2'), 123.1 (C-3a), 129.0 (C-3''), 129.6 (C-5), 130.0 (C-2''), 131.2 (C-3'), 131.4 (C-4), 134.0 (C-4''), 136.8 (C-1''), 138.4 (C-1'), 147.4 (C-3), 155.4 (C-6), 159.6 (C-7a), 163.8 (CONH), 196.2 (5-CO). ν_{max} (ATR)/cm^{-1} 3405 (N-H amide), 3302 (N-H amine), 2923 (C-H alkane), 1665 (C=O amide), 1647 (C=O carbonyl), 1581 (C=C aromatic), 1482 (-C-H bending), 1251 (C-N aromatic), 687 (C-Br). *m/z* (ESI^+): 490 ($^{79}BrMNa^+$, 100%), 488 ($^{79}BrMNa^+$, 100%), 468 (31%), 466 (31%), 398 (10%), 379 (12%), 351 (12%), 227 (31%), 217 (36%), 179 (15%), 159 (35%), 101 (66%). HRMS (ESI^+) found ($^{81}BrMNa^+$): 490.0005 $C_{22}H_{16}{}^{81}BrN_3NaO_3S$ requires 490.0020. Found ($^{79}BrMNa^+$): 488.0031 $C_{22}H_{16}{}^{79}BrN_3NaO_3S$ requires 488.0039.

3-Amino-5-benzoyl-*N*-(3'-chlorophenyl)-6-methylthieno[2,3-*b*]pyridine-2-carboxamide 4e

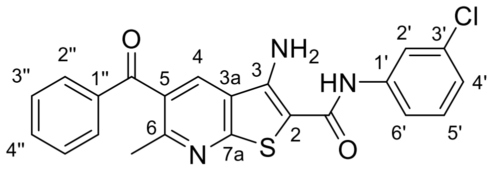

The reaction was carried out following General procedure 3.2.4 using carbonitrile **12a** (100 mg, 0.39 mmol), chloride **14e** (79 mg, 0.39 mmol) and sodium carbonate (56 mg, 0.53 mmol) in EtOH (3 mL) to give the *title compound* **4e** (47 mg, 30%) as a yellow solid. m.p. >230 °C. δ_H (400 MHz, $(CD_3)_2SO$) 2.57 (3H, s, 6-CH_3), 7.12 (1H, dd, *J* = 7.9, 1.9 Hz, H-4'), 7.35 (1H, t, *J* = 7.9 Hz, H-5'), 7.49 (2H, br s, NH_2), 7.60 (2H, t, *J* = 7.7 Hz, H-3''), 7.65 (1H, d, *J* = 7.9 Hz, H-6'), 7.75 (1H, t, *J* = 7.7 Hz, H-4''), 7.82 (2H, d, *J* = 7.7 Hz, H-2''), 7.92 (1H, t, *J* = 1.9 Hz, H-2'), 8.58 (1H, s, H-4), 9.65 (1H, br s, NH). δ_C (100 MHz, $(CD_3)_2SO$) 23.4 (6-CH_3), 95.8 (C-2), 119.1 (C-6'), 120.2 (C-2'), 123.0 (C-3a), 123.0 (C-4'), 129.0 (C-3''), 129.6 (C-5), 130.0 (C-2''), 130.1 (C-5'), 131.5 (C-4), 132.7 (C-3'), 133.9 (C-4''), 136.8 (C-1''), 140.6 (C-1'), 147.6 (C-3), 157.5 (C-6), 159.6 (C-7a), 163.9 (CONH), 196.2 (5-CO). v_{max} (ATR)/cm^{-1} 3457 (N-H amide), 3338 (N-H amine), 2972 (C-H alkane), 1667 (C=O amide), 1649 (C=O carbonyl), 1581 (C=C aromatic), 1448 (-C-H bending), 1241 (C-N aromatic), 809 (C-Cl). *m/z* (ESI^+): 446 ($^{37}ClMNa^+$, 34%), 444 ($^{35}ClMNa^+$, 100%), 227 (27%), 159 (26%), 101 (42%). HRMS (ESI^+) found ($^{37}ClMNa^+$): 446.0516 $C_{22}H_{16}{}^{37}ClN_3NaO_2S$ requires 446.0521. Found ($^{35}ClMNa^+$): 444.0543 $C_{22}H_{16}{}^{35}ClN_3NaO_2S$ requires 444.0544.

3-Amino-5-benzoyl-*N*-(3'-bromophenyl)-6-methylthieno[2,3-*b*]pyridine-2-carboxamide 4f

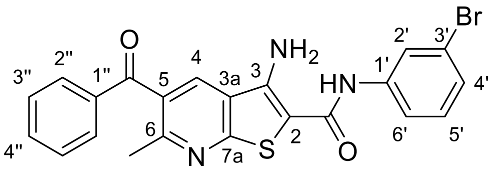

The reaction was carried out following General procedure 3.2.4 using carbonitrile **12a** (100 mg, 0.39 mmol), chloride **14f** (96 mg, 0.39 mmol) and sodium carbonate (56 mg, 0.53 mmol) in EtOH (3 mL) to give the *title compound* **4f** (76 mg, 42%) as a yellow solid. m.p. >230 °C. δ_H (400 MHz, $(CD_3)_2SO$) 2.57 (3H, s, 6-CH_3), 7.26-7.30 (2H, m, H-4' and H-5'), 7.49 (2H, br s, NH_2), 7.60 (2H, t, *J* = 7.8 Hz, H-3''), 7.65 (1H, dt, *J* = 7.8, 1.8 Hz, H-6'), 7.74 (1H, t, *J* = 7.8 Hz, H-4''), 7.81 (2H, d, *J* = 7.8 Hz, H-2''), 8.07 (1H, t, *J* = 1.8 Hz, H-2'), 8.58 (1H, s, H-4), 9.63 (1H, br s, NH). δ_C (100 MHz, $(CD_3)_2SO$) 23.4 (6-CH_3), 95.8 (C-2), 119.5 (C-6'), 121.2 (C-3'), 123.0 (C-2'), 123.1 (C-3a), 125.8 (C-4'), 129.0 (C-3''), 129.6 (C-5), 130.0 (C-2''), 130.4 (C-5'), 131.4 (C-4), 133.9 (C-4''), 136.8 (C-1''), 140.7 (C-1'), 147.6 (C-3), 157.5 (C-6), 159.6 (C-7a), 163.8 (CONH), 196.2 (5-CO). ν_{max} (ATR)/cm^{-1} 3457 (N-H amide), 3338 (N-H amine), 2970 (C-H alkane), 1667 (C=O amide), 1648 (C=O carbonyl), 1581 (C=C aromatic), 1478 (-C-H bending), 1251 (C-N aromatic), 672 (C-Br). *m/z* (ESI^+): 490 ($^{79}BrMNa^+$, 100%), 488 ($^{79}BrMNa^+$, 94%), 468 (13%), 466 (12%), 379 (20%), 351 (18%), 295 (10%), 227 (51%), 217 (22%), 179 (12%), 159 (50%), 101 (85%). HRMS (ESI^+) found ($^{81}BrMNa^+$): 490.0002 $C_{22}H_{16}{}^{81}BrN_3NaO_3S$ requires 490.0020. Found ($^{79}BrMNa^+$): 488.0022 $C_{22}H_{16}{}^{79}BrN_3NaO_3S$ requires 488.0039.

3-Amino-5-benzoyl-*N*-(3'-methoxyphenyl)-6-methylthieno[2,3-*b*]pyridine-2-carboxamide 4g

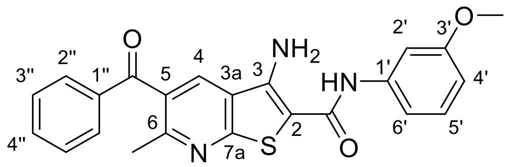

The reaction was carried out following General procedure 3.2.4 using carbonitrile **12a** (100 mg, 0.39 mmol), chloride **14g** (78 mg, 0.39 mmol) and sodium carbonate (56 mg, 0.53 mmol) in EtOH (3 mL) to give the *title compound* **4g** (47 mg, 29%) as a yellow solid. m.p. 200-202 °C. δ_H (400 MHz, $(CD_3)_2SO$) 2.57 (3H, s, 6-CH_3), 3.75 (3H, s, 3'-OCH_3), 6.64 (1H, dd, *J* = 8.2, 2.1 Hz, H-4'), 7.21 (1H, t, *J* = 8.2 Hz, H-5'), 7.33 (1H, d, *J* = 8.2 Hz, H-6'), 7.39 (3H, m, NH_2 and H-2'), 7.60 (2H, t, *J* = 7.8 Hz, H-3''), 7.74 (1H, t, *J* = 7.8 Hz, H-4''), 7.82 (2H, dd, *J* = 7.8, 1.1 Hz, H-2''), 8.56 (1H, s, H-4), 9.44 (1H, br s, NH). δ_C (100 MHz, $(CD_3)_2SO$) 23.4 (6-CH_3), 54.9 (3'-OCH_3), 106.5 (C-2'), 108.9 (C-4'), 113.1 (C-6'), 123.1 (C-3a), 129.0 (C-3''), 129.1 (C-5'), 129.5 (C-5), 130.0 (C-2''), 131.3 (C-4), 133.9 (C-4''), 136.8 (C-1''), 140.4 (C-1'), 147.0 (C-3), 157.2 (C-6), 159.3 (C-7a), 159.4 (C-3'), 163.7 (CONH), 196.2 (5-CO). C-2 not observed. ν_{max} (ATR)/cm^{-1} 3393 (N-H amide), 3309 (N-H amine), 2960 (C-H alkane), 1656 (C=O carbonyl), 1587 (C=C aromatic), 1487 (-C-H bending), 1254 (C-N aromatic), 1165 (C-O ether). *m/z* (ESI$^+$): 440 (MNa^+, 100%). HRMS (ESI$^+$) found (MNa^+): 440.1026 $C_{23}H_{19}N_3NaO_3S$ requires 440.1039.

3-Amino-5-benzoyl-*N*-(3'-chloro-2'-methylphenyl)-6-methylthieno[2,3-*b*]pyridine-2-carboxamide 4h

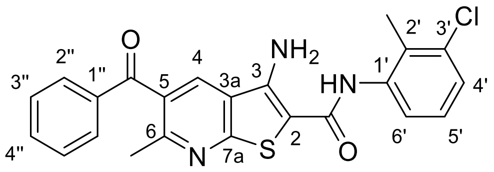

The reaction was carried out following General procedure 3.2.4 using carbonitrile **12a** (100 mg, 0.39 mmol), chloride **14h** (85 mg, 0.39 mmol) and sodium carbonate (56 mg, 0.53 mmol) in EtOH (3 mL) to give the *title compound* **4h** (56 mg, 33%) as a brown solid. m.p. >230 °C. δ_H (400 MHz, $(CD_3)_2SO$) 2.24 (3H, s, 2'-CH_3), 2.57 (3H, s, 6-CH_3), 7.20 (1H, t, *J* = 7.9 Hz, H-5'), 7.28-7.34 (4H, m, H-4', H-6', and NH_2), 7.60 (2H, t, *J* = 7.8 Hz, H-3''), 7.74 (1H, t, *J* = 7.8 Hz, H-4''), 7.81 (2H, dd, *J* = 7.8, 1.3 Hz, H-2''), 8.52 (1H, s, H-4), 9.47 (1H, br s, NH). δ_C (100 MHz, $(CD_3)_2SO$) 15.4 (2'-CH_3), 23.4 (6-CH_3), 123.3 (C-3a), 125.8 (C-6'), 126.6 (C-5'), 128.7 (C-4'), 129.0 (C-3''), 129.4 (C-5), 130.0 (C-2''), 131.2 (C-4), 132.2 (C-2'), 133.5 (C-3'), 133.6 (C-1'), 133.9 (C-4''), 136.8 (C-1''), 146.3 (C-3), 156.7 (C-6), 159.5 (C-7a), 164.0 (CONH), 196.3 (5-CO). C-2 not observed. ν_{max} (ATR)/cm^{-1} 3300 (N-H amine and amide), 2973 (C-H alkane), 1659 (C=O carbonyl), 1581 (C=C aromatic), 1483 (-C-H bending), 1252 (C-N aromatic), 805 (C-Cl). *m/z* (ESI^+): 460 ($^{37}ClMNa^+$, 40%), 458 ($^{35}ClMNa^+$, 100%), (10%), 227 (17%), (10%), 159 (15%), 101 (19%). HRMS (ESI^+) found ($^{37}ClMNa^+$): 460.0670 $C_{23}H_{18}{}^{37}ClN_3NaO_2S$ requires 460.0678. Found ($^{35}ClMNa^+$): 458.0699 $C_{23}H_{18}{}^{35}ClN_3NaO_2S$ requires 458.0700.

3-Amino-5-benzoyl-*N*-(3'-bromo-2'-methylphenyl)-6-methylthieno[2,3-*b*]pyridine-2-carboxamide 4i

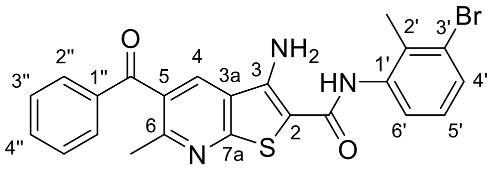

The reaction was carried out following General procedure 3.2.4 using carbonitrile **12a** (100 mg, 0.39 mmol), chloride **14i** (102 mg, 0.39 mmol) and sodium carbonate (56 mg, 0.53 mmol) in EtOH (3 mL) to give the *title compound* **4i** (56 mg, 30%) as a yellow solid. m.p. >230 °C. δ_H (400 MHz, $(CD_3)_2SO$) 2.27 (3H, s, 2'-CH_3), 2.57 (3H, s, 6-CH_3), 7.14 (1H, t, *J* = 7.9 Hz, H-5'), 7.32 (2H, br s, NH_2), 7.35 (1H, d, *J* = 7.9 Hz, H-6'), 7.47 (1H, d, *J* = 7.9 Hz, H-4'), 7.60 (2H, t, *J* = 7.9 Hz, H-3''), 7.75 (1H, t, *J* = 7.9 Hz, H-4''), 7.81 (2H, dd, *J* = 7.9, 1.4 Hz, H-2''), 8.52 (1H, s, H-4), 9.49 (1H, br s, NH). δ_C (100 MHz, $(CD_3)_2SO$) 18.5 (2'-CH_3), 23.4 (6-CH_3), 95.8 (C-2), 123.9 (C-3a), 124.5 (C-3'), 126.6 (C-6'), 127.1 (C-5'), 128.8 (C-4' '), 129.0 (C-3''), 129.5 (C-5), 130.0 (C-2''), 131.2 (C-4), 133.8 (C-2'), 133.9 (C-4''), 136.8 (C-1''), 138.7 (C-1'), 146.6 (C-3), 156.9 (C-6), 159.5 (C-7a), 164.0 (CONH), 196.2 (5-CO). ν_{max} (ATR)/cm^{-1} 3310 (N-H amine and amide), 2972 (C-H alkane), 1656 (C=O carbonyl), 1582 (C=C aromatic), 1484 (-C-H bending), 1253 (C-N aromatic), 687 (C-Br). *m/z* (ESI^+): 490 ($^{79}BrMNa^+$, 100%), 488 ($^{79}BrMNa^+$, 94%), 379 (10%), 227 (26%), 217 (15%), 159 (23%), 101 (34%). HRMS (ESI^+) found ($^{81}BrMNa^+$): 504.0162 $C_{23}H_{18}{}^{81}BrN_3NaO_3S$ requires 504.0177. Found ($^{79}BrMNa^+$): 502.0189 $C_{23}H_{18}{}^{79}BrN_3NaO_3S$ requires 502.0195.

3-Amino-5-benzoyl-6-methyl-*N*-(naphthalen-1'-yl)thieno[2,3-*b*]pyridine-2-carboxamide 4j

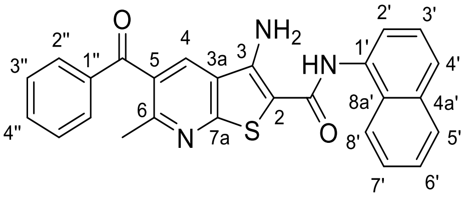

The reaction was carried out following General procedure 3.2.4 using carbonitrile **12a** (100 mg, 0.39 mmol), chloride **14j** (86 mg, 0.39 mmol) and sodium carbonate (56 mg, 0.53 mmol) in EtOH (3 mL) to give the *title compound* **4j** (58 mg, 34%) as a mustard solid. m.p. 200-202 °C. δ_H (400 MHz, $(CD_3)_2SO$) 2.59 (3H, s, 6-CH_3), 7.53-7.57 (4H, m, Ar-H), 7.34 (2H, br s, NH_2), 7.61 (2H, t, *J* = 7.8 Hz, H-3''), 7.75 (1H, t, *J* = 7.8 Hz, H-4''), 7.83 (2H, dd, *J* = 7.8, 1.1 Hz, H-2''), 8.56 (1H, s, H-4), 7.57 (1H, t, *J* = 4.7 Hz, Ar-H), 7.93-7.98 (2H, m, Ar-H), 9.77 (1H, br s, NH). δ_C (100 MHz, $(CD_3)_2SO$) 23.4 (6-CH_3), 97.0 (C-2), 123.2 (Ar-CH), 123.4 (C-3a), 124.35 (Ar-CH), 124.45 (Ar-CH), 125.87 (Ar-CH), 125.94 (Ar-CH), 126.3 (Ar-CH), 128.0 (Ar-CH), 129.0 (C-3''), 129.5 (C-5), 129.7 (Ar-C), 130.0 (C-2''), 131.3 (C-4), 133.7 (C-1' or Ar-CH), 133.8 (C-1' or Ar-CH), 133.9 (C-4''), 136.8 (C-1''), 146.7 (C-3), 157.4 (C-6), 159.5 (C-7a), 164.0 (CONH), 196.2 (5-CO). ν_{max} (ATR)/cm^{-1} 3380 (N-H amide), 3300 (N-H amine), 2980 (C-H alkane), 1664 (C=O carbonyl), 1595 (C=C aromatic), 1480 (-C-H bending), 1254 (C-N aromatic). *m/z* (ESI^+): 460 (MNa^+, 100%). HRMS (ESI^+) found (MNa^+): 460.1078 $C_{26}H_{19}N_3NaO_2S$ requires 460.1090.

3-Amino-5-(4''-methoxybenzoyl)-6-methyl-*N*-phenylthieno[2,3-*b*]pyridine-2-carboxamide 5a

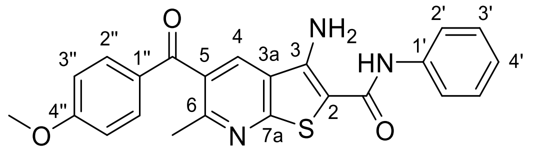

The reaction was carried out following General procedure 3.2.4 using carbonitrile **12b** (100 mg, 0.35 mmol), chloride **14a** (60 mg, 0.35 mmol) and sodium carbonate (74 mg, 0.70 mmol) in EtOH (2.7 mL) to give the *title compound* **5a** (42 mg, 29%) as a mustard solid. m.p. 119-120 °C. δ_H (400 MHz, $(CD_3)_2SO$) 2.54 (3H, s, 6-CH_3), 3.88 (3H, s, 4''-OCH_3), 7.08 (1H, t, *J* = 7.8 Hz, H-4'), 7.12 (2H, d, *J* = 8.8 Hz, H-3''), 7.33 (2H, t, *J* = 7.8 Hz, H-3'), 7.40 (2H, br s, NH_2), 7.70 (2H, d, *J* = 7.8 Hz, H-2'), 7.79 (2H, dt, *J* = 8.8, 2.4 Hz, H-2''), 8.52 (1H, s, H-4), 9.48 (1H, br s, NH). δ_C (100 MHz, $(CD_3)_2SO$) 23.4 (6-CH_3), 55.7 (4''-OCH_3), 114.3 (C-3''), 121.1 (C-2'), 123.2 (C-4'), 123.4 (C-3a), 128.4 (C-3'), 129.5 (C-1''), 130.1 (C-5), 130.7 (C-4), 132.5 (C-2''), 138.9 (C-1'), 147.0 (C-3), 159.1 (C-7a), 163.8 (C-4''), 163.9 (CONH). C-2, C-6 and 5-CO not observed. ν_{max} (ATR)/cm^{-1} 3418 (N-H amide), 3318 (N-H amine), 2954 (C-H alkane), 1706 (C=O amide), 1656 (C=O carbonyl), 1587 (C=C aromatic), 1437 (-C-H bending), 1249 (C-N aromatic), 1177 (C-O ether). *m/z* (ESI$^+$): 440 (MNa$^+$, 100%). HRMS (ESI$^+$) found (MNa$^+$): 440.1028 $C_{23}H_{19}N_3NaO_2S$ requires 440.1039.

3-Amino-5-(4''-methoxybenzoyl)-*N*-(4'-methoxyphenyl)-6-methylthieno[2,3-*b*]pyridine-2-carboxamide 5b

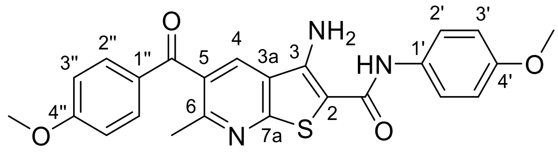

The reaction was carried out following General procedure 3.2.4 using carbonitrile **12b** (100 mg, 0.35 mmol), chloride **14b** (70 mg, 0.35 mmol) and sodium carbonate (74 mg, 0.70 mmol) in EtOH (2.70 mL) to give the *title compound* **5b** (62 mg, 40%) as a brown solid. m.p. 115-117 °C. δ_H (400 MHz, $(CD_3)_2SO$) 2.53 (3H, s, 6-CH_3), 3.74 (3H, s, 4'-OCH_3), 3.87 (3H, s, 4''-OCH_3), 6.90 (2H, dt, J = 9.0, 2.7 Hz, H-3'), 7.11 (2H, dt, J = 8.9, 2.5 Hz, H-3''), 7.34 (2H, br s, NH_2), 7.58 (2H, dt, J = 9.0, 2.7 Hz, H-2'), 7.79 (2H, dt, J = 8.9, 2.2 Hz, H-2''), 8.50 (1H, s, H-4), 9.38 (1H, br s, NH). δ_C (100 MHz, $(CD_3)_2SO$) 23.2 (6-CH_3), 55.2 (4'-CH_3), 55.7 (4''-OCH_3), 96.9 (C-2), 113.5 (C-3'), 114.3 (C-3''), 122.9 (C-2'), 123.2 (C-3a), 129.5 (C-1''), 130.6 (C-5), 130.1 (C-4), 131.8 (C-1'), 132.5 (C-2''), 146.6 (C-3), 155.5 (C-4'), 156.6 (C-6), 159.1 (C-7a), 163.6 (C-4''), 163.8 (CONH), 194.6 (5-CO). ν_{max} (ATR)/cm^{-1} 3444 (N-H amide), 3336 (N-H amine), 2931 (C-H alkane), 1658 (C=O amide), 1638 (C=O carbonyl), 1582 (C=C aromatic), 1445 (-C-H bending), 1255 (C-N aromatic), 1184 (C-O ether), 1169 (C-O ether). *m/z* (ESI^+): 470 (MNa^+, 100%), 227 (10%). HRMS (ESI^+) found (MNa^+): 470.1143 $C_{24}H_{21}N_3NaO_4S$ requires 470.1145.

3-Amino-5-(4''-methoxybenzoyl)-*N*-(4'-chlorophenyl)-6-methylthieno[2,3-*b*]pyridine-2-carboxamide 5c

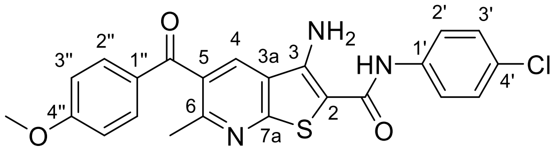

The reaction was carried out following General procedure 3.2.4 using carbonitrile **12b** (100 mg, 0.35 mmol), chloride **14c** (71 mg, 0.35 mmol) and sodium carbonate (56 mg, 0.53 mmol) in EtOH (2.7 mL) to give the *title compound* **5c** (54 mg, 34%) as a mustard solid. m.p. >230 °C. δ_H (400 MHz, $(CD_3)_2SO$) 2.54 (3H, s, 6-CH_3), 3.87 (3H, s, 4''-OCH_3), 7.11 (2H, dt, *J* = 8.9, 2.4 Hz, H-3''), 7.44 (2H, br s, NH_2), 7.38 (1H, dt, *J* = 8.9, 2.4 Hz, H-3'), 7.50 (1H, dt, *J* = 8.9, 2.4 Hz, H-3'), 7.70 (1H, dt, *J* = 8.9, 2.4 Hz, H-2'), 7.75 (1H, dt, *J* = 8.9, 2.4 Hz, H-2'), 7.79 (2H, dt, *J* = 8.9, 2.3 Hz, H-2''), 8.52 (1H, s, H-4), 9.60 (1H, br s, NH). δ_C (100 MHz, $(CD_3)_2SO$) 23.2 (6-CH_3), 55.7 (4''-OCH_3), 96.0 (C-2), 114.3 (C-3''), 122.5 (C-2'), 123.8 (C-3a), 126.9 (C-4'), 128.3 (C-3'), 129.5 (C-1''), 130.3 (C-5), 130.7 (C-4), 132.5 (C-2''), 137.8 (C-1'), 147.3 (C-3), 157.3 (C-6), 159.1 (C-7a), 163.6 (C-4''), 163.8 (CONH), 194.6 (5-CO). ν_{max} (ATR)/cm^{-1} 3446 (N-H amide), 3340 (N-H amine), 1659 (C=O amide), 1638 (C=O carbonyl), 1582 (C=C aromatic), 1483 (-C-H bending), 1258 (C-N aromatic), 1169 (C-O ether), 806 (C-Cl). *m/z* (ESI^+): 476 ($^{37}ClMNa^+$, 41%), 474 ($^{35}ClMNa^+$, 100%), 452 (29%), 431 (61%), 421 (28%), 413 (21%), 398 (20%), 381 (95%), 363 (69%), 353 (21%). HRMS (ESI^+) found ($^{37}ClMNa^+$): 476.0611 $C_{23}H_{18}{}^{37}ClN_3NaO_2S$ requires 476.0627. Found ($^{35}ClMNa^+$): 474.0638 $C_{23}H_{18}{}^{35}ClN_3NaO_2S$ requires 474.0650.

3-Amino-5-(4''-methoxybenzoyl)-*N*-(4'-bromophenyl)-6-methylthieno[2,3-*b*]pyridine-2-carboxamide 5d

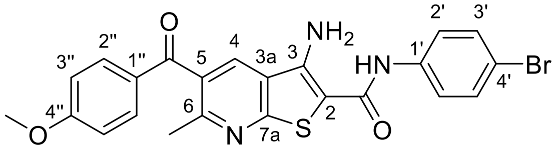

The reaction was carried out following General procedure 3.2.4 using carbonitrile **12b** (100 mg, 0.35 mmol), chloride **14d** (86 mg, 0.35 mmol) and sodium carbonate (74 mg, 0.70 mmol) in EtOH (2.7 mL) to give the *title compound* **5d** (59 mg, 34%) as a yellow solid. m.p. >230 °C. δ_H (400 MHz, $(CD_3)_2SO$) 2.54 (3H, s, 6-CH_3), 3.87 (3H, s, 4''-OCH_3), 7.11 (2H, dt, *J* = 8.9, 2.2 Hz, H-3''), 7.44 (2H, br s, NH_2), 7.50 (2H, dt, *J* = 8.9, 2.4 Hz, H-3'), 7.70 (2H, dt, *J* = 8.9, 2.5 Hz, H-2'), 7.79 (2H, dt, *J* = 8.9, 2.4 Hz, H-2''), 8.53 (1H, s, H-4), 9.61 (1H, br s, NH). δ_C (100 MHz, $(CD_3)_2SO$) 23.2 (6-CH_3), 55.7 (4''-OCH_3), 96.0 (C-2), 114.3 (C-3''), 115.1 (C-4'), 122.8 (C-2'), 123.1 (C-3a), 129.5 (C-1''), 130.2 (C-5), 130.8 (C-4), 131.2 (C-3'), 132.5 (C-2''), 138.4 (C-1'), 147.3 (C-3), 157.0 (C-6), 159.2 (C-7a), 163.8 (C-4''), 163.9 (CONH), 194.6 (5-CO). ν_{max} (ATR)/cm^{-1} 3444 (N-H amide), 3336 (N-H amine), 2930 (C-H alkane), 1658 (C=O amide), 1639 (C=O carbonyl), 1582 (C=C aromatic), 1483 (-C-H bending), 1253 (C-N aromatic), 1169 (C-O ether), 680 (C-Br). *m/z* (ESI^+): 520 ($^{81}BrMNa^+$, 100%), 518 ($^{79}BrMNa^+$, 97%), 498, (33%), 496 (34%), 431 (18%), 381 (53%), 295 (18%), 227 (87%), 159 (54%), 101 (47%). HRMS (ESI^+) found ($^{81}BrMNa^+$): 520.0121 $C_{23}H_{18}{}^{81}BrN_3NaO_3S$ requires 520.0126. Found ($^{79}BrMNa^+$): 518.0139 $C_{23}H_{18}{}^{79}BrN_3NaO_3S$ requires 518.0144.

3-Amino-5-(4''-methoxybenzoyl)-*N*-(3'-chlorophenyl)-6-methylthieno[2,3-*b*]pyridine-2-carboxamide 5e

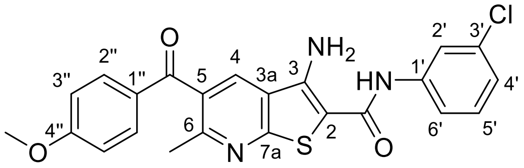

The reaction was carried out following General procedure 3.2.4 using carbonitrile **12b** (100 mg, 0.35 mmol), chloride **14e** (71 mg, 0.35 mmol) and sodium carbonate (56 mg, 0.53 mmol) in EtOH (2.7 mL) to give the *title compound* **5e** (39 mg, 25%) as a mustard solid. m.p. >230 °C. δ_H (400 MHz, $(CD_3)_2SO$) 2.54 (3H, s, 6-CH_3), 3.88 (3H, s, 4''-OCH_3), 7.11 (3H, m, H-3'' and H-4'), 7.34 (1H, t, *J* = 8.2 Hz, H-5'), 7.46 (2H, br s, NH_2), 7.64 (1H, d, *J* = 8.2 Hz, H-6'), 7.79 (2H, dt, *J* = 8.9, 2.3 Hz, H-2''), 7.92 (1H, t, *J* = 2.0 Hz, H-2'), 8.53 (1H, s, H-4), 9.64 (1H, br s, NH). δ_C (100 MHz, $(CD_3)_2SO$) 23.2 (6-CH_3), 55.7 (4''-OCH_3), 95.8 (C-2), 114.3 (C-3''), 119.2 (C-6'), 120.3 (C-2'), 123.2 (C-3a), 122.6 (C-4'), 129.4 (C-1''), 129.5 (C-5'), 130.4 (C-5), 130.8 (C-4), 132.5 (C-2''), 132.6 (C-3'), 140.7 (C-1'), 147.5 (C-3), 157.7 (C-6), 159.2 (C-7a), 163.9 (CONH), 164.1 (C-4''), 194.6 (5-CO). ν_{max} (ATR)/cm^{-1} 3438 (N-H amide), 3317 (N-H amine), 2973 (C-H alkane), 1658 (C=O amide), 1639 (C=O carbonyl), 1582 (C=C aromatic), 1460 (-C-H bending), 1255 (C-N aromatic), 1169 (C-O ether), 803 (C-Cl). *m/z* (ESI^+): 476 ($^{37}ClMNa^+$, 37%), 474 ($^{35}ClMNa^+$, 100%), 452 (25%), 381 (27%), 295 (13%), 227 (62%), 159 (41%), 101 (37%). HRMS (ESI^+) found ($^{37}ClMNa^+$): 476.0629 $C_{23}H_{18}{}^{37}ClN_3NaO_3S$ requires 476.0627. Found ($^{35}ClMNa^+$): 474.0648 $C_{23}H_{18}{}^{35}ClN_3NaO_3S$ requires 474.0650.

3-Amino-5-(4''-methoxybenzoyl)-*N*-(3'-bromophenyl)-6-methylthieno[2,3-*b*]pyridine-2-carboxamide 5f

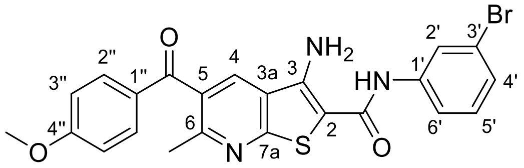

The reaction was carried out following General procedure 3.2.4 using carbonitrile **12b** (100 mg, 0.35 mmol), chloride **14f** (86 mg, 0.35 mmol) and sodium carbonate (74 mg, 0.70 mmol) in EtOH (2.7 mL) to give the *title compound* **5f** (34 mg, 20%) as a mustard solid. m.p. >230 °C. δ_H (400 MHz, $(CD_3)_2SO$) 2.54 (3H, s, 6-CH_3), 3.88 (3H, s, 4''-OCH_3), 7.11 (2H, dt, *J* = 8.8, 2.4 Hz, H-3''), 7.23 (1H, d, *J* = 8.0 Hz, H-4'), 7.28 (1H, t, *J* = 8.0 Hz, H-5'), 7.46 (2H, br s, NH_2), 7.68 (1H, d, *J* = 8.0 Hz, H-6'), 7.79 (2H, dt, *J* = 8.8, 2.4 Hz, H-2''), 8.07 (1H, t, *J* = 1.8 Hz, H-2'), 8.53 (1H, s, H-4), 9.63 (1H, br s, NH). δ_C (100 MHz, $(CD_3)_2SO$) 23.2 (6-CH_3), 55.7 (4''-OCH_3), 92.5 (C-2), 114.3 (C-3''), 119.6 (C-6'), 121.2 (C-3'), 122.8 (C-2'), 123.2 (C-3a), 125.2 (C-4'), 129.5 (C-1''), 129.9 (C-5'), 130.3 (C-4), 130.7 (C-5), 132.5 (C-2''), 141.8 (C-1'), 147.2 (C-3), 156.9 (C-6), 159.2 (C-7a), 163.8 (C-4''), 163.9 (CONH), 194.7 (5-CO). ν_{max} (ATR)/cm^{-1} 3407 (N-H amide), 3297 (N-H amine), 2973 (C-H alkane), 1659 (C=O amide and C=O carbonyl), 1583 (C=C aromatic), 1489 (-C-H bending), 1251 (C-N aromatic), 1171 (C-O ether), 692 (C-Br). *m/z* (ESI^+): 520 ($^{81}BrMNa^+$, 100%), 518 ($^{79}BrMNa^+$, 95%), 498 (23%), 496 (22%), 431 (13%), 381 (73%), 295 (13%), 227 (68%), 159 (44%), 101 (49%). HRMS (ESI^+) found ($^{81}BrMNa^+$): 520.0128 $C_{23}H_{18}{}^{81}BrN_3NaO_3S$ requires 520.0126. Found ($^{79}BrMNa^+$): 518.0146 $C_{23}H_{18}{}^{79}BrN_3NaO_3S$ requires 518.0144.

3-Amino-5-(4''-methoxybenzoyl)-*N*-(3'-methoxyphenyl)-6-methylthieno[2,3-*b*]pyridine-2-carboxamide 5g

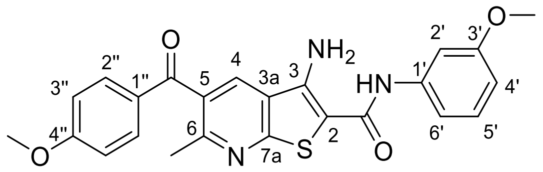

The reaction was carried out following General procedure 3.2.4 using carbonitrile **12b** (100 mg, 0.35 mmol), chloride **14g** (70 mg, 0.35 mmol) and sodium carbonate (74 mg, 0.70 mmol) in EtOH (2.7 mL) to give the *title compound* **5g** (59 mg, 38%) as a yellow solid. m.p. 112-115 °C. δ_H (400 MHz, $(CD_3)_2SO$) 2.54 (3H, s, 6-CH_3), 3.75 (3H, s, 3'-OCH_3), 3.88 (3H, s, 4''-OCH_3), 6.64 (1H, dd, *J* = 8.2, 2.0 Hz, H-4'), 7.11 (2H, dt, *J* = 8.9, 2.4 Hz, H-3''), 7.21 (1H, t, *J* = 8.2 Hz, H-5'), 7.32 (1H, d, *J* = 8.2 Hz, H-6'), 7.39 (1H, t, *J* = 2.0 Hz, H-2'), 7.41 (2H, br s, NH_2), 7.79 (2H, dt, *J* = 8.9, 2.4 Hz, H-2''), 8.52 (1H, s, H-4), 9.44 (1H, br s, NH). δ_C (100 MHz, $(CD_3)_2SO$) 23.2 (6-CH_3), 55.0 (3'-OCH_3), 55.7 (4''-OCH_3), 106.6 (C-2'), 113.2 (C-6'), 114.3 (C-3''), 123.2 (C-3a), 129.1 (C-5'), 129.5 (C-1''), 130.1 (C-5), 130.7 (C-4), 132.5 (C-2''), 153.4 (C-3), 156.7 (C-6), 159.1 (C-3'), 159.3 (C-7a), 163.7 (C-4''), 163.9 (CONH), 194.6 (5-CO). C-2, C-1' and C-4' not observed. ν_{max} (ATR)/cm^{-1} 3408 (N-H amide), 3299 (N-H amine), 2972 (C-H alkane), 1659 (C=O carbonyl), 1582 (C=C aromatic), 1489 (-C-H bending), 1251 (C-N aromatic), 1184 (C-O ether), 1171 (C-O ether). *m/z* (ESI^+): 470 (MNa^+, 100%), 381 (12%), 227 (12%), 101 (10%). HRMS (ESI^+) found (MNa^+): 470.1147 $C_{24}H_{21}N_3NaO_3S$ requires 470.1145.

3-Amino-5-(4''-methoxybenzoyl)-*N*-(3'-chloro-2'-methylphenyl)-6-methylthieno[2,3-*b*]pyridine-2-carboxamide 5h

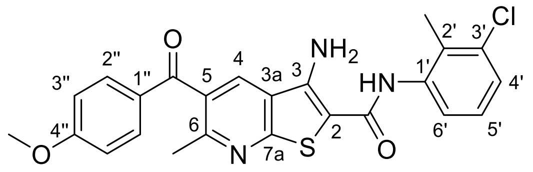

The reaction was carried out following General procedure 3.2.4 using carbonitrile **12b** (100 mg, 0.35 mmol), chloride **14h** (76 mg, 0.35 mmol) and sodium carbonate (74 mg, 0.70 mmol) in EtOH (2.7 mL) to give the *title compound* **5h** (68 mg, 42%) as a brown solid. m.p. 194-196 °C. δ_H (400 MHz, $(CD_3)_2SO$) 2.24 (3H, s, 2'-CH_3), 2.54 (3H, s, 6-CH_3), 3.87 (3H, s, 4''-OCH_3), 7.11 (2H, dt, *J* = 8.9, 2.4 Hz, H-3''), 7.20 (1H, t, *J* = 7.9 Hz, H-5'), 7.29-7.34 (4H, m, H-4', H-6', and NH_2), 7.78 (2H, dt, *J* = 8.9, 2.4 Hz, H-2''), 8.47 (1H, s, H-4), 9.45 (1H, br s, NH). δ_C (100 MHz, $(CD_3)_2SO$) 15.4 (2'-CH_3), 23.2 (6-CH_3), 55.7 (4''-OCH_3), 95.7 (C-2), 114.3 (C-3''), 123.3 (C-3a), 125.8 (C-6'), 126.6 (C-5'), 129.5 (C-1''), 128.7 (C-4'), 130.0 (C-5), 130.6 (C-4), 132.5 (C-2''), 132.2 (C-2'), 133.5 (C-3'), 133.8 (C-1'), 146.2 (C-3), 156.5 (C-6), 159.2 (C-7a), 163.9 (C-4''), 164.0 (CONH), 194.7 (5-CO). ν_{max} (ATR)/cm^{-1} 3438 (N-H amide), 339 (N-H amine), 2972 (C-H alkane), 1657 (C=O amide), 1640 (C=O carbonyl), 1582 (C=C aromatic), 1485 (-C-H bending), 1253 (C-N aromatic), 1169 (C-O ether), 803 (C-Cl). *m/z* (ESI$^+$): 490 (^{37}ClMNa$^+$, 43%), 488 (^{35}ClMNa$^+$, 100%), 431 (10%), 381 (18%), 227 (40%), 159 (24%), 101 (27%). HRMS (ESI$^+$) found (^{37}ClMNa$^+$): 490.0785 $C_{24}H_{20}{}^{37}ClN_3NaO_3S$ requires 490.0784. Found (^{35}ClMNa$^+$): 488.0804 $C_{24}H_{20}{}^{35}ClN_3NaO_3S$ requires 488.0806.

3-Amino-5-(4''-methoxybenzoyl)-*N*-(3'-bromo-2'-methylphenyl)-6-methylthieno[2,3-*b*]pyridine-2-carboxamide 5i

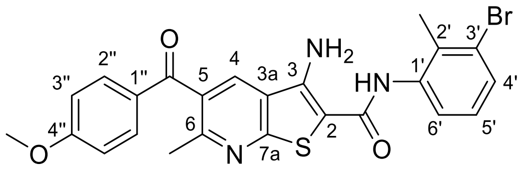

The reaction was carried out following General procedure 3.2.4 using carbonitrile **12b** (100 mg, 0.35 mmol), chloride **14i** (92 mg, 0.35 mmol) and sodium carbonate (74 mg, 0.70 mmol) in EtOH (2.7 mL) to give the *title compound* **5i** (57 mg, 32%) as a brown solid. m.p. 169-171 °C. δ_H (400 MHz, $(CD_3)_2SO$) 2.27 (3H, s, 2'-CH_3), 2.54 (3H, s, 6-CH_3), 3.88 (3H, s, 4''-OCH_3), 7.06-7.17 (2H, m, H-3'' and H-5'), 7.31-7.34 (3H, m, H-6' and NH_2), 7.48 (1H, d, *J* = 8.8 Hz, H-4'), 7.79 (2H, dt, *J* = 9.0, 2.4 Hz, H-2''), 8.49 (1H, s, H-4), 9.49 (1H, br s, NH). δ_C (100 MHz, $(CD_3)_2SO$) 18.5 (2'-CH_3), 23.2 (6-CH_3), 55.7 (4''-OCH_3), 96.2 (C-2), 114.3 (C-3''), 123.4 (C-3a), 124.7 (C-3'), 126.6 (C-6'), 127.1 (C-5'), 129.5 (C-1''), 128.8 (C-4'), 130 (C-5), 130.1 (C-4), 132.5 (C-2''), 133.8 (C-2'), 138.9 (C-1'), 146.1 (C-3), 157.0 (C-6), 159.2 (C-7a), 165.9 (C-4''), 168.7 (CONH), 194.6 (5-CO). ν_{max} (ATR)/cm^{-1} 3407 (N-H amide), 3298 (N-H amine), 2973 (C-H alkane), 1658 (C=O carbonyl), 1583 (C=C aromatic), 1490 (-C-H bending), 1251 (C-N aromatic), 1171 (C-O ether), 693 (C-Br). *m/z* (ESI$^+$): 534 (^{81}BrMNa$^+$, 100%), 532 (^{79}BrMNa$^+$, 94%), 514 (33%), 512 (31%), 413 (14%), 381 (81%), 227 (36%), 179 (25%), 159 (22%), 101 (56%). HRMS (ESI$^+$) found (^{81}BrMNa$^+$): 534.0281 $C_{24}H_{20}{}^{81}BrN_3NaO_3S$ requires 534.0283. Found (^{79}BrMNa$^+$): 532.0302 $C_{24}H_{20}{}^{79}BrN_3NaO_3S$ requires 532.0301.

3-Amino-5-(4''-methoxybenzoyl)-6-methyl-*N*-(napthalen-1'-yl)thieno[2,3-*b*]pyridine-2-carboxamide 5j

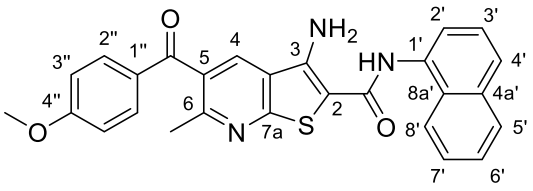

The reaction was carried out following General procedure 3.2.4 using carbonitrile **12b** (100 mg, 0.35 mmol), chloride **14j** (79 mg, 0.35 mmol) and sodium carbonate (74 mg, 0.70 mmol) in EtOH (2.7 mL) to give the *title compound* **5j** (48 mg, 29%) as a mustard solid. m.p. 209-211 °C. δ_H (400 MHz, $(CD_3)_2SO$) 2.57 (3H, s, 6-CH_3), 3.88 (3H, s, 4''-OCH_3), 7.12 (2H, t, J = 8.9 Hz, H-3''), 7.32 (2H, br s, NH_2), 7.51-7.56 (4H, m, Ar-H), 7.80 (2H, d, J = 8.9 Hz, H-2''), 7.84-7.86 (1H, m, Ar-H), 7.94-7.98 (2H, m, Ar-H), 8.52 (1H, s, H-4), 9.76 (1H, br s, NH). δ_C (100 MHz, $(CD_3)_2SO$) 23.2 (6-CH_3), 55.7 (4''-OCH_3), 96.6 (C-2), 114.3 (C-3''), 123.2 (C-3a), 123.5 (Ar-CH), 124.4 (Ar-CH), 125.5 (Ar-CH), 125.9 (Ar-CH), 126.0 (Ar-CH), 126.3 (Ar-CH), 128.0 (Ar-CH), 129.5 (Ar-C), 129.7 (C-1''), 130.1 (C-5), 130.7 (C-4), 132.5 (C-2''), 133.7 (C-1' or Ar-C), 133.8 (C-1' or Ar-C), 146.9 (C-3), 156.7 (C-6), 159.2 (C-7a), 163.6 (C-4''), 164.6 (CONH), 194.6 (5-CO). ν_{max} (ATR)/cm^{-1} 3408 (N-H amide), 3298 (N-H amine), 2973 (C-H alkane), 1659 (C=O carbonyl), 1582 (C=C aromatic), 1489 (-C-H bending), 1251 (C-N aromatic), 1172 (C-O ether). *m/z* (ESI$^+$): 490 (MNa$^+$, 100%), 468 (10%), 227 (12%). HRMS (ESI$^+$) found (MNa$^+$): 490.1214 $C_{27}H_{21}N_3NaO_2S$ requires 490.1196.

3-Amino-6-methyl-*N*-phenyl-5-(4''-(trifluoromethyl)benzoyl)thieno[2,3-*b*]pyridine-2-carboxamide 6a

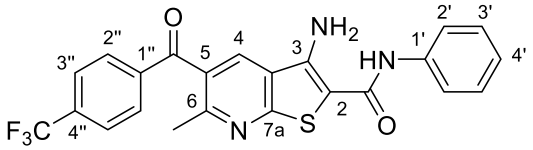

The reaction was carried out following General Procedure 3.2.4 using carbonitrile **12c** (100 mg, 0.31 mmol), chloride **14a** (53 mg, 0.31 mmol) and sodium carbonate (66 mg, 0.62 mmol) in EtOH (2.4 mL) to give the *title compound* **6a** (66 mg, 47 %) as a yellow solid. m.p. >230 °C. ν_{max} (ATR)/cm^{-1} 3186 (N-H amide and N-H amine), 2961 (C-H alkane), 1665 (C=O amide), 1638 (C=O carbonyl), 1589 (C=C aromatic), 1438 (-C-H bending), 1327 (C-F), 1258 (C-N aromatic). δ_H (400 MHz, $(CD_3)_2SO$) 2.64 (3H, s, 6-CH_3), 7.01–7.05 (1H, m, H-4'), 7.29 (2H, t, *J* = 7.7 Hz, H-3'), 7.39 (2H, br s, NH_2), 7.67 (2H, d, *J* = 7.7 Hz, H-2'), 7.96–8.02 (4H, m, H-2'' and H-3''), 8.56 (1H, s, H-4), 9.50 (1H, br s, NH). δ_C (100 MHz, $(CD_3)_2SO$) 23.7 (6-CH_3), 121.1 (C-2'), 123.2 (C-4'), 123.5 (C-3a), 125.9 (C-3''), 128.4 (C-3'), 128.5 (C-5), 130.8 (C-2''), 132.3 (C-4), 138.7 (C-1'), 140.5 (C-1''), 160.0 (C-7a), 163.7 (CONH), 195.4 (5-CO). C-2, C-3, C-6, C-4'', and 4''-CF_3 not observed. *m/z* (ESI$^+$): 410 (MNa^+, 100%), 456 (15%), 431 (18%), 398 (36%), 381 (54%), 363 (23%), 295 (18%), 227 (98%), 159 (54%), 101 (55%). HRMS (ESI$^+$) found (MNa^+): 478.0801 $C_{22}H_{17}F_3N_3NaO_2S$ requires 478.0808.

3-Amino-*N*-(4'-methoxyphenyl)-6-methyl-5-(4''-(trifluoromethyl)benzoyl)thieno[2,3-*b*]pyridine-2-carboxamide 6b

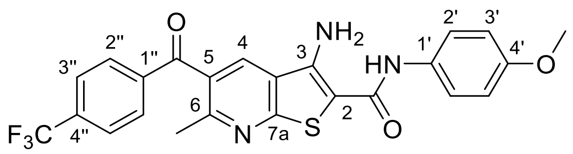

The reaction was carried out following General Procedure 3.2.4 using carbonitrile **12c** (100 mg, 0.31 mmol), chloride **14b** (62 mg, 0.31 mmol) and sodium carbonate (66 mg, 0.62 mmol) in EtOH (2.4 mL) to give the *title compound* **6b** (12 mg, 8%) as a light mustard solid. m.p. 198-200 °C. ν_{max} (ATR)/cm^{-1} 3325 (N-H amide), 3204 (N-H amine), 2930 (C-H alkane), 1662 (C=O amide), 1629 (C=O carbonyl), 1598 (C=C aromatic), 1458 (-C-H bending), 1327 (C-F), 1249 (C-N aromatic), 1167 (C-O ether). δ_H (400 MHz, $(CD_3)_2SO$) 2.64 (3H, s, 6-CH_3), 3.74 (3H, s, 4'-OCH_3), 6.90 (2H, dt, *J* = 9.0, 2.8 Hz, H-3'), 7.37 (2H, br s, NH_2), 7.57 (2H, dt, *J* = 9.0, 2.8 Hz, H-2'), 7.96–8.02 (4H, m, H-2'' and H-3''), 8.58 (1H, s, H-4), 9.40 (1H, br s, NH). δ_C (100 MHz, $(CD_3)_2SO$) 23.7 (6-CH_3), 55.2 (4'-CH_3), 113.6 (C-3'), 122.9 (C-2'), 123.2 (C-3a), 125.8 (C-3''), 128.5 (C-5), 130.8 (C-2''), 132.2 (C-4), 131.8 (C-1'), 140.5 (C-1''), 146.6 (C-3), 155.5 (C-4'), 157.7 (C-6), 160.0 (C-7a), 163.5 (CONH), 195.4 (5-CO). C-2, C-4'', and 4''-CF_3 not observed. *m/z* (ESI^+): 440 (MNa^+, 100%), 381 (31%), 227 (22%), 159 (14%), 101 (18%). HRMS (ESI^+) found (MNa^+): 508.0901 $C_{24}H_{18}F_3N_3NaO_3S$ requires 508.0913.

3-Amino-5-(hydroxy(phenyl)methyl)-6-methyl-*N*-phenylthieno[2,3-*b*]pyridine-2-carboxamide 7a

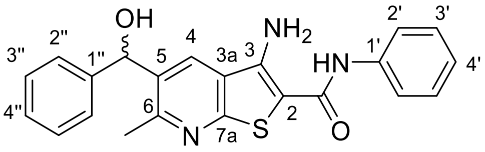

The reaction was carried out following General procedure 3.2.5 using ketone **4a** (50 mg, 0.129 mmol) and $NaBH_4$ (9.8 mg, 0.258 mmol) in THF/MeOH (4:1, 3 mL) to give the *title compound* **7a** (44 mg, 88%) as a yellow solid. m.p. >230 °C. R_f = 0.44 (1:1 EtOAc/Petroleum Ether). δ_H (400 MHz, $(CD_3)_2SO$) 2.47 (3H, s, 6-CH_3), 5.95 (1H, d, *J* = 3.7 Hz, 5-C<u>H</u>OH), 6.13 (1H, d, *J* = 3.7 Hz, 5-CHO<u>H</u>), 7.06 (1H, t, *J* = 7.5 Hz, H-4'), 7.23-7.27 (1H, m, H-4''), 7.29-7.35 (6H, m, H-3', H-3'', and H-2''), 7.43 (2H, br s, NH_2), 7.69 (2H, dd, *J* = 8.4, 0.9 Hz, H-2'), 8.68 (1H, s, H-4), 9.36 (1H, br s, NH). δ_C (100 MHz, $(CD_3)_2SO$) 22.7 (6-CH_3), 71.1 (5-CHOH), 95.8 (C-2), 121.1 (C-2'), 123.3 (C-4'), 124.4 (C-3a), 126.6 (C-4''), 127.1 (C-2''), 128.2 (C-3'), 128.3 (C-3''), 128.9 (C-4), 135.2 (C-5), 139.0 (C-1'), 143.4 (C-1''), 147.4 (C-3), 156.9 (C-6), 160.7 (C-7a), 164.1 (CONH). ν_{max} (ATR)/cm^{-1} 3394 (C-OH), 3296 (N-H amide, and N-H amine), 2921 (C-H alkane), 1979 (C-H aromatic), 1640 (C=O), 1587 (C=C aromatic), 1439 (C-H bending), 1260 (C-N aromatic), 1039 (C-N aliphatic). *m/z* (ESI^+): 390 (MH^+, 100%). HRMS (ESI^+) found (MH^+): 390.1279 $C_{22}H_{20}N_3O_2S$ requires 390.1271.

3-Amino-5-(hydroxy(phenyl)methyl)-*N*-(4'-methoxyphenyl)-6-methylthieno[2,3-*b*]pyridine-2-carboxamide 7b

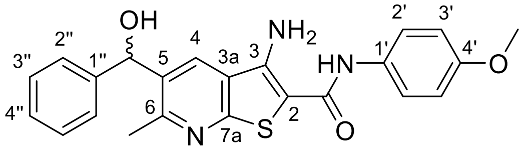

The reaction was carried out following General procedure 3.2.5 using ketone **4b** (35 mg, 0.084 mmol) and $NaBH_4$ (5 mg, 0.144 mmol) in THF/MeOH (4:1, 3 mL) to give the *title compound* **7b** (35 mg, 88%) as a yellow solid. m.p. >230 °C. R_f = 0.53 (1:1 EtOAc/Petroleum Ether). δ_H (400 MHz, $(CD_3)_2SO$) 2.46 (3H, s, 6-CH_3), 3.74 (3H, s, 4'-OCH_3), 5.94 (1H, d, *J* = 3.7 Hz, 5-C<u>H</u>OH), 6.12 (1H, d, *J* = 3.7 Hz, 5-CHO<u>H</u>), 6.89 (2H, dt, *J* = 9.1, 2.8 Hz, H-3'), 7.24 - 7.27 (1H, m, H-4''), 7.33-7.34 (4H, m, H-2'' and H-3''), 7.38 (2H, br s, NH_2), 7.57 (2H, dd, *J* = 9.1, 2.8 Hz, H-2'), 8.65 (1H, s, H-4), 9.26 (1H, br s, NH). δ_C (100 MHz, $(CD_3)_2SO$) 22.7 (6-CH_3), 55.1 (4'-CH_3), 71.1 (5-CHOH), 96.0 (C-2), 113.5 (C-3'), 122.9 (C-2'), 124.4 (C-3a), 127.1 (C-2''), 126.9 (C-4''), 128.2 (C-3''), 128.8 (C-4), 135.2 (C-5), 131.9 (C-1'), 143.4 (C-1''), 147.6 (C-3), 155.5 (C-4'), 156.6 (C-6), 157.9 (C-7a), 163.9 (CONH). ν_{max} (ATR)/cm^{-1} 3447 (C-OH), 3332 (N-H amide), 3264 (N-H amine), 2927 (C-H alkane), 1980 (C-H aromatic), 1638 (C=O), 1596 (C=C aromatic), 1232 (C-N aromatic), 1173 (C-O ether), 1036 (C-N aliphatic). *m/z* (ESI^+): 420 (MH^+, 100%). HRMS (ESI^+) found (MH^+): 420.1377 $C_{23}H_{21}N_3O_3S$ requires 420.1376.

3-Amino-*N*-(4'-chlorophenyl)-5-(hydroxy(phenyl)methyl)-6-methylthieno[2,3-*b*]pyridine-2-carboxamide 7c

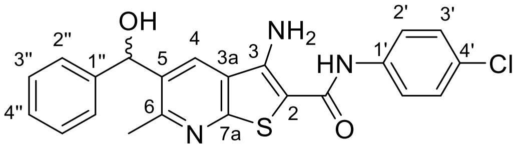

The reaction was carried out following General procedure 3.2.5 using ketone **4c** (35 mg, 0.086 mmol) and $NaBH_4$ (5 mg, 0.086 mmol) in THF/MeOH (4:1, 3 mL) to give the *title compound* **7c** (35 mg, quantitative) as a yellow solid. m.p. >230 °C. R_f = 0.72 (1:1 EtOAc/Petroleum Ether). δ_H (400 MHz, $(CD_3)_2SO$) 2.47 (3H, s, 6-CH_3), 5.95 (1H, d, J = 3.4 Hz, 5-C<u>H</u>OH), 6.13 (1H, d, J = 3.4 Hz, 5-CHO<u>H</u>), 7.24-7.27 (1H, m, H-4''), 7.33-7.35 (4H, m, H-2'' and H-3''), 7.36 (2H, d, J = 8.9 Hz, H-3'), 7.48 (2H, br s, NH_2), 7.74 (2H, d, J = 8.9 Hz, H-2'), 8.69 (1H, s, H-4), 9.49 (1H, br s, NH). δ_C (100 MHz, $(CD_3)_2SO$) 22.7 (6-CH_3), 71.1 (5-CHOH), 95.4 (C-2), 122.4 (C-2'), 124.3 (C-3a), 126.6 (C-4''), 127.1 (C-4'' and C-2''), 128.2 (C-3' and C-3''), 129.0 (C-4), 135.3 (C-5), 138.0 (C-1'), 143.4 (C-1''), 147.7 (C-3), 156.6 (C-6), 157.1 (C-7a), 164.1 (CONH). C-4' was not observed on ^{13}C NMR. ν_{max} (ATR)/cm^{-1} 3450 (C-OH), 3382 (N-H amide), 3240 (N-H amine), 2924 (C-H alkane), 2031 (C-H aromatic), 1640 (C=O), 1590 (C=C aromatic), 1235 (C-N aromatic), 1037 (C-N aliphatic), 771 (C-Cl). *m/z* (ESI$^+$): 448 (^{37}ClMNa$^+$, 40%), 446 (^{35}ClMNa$^+$, 100%). HRMS (ESI$^+$) found (^{37}ClMNa$^+$): 448.0657 $C_{22}H_{18}{}^{37}ClN_3NaO_2S$ requires 448.0677. Found (^{35}ClMNa$^+$): 446.0685 $C_{22}H_{18}{}^{35}ClN_3NaO_2S$ requires 446.0700.

3-Amino-*N*-(4'-bromophenyl)-5-(hydroxy(phenyl)methyl)-6-methylthieno[2,3-*b*]pyridine-2-carboxamide 7d

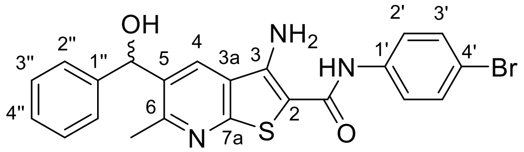

The reaction was carried out following General procedure 3.2.5 using ketone **4d** (30 mg, 0.065 mmol) and $NaBH_4$ (5 mg, 0.129 mmol) in THF/MeOH (4:1, 3 mL) to give the *title compound* **7d** (29 mg, 97%) as a brown solid. m.p. >230 °C. R_f = 0.62 (1:1 EtOAc/Petroleum Ether). δ_H (400 MHz, $(CD_3)_2SO$) 2.46 (3H, s, 6-CH_3), 5.95 (1H, d, *J* = 3.3 Hz, 5-C<u>H</u>OH), 6.13 (1H, d, *J* = 3.3 Hz, 5-CHO<u>H</u>), 7.25-7.27 (1H, m, H-4''), 7.32-7.34 (4H, m, H-2''and H-3''), 7.47- 7.51 (4H, m, NH_2 and H-3'), 7.69 (2H, dt, *J* = 8.9, 2.6 Hz, H-2'), 8.69 (1H, s, H-4), 9.48 (1H, br s, NH). δ_C (100 MHz, $(CD_3)_2SO$) 22.8 (6-CH_3), 71.1 (5-CHOH), 95.5 (C-2), 114.9 (C-4'), 122.8 (C-2'), 124.2 (C-3a), 126.9 (C-4''), 127.1 (C-2''), 128.2 (C-3''), 129.0 (C-4), 131.1 (C-3'), 135.3 (C-5), 138.5 (C-1'), 143.4 (C-1''), 147.8 (C-3), 156.6 (C-6), 157.1 (C-7*a*), 164.1 (CONH). ν_{max} (ATR)/cm^{-1} 3279 (C-OH, N-H amide, and N-H amine), 2923 (C-H alkane), 1979 (C-H aromatic), 1645 (C=O), 1583 (C=C aromatic), 1248 (C-N aromatic), 1041 (C-N aliphatic), 678 (C-Br). *m/z* (ESI^+): 492 ($^{79}BrMNa^+$, 100%), 490 ($^{79}BrMNa^+$, 94%), 449 (22%), 447 (80%), 413 (88%), 381 (47%), 332 (74%), 304 (77%). HRMS (ESI^+) found ($^{81}BrMNa^+$): 492.0179 $C_{22}H_{18}{}^{81}BrN_3NaO_3S$ requires 492.177. Found ($^{79}BrMNa^+$): 490.0202 $C_{22}H_{18}{}^{79}BrN_3NaO_3S$ requires 490.0195.

3-Amino-*N*-(3'-chlorophenyl)-5-(hydroxy(phenyl)methyl)-6-methylthieno[2,3-*b*]pyridine-2-carboxamide 7e

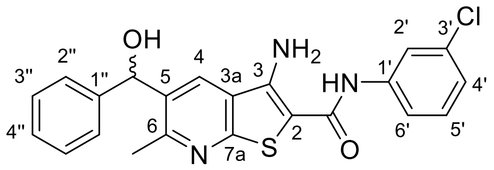

The reaction was carried out following General procedure 3.2.5 using ketone **4e** (30 mg, 0.071 mmol) and $NaBH_4$ (5 mg, 0.142 mmol) in THF/MeOH (4:1, 3 mL) to give the *title compound* **7e** (30 mg, quantitative) as a yellow solid. m.p. >230 °C. R_f = 0.53 (1:1 EtOAc/Petroleum Ether). δ_H (400 MHz, $(CD_3)_2SO$) 2.49 (3H, s, 6-CH_3), 5.95 (1H, d, *J* = 3.6 Hz, 5-C<u>H</u>OH), 6.14 (1H, d, *J* = 3.6 Hz, 5-CHO<u>H</u>), 7.11 (1H, ddd, *J* = 8.1, 1.9, 0.8 Hz, H-4'), 7.25-7.27 (1H, m, H-4''), 7.31-7.36 (5H, m, H-5', H-2'' and H-3''), 7.52 (2H, br s, NH_2), 7.65 (1H, dd, *J* = 8.4, 1.9 Hz, H-6'), 7.91 (1H, t, *J* = 1.9 Hz, H-2'), 8.70 (1H, s, H-4), 9.52 (1H, br s, NH). δ_C (100 MHz, $(CD_3)_2SO$) 22.7 (6-CH_3), 71.1 (5-CHOH), 95.2 (C-2), 119.1 (C-6'), 120.2 (C-2'), 122.8 (C-4'), 124.2 (C-3a), 127.0 (C-4''), 127.1 (C-2''), 129.0 (C-4), 129.5 (C-5'), 130.0 (C-3''), 132.7 (C-3'), 135.3 (C-5), 140.6 (C-1'), 143.3 (C-1''), 148.0 (C-3), 156.6 (C-6), 157.2 (C-7a), 164.2 (CONH). ν_{max} (ATR)/cm^{-1} 3432 (C-OH), 3349 (N-H amide), 3253 (N-H amine), 2924 (C-H alkane), 1980 (C-H aromatic), 1645 (C=O), 1586 (C=C aromatic), 1252 (C-N aromatic), 1042 (C-N aliphatic), 775 (C-Cl). *m/z* (ESI^+): 448 ($^{37}ClMNa^+$, 15%), 446 ($^{35}ClMNa^+$, 42%), 426 (39%), 424 (100%), 413 (13%), 381 (22%), 332 (18%), 304 (20%), 227 (12%), 159 (10%), 101 (12%). HRMS (ESI^+) found ($^{37}ClMNa^+$): 448.0657 $C_{22}H_{18}{}^{37}ClN_3NaO_2S$ requires 448.0677. Found ($^{35}ClMNa^+$): 446.0703 $C_{22}H_{18}{}^{35}ClN_3NaO_2S$ requires 446.0700.

3-Amino-*N*-(3'-bromophenyl)-5-(hydroxy(phenyl)methyl)-6-methylthieno[2,3-*b*]pyridine-2-carboxamide 7f

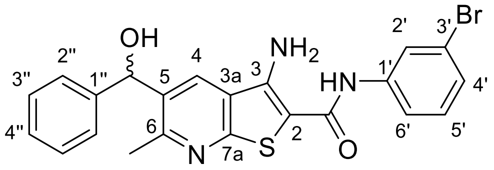

The reaction was carried out following General procedure 3.2.5 using ketone **4f** (30 mg, 0.065 mmol) and $NaBH_4$ (5 mg, 0.129 mmol) in THF/MeOH (4:1, 3 mL) to give the *title compound* **7f** (30 mg, quantitative) as a yellow solid. m.p. >230 °C. R_f = 0.57 (1:1 EtOAc/Petroleum Ether). δ_H (400 MHz, $(CD_3)_2SO$) 2.46 (3H, s, 6-CH_3), 5.94 (1H, s, 5-C$\underline{H}$OH), 6.13 (1H, s, 5-CHO$\underline{H}$), 7.25-7.27 (3H, m, H-4'', H-4' and H-5'), 7.30-7.34 (4H, m, H-2'', and H-3''), 7.52 (2H, br s, NH_2), 7.69 (1H, d, J = 7.4 Hz, H-6'), 8.05 (1H, s, H-2'), 8.69 (1H, s, H-4), 9.50 (1H, br s, NH). δ_C (100 MHz, $(CD_3)_2SO$) 22.8 (6-CH_3), 71.1 (5-CHOH), 95.2 (C-2), 119.5 (C-6'), 121.2 (C-3'), 123.0 (C-2'), 124.3 (C-3a), 125.7 (C-4'), 127.1 (C-2''), 127.2 (C-4''), 128.2 (C-3''), 129.1 (C-4), 130.3 (C-5'), 135.3 (C-5), 140.8 (C-1'), 143.4 (C-1''), 148.0 (C-3), 156.6 (C-6), 157.2 (C-7a), 164.2 (CONH). ν_{max} (ATR)/cm^{-1} 3274 (C-OH, N-H amide, and N-H amine), 2922 (C-H alkane), 1977 (C-H aromatic), 1646 (C=O), 1583 (C=C aromatic), 1248 (C-N aromatic), 1042 (C-N aliphatic), 678 (C-Br). *m/z* (ESI^+): 492 ($^{79}BrMNa^+$, 42%), 490 ($^{79}BrMNa^+$, 40%), 470 (100%), 468 (91%), 413 (23%), 332 (99%), 304 (81%), 117 (27%), 101 (17%). HRMS (ESI^+) found ($^{81}BrMNa^+$): 492.0177 $C_{22}H_{18}{}^{81}BrN_3NaO_3S$ requires 492.177. Found ($^{79}BrMNa^+$): 490.0204 $C_{22}H_{18}{}^{79}BrN_3NaO_3S$ requires 490.0195.

3-Amino-5-(hydroxy(phenyl)methyl)-*N*-(3'-methoxyphenyl)-6-methylthieno[2,3-*b*]pyridine-2-carboxamide 7g

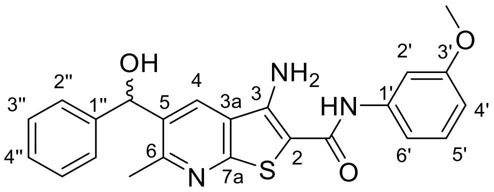

The reaction was carried out following General procedure 3.2.5 using ketone **4g** (30 mg, 0.072 mmol) and $NaBH_4$ (5 mg, 0.143 mmol) in THF/MeOH (4:1, 3 mL) to give the *title compound* **7g** (30 mg, quantitative) as a yellow solid. m.p. decomp. at 192 °C. R_f = 0.54 (1:1 EtOAc/Petroleum Ether). δ_H (400 MHz, $(CD_3)_2SO$) 2.47 (3H, s, 6-CH_3), 3.75 (3H, s, 3'-OCH_3), 5.95 (1H, d, *J* = 3.8 Hz, 5-C<u>H</u>OH), 6.13 (1H, d, *J* = 3.8 Hz, 5-CHO<u>H</u>), 6.64 (1H, ddd, *J* = 8.3, 2.3, 0.7 Hz, H-4'), 7.20 (1H, t, *J* = 8.3 Hz, H-5'), 7.25-7.27 (1H, m, H-4''), 7.31 (1H, dd, *J* = 2.3, 0.9 Hz, H-6'), 7.32-7.35 (2H, m, H-2'' and H-3''), 7.39 (1H, t, *J* = 2.3 Hz, H-2'), 7.45 (2H, br s, NH_2), 8.68 (1H, s, H-4), 9.31 (1H, br s, NH). δ_C (100 MHz, $(CD_3)_2SO$) 22.7 (6-CH_3), 55.0 (3'-OCH_3), 71.1 (5-CHOH), 95.8 (C-2), 106.5 (C-2'), 108.9 (C-4'), 113.1 (C-6'), 124.4 (C-3a), 127.0 (C-4''), 127.1 (C-2''), 128.2 (C-3''), 129.0 (C-4), 129.1 (C-5'), 135.2 (C-5), 140.3 (C-1'), 143.5 (C-1''), 147.5 (C-3), 154.7 (C-6), 156.5 (C-7a), 159.3 (C-3'), 164.1 (CONH). ν_{max} (ATR)/cm^{-1} 3332 (C-OH, N-H amide, and N-H amine), 2924 (C-H alkane), 1978 (C-H aromatic), 1590 (C=C aromatic), 1256 (C-N aromatic), 1042 (C-N aliphatic). *m/z* (ESI^+): 442 (MNa^+, 100%) 101 (11%). HRMS (ESI^+) found (MNa^+): 442.1197 $C_{23}H_{21}N_3NaO_3S$ requires 442.1196.

3-Amino-*N*-(3'-chloro-2'-methylphenyl)-5-(hydroxy(phenyl)methyl)-6-methylthieno[2,3-*b*]pyridine-2-carboxamide 7h

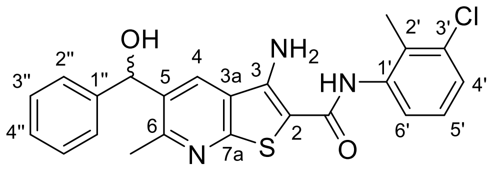

The reaction was carried out following General procedure 3.2.5 using ketone **4h** (30 mg, 0.069 mmol) and $NaBH_4$ (5 mg, 0.138 mmol) in THF/MeOH (4:1, 3 mL) to give the *title compound* **7h** (29 mg, 97%) as a brown solid. m.p. >230 °C. R_f = 0.58 (1:1 EtOAc/Petroleum Ether). δ_H (400 MHz, $(CD_3)_2SO$) 2.23 (3H, s, 2'-CH_3), 2.46 (3H, s, 6-CH_3), 5.95 (1H, d, *J* = 3.6 Hz, 5-C<u>H</u>OH), 6.14 (1H, d, *J* = 3.6 Hz, 5-CHO<u>H</u>), 7.20-7.28 (3H, m, *J* = 7.9 Hz, H-4'', H-5' and H-6'), 7.33-7.34 (4H, m, H-2'' and H-3''), 7.35-7.36 (3H, m, H-4', and NH_2), 8.67 (1H, s, H-4), 9.33 (1H, br s, NH). δ_C (100 MHz, $(CD_3)_2SO$) 15.4 (2'-CH_3), 22.8 (6-CH_3), 71.1 (5-CHOH), 95.6 (C-2), 124.4 (C-3a), 126.2 (C-6'), 126.5 (C-5'), 126.7 (C-4'), 126.8 (C-4''), 127.2 (C-2''), 128.2 (C-3''), 128.9 (C-4), 132.5 (C-2'), 133.6 (C-3'), 135.2 (C-5), 138.2 (C-1'), 143.4 (C-1''), 147.2 (C-3), 156.5 (C-6), 156.9 (C-7a), 164.2 (CONH). ν_{max} (ATR)/cm^{-1} 3426 (C-OH), 3278 (N-H amide), 3173 (N-H amine), 2924 (C-H alkane), 1980 (C-H aromatic), 1678 (C=O), 1588 (C=C aromatic), 1253 (C-N aromatic), 1042 (C-N aliphatic), 778 (C-Cl). *m/z* (ESI^+): 462 ($^{37}ClMNa^+$, 38%), 460 ($^{35}ClMNa^+$, 100%), 438 (23%), 413 (20%), 381 (18%), 332 (31%), 304 (34%), 101 (15%). HRMS (ESI^+) found (^{37}Cl MNa^+): 462.0834 $C_{23}H_{20}{}^{37}ClN_3NaO_2S$ requires 462.0834. Found (^{35}Cl MNa^+): 460.0854 $C_{23}H_{20}{}^{35}ClN_3NaO_2S$ requires 460.0857.

3-Amino-*N*-(3'-bromo-2'-methylphenyl)-5-(hydroxy(phenyl)methyl)-6-methylthieno[2,3-*b*]pyridine-2-carboxamide 7i

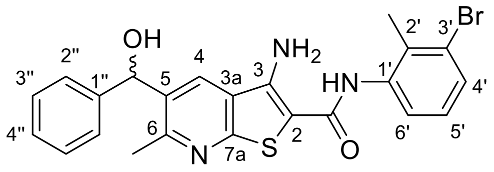

The reaction was carried out following General procedure 3.2.5 using ketone **4i** (30 mg, 0.062 mmol) and $NaBH_4$ (5 mg, 0.125 mmol) in THF/MeOH (4:1, 3 mL) to give the *title compound* **7i** (30 mg, quantitative) as a yellow solid. m.p. >230 °C. R_f = 0.70 (1:1 EtOAc/Petroleum Ether). δ_H (400 MHz, $(CD_3)_2SO$) 2.26 (3H, s, 2'-CH_3), 2.46 (3H, s, 6-CH_3), 5.95 (1H, d, *J* = 3.7 Hz, 5-C<u>H</u>OH), 6.14 (1H, d, *J* = 3.7 Hz, 5-CHO<u>H</u>), 7.16 (1H, t, *J* = 8.0 Hz, H-5'), 7.23-7.27 (1H, m, H-4''), 7.31 (1H, d, *J* = 8.0 Hz, H-6'), 7.33-7.34 (4H, m, H-2'' and H-3''), 7.36 (2H, br s, NH_2), 7.50 (1H, dd, *J* = 8.0, 1.0 Hz, H-4'), 8.67 (1H, s, H-4), 9.35 (1H, br s, NH). δ_C (100 MHz, $(CD_3)_2SO$) 18.5 (2'-CH_3), 22.7 (6-CH_3), 71.1 (5-CHOH), 95.6 (C-2), 124.4 (C-3a), 124.5 (C-3'), 126.5 (C-6'), 126.9 (C-5'), 127.2 (C-2''), 127.2 (C-4''), 128.2 (C-3''), 128.9 (C-4), 129.9 (C-4'), 134.3 (C-2'), 138.0 (C-1'), 143.4 (C-1''), 147.2 (C-3), 164.2 (CONH). C-5, C-6 and C-7a not observed. ν_{max} (ATR)/cm^{-1} 3435 (C-OH), 3419 (N-H amide), 3320 (N-H amine), 2924 (C-H alkane), 1980 (C-H aromatic), 1619 (C=O), 1594 (C=C aromatic), 1241 (C-N aromatic), 1039 (C-N aliphatic), 672 (C-Br). *m/z* (ESI$^+$): 506 (^{79}BrMNa$^+$, 100%), 504(^{79}BrMNa$^+$, 93%), 413 (23%), 332 (71%), 304 (70%), 101 (16%). HRMS (ESI$^+$) found (^{81}BrMNa$^+$): 506.0347 $C_{23}H_{20}{}^{81}BrN_3NaO_3S$ requires 50.0333. Found (^{79}BrMNa$^+$): 504.0361 $C_{23}H_{20}{}^{79}BrN_3NaO_3S$ requires 504.0352.

3-Amino-5-(hydroxy(phenyl)methyl)-6-methyl-*N*-(napthalen-1'-yl)thieno[2,3-*b*]pyridine-2-carboxamide 7j

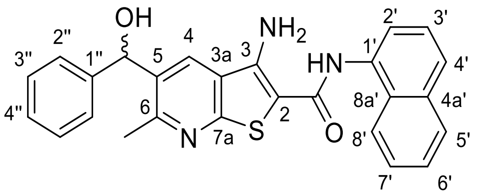

The reaction was carried out following General procedure 3.2.5 using ketone **4j** (30 mg, 0.069 mmol) and $NaBH_4$ (5 mg, 0.137 mmol) in THF/MeOH (4:1, 3 mL) to give the *title compound* **7j** (28 mg, 93%) as a brown solid. m.p. >230 °C. R_f = 0.43 (1:1 EtOAc/Petroleum Ether). δ_H (400 MHz, $(CD_3)_2SO$) 2.48 (3H, s, 6-CH_3), 5.96 (1H, d, *J* = 3.5 Hz, 5-C<u>H</u>OH), 6.15 (1H, d, *J* = 3.5 Hz, 5-CHO<u>H</u>), 7.25-7.28 (1H, m, H-4''), 7.34-7.36 (6H,m, NH_2, H-3'', and H-2''), 7.52-7.56 (4H, m, Ar-H) 7.84-7.87 (1H, m, Ar-H), 7.91-7.93 (1H, m, Ar-H), 7.95-7.98 (1H, m, Ar-H), 8.68 (1H, s, H-4), 9.64 (1H, br s, NH). δ_C (100 MHz, $(CD_3)_2SO$) 22.8 (6-CH_3), 71.1 (5-CHOH), 96.0 (C-2), 123.1 (Ar-CH), 124.4 (Ar-CH), 124.5 (C-3a), 125.5 (Ar-CH), 125.86 (Ar-CH), 125.94 (Ar-CH), 126.2 (Ar-CH), 127.2 (C-2''), 128.0 (Ar-CH), 128.2 (C-3''), 128.9 (C-4), 126.7 (C-4''), 129.7 (Ar-C), 133.7 (C-1' or Ar-CH) 134.0 (C-1' or Ar-CH), 135.2 (C-5), 143.4 (C-1''), 147.1 (C-3), 156.6 (C-6), 156.8 (C-7a), 164.9 (CONH). ν_{max} (ATR)/cm^{-1} 3438 (C-OH), 3345 (N-H amide), 3262 (N-H amine), 2924 (C-H alkane), 1980 (C-H aromatic), 1678 (C=O), 1589 (C=C aromatic), 1255 (C-N aromatic), 1043 (C-N aliphatic). *m/z* (ESI^+): 462 (MNa^+, 100%). HRMS (ESI^+) found (MNa^+): 462.1252 $C_{26}H_{21}N_3NaO_2S$ requires 462.1247.

3-Amino-5-(hydroxy(4''-methoxyphenyl)methyl)-6-methyl-*N*-phenylthieno[2,3-*b*]pyridine-2-carboxamide 8a

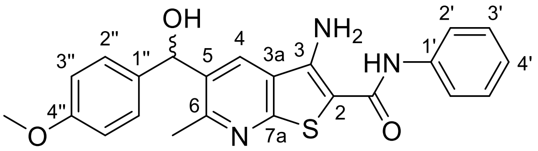

The reaction was carried out following General procedure 3.2.5 using ketone **5a** (25 mg, 0.06 mmol) and $NaBH_4$ (5 mg, 0.119 mmol) in THF/MeOH (4:1, 3 mL) to give the *title compound* **8a** (15 mg, 60%) as a mustard solid. m.p. 225-227 °C. R_f = 0.47 (1:1 EtOAc/Petroleum Ether). δ_H (400 MHz, $(CD_3)_2SO$) 2.44 (3H, s, 6-CH_3), 3.72 (3H, s, 4''-OCH_3), 5.89 (1H, d, *J* = 2.8 Hz, 5-C<u>H</u>OH), 6.01 (1H, d, *J* = 2.8 Hz, 5-CHO<u>H</u>), 6.88 (2H, t, *J* = 8.7, 2.4 Hz, H-3''), 7.06 (1H, t, *J* = 7.6 Hz, H-4'), 7.24 (2H, dt, *J* = 8.7, 2.4 Hz, H-2''), 7.31 (2H, t, *J* = 7.6 Hz, H-3'), 7.43 (2H, br s, NH_2), 7.69 (2H, d, *J* = 7.6 Hz, H-2'), 8.67 (1H, s, H-4), 9.35 (1H, br s, NH). δ_C (100 MHz, $(CD_3)_2SO$) 22.7 (6-CH_3), 55.0 (4''-OCH_3), 70.6 (5-CHOH), 95.8 (C-2), 113.6 (C-3''), 121.1 (C-2'), 123.3 (C-4'), 124.4 (C-3a), 128.3 (C-3'), 128.4 (C-2''), 128.6 (C-4), 135.4 (C-1''), 135.5 (C-5), 139.0 (C-1'), 147.4 (C-3), 156.4 (C-6), 156.9 (C-7a), 158.3 (C-4''), 164.1 (CONH). ν_{max} (ATR)/cm^{-1} 3463 (C-OH), 3385 (N-H amide), 3346 (N-H amine), 2926 (C-H alkane), 2036 (C-H aromatic), 1637 (C=O), 1585 (C=C aromatic), 1442 (C-H bending), 1247 (C-N aromatic), 1174 (C-O ether), 1029 (C-N aliphatic). *m/z* (ESI^+): 442 (MNa^+, 100%), 413 (19%). HRMS (ESI^+) found (MNa^+): 442.1186 $C_{23}H_{21}N_3NaO_3S$ requires 442.1196.

3-Amino-5-(hydroxy(4''-methoxyphenyl)methyl)-*N*-(4'-methoxyphenyl)-6-methylthieno[2,3-*b*]pyridine-2-carboxamide 8b

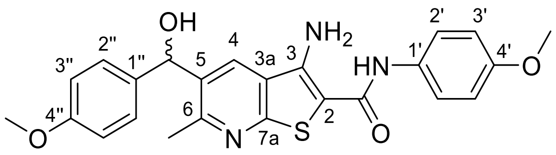

The reaction was carried out following General procedure 3.2.5 using ketone **5b** (25 mg, 0.056 mmol) and $NaBH_4$ (4 mg, 0.112 mmol) in THF/MeOH (4:1, 3 mL) to give the *title compound* **8b** (18 mg, 72%) as a yellow solid. m.p. >230 °C. R_f = 0.44 (1:1 EtOAc/Petroleum Ether). δ_H (400 MHz, $(CD_3)_2SO$) 2.44 (3H, s, 6-CH_3), 3.72 (3H, s, 4''-OCH_3), 3.74 (3H, s, 4'-OCH_3), 5.88 (1H, s, 5-C<u>H</u>OH), 6.00 (1H, s, 5-CHO<u>H</u>), 6.89 (4H, m, H-3' and H-3''), 7.24 (4H, d, *J* = 7.9, H-2''), 7.38 (2H, br s, NH_2), 7.57 (2H, d, *J* = 8.4 Hz, H-2'), 8.65 (1H, s, H-4), 9.25 (1H, br s, NH). δ_C (100 MHz, $(CD_3)_2SO$) 22.7 (6-CH_3), 55.0 (4''-OCH_3), 55.1 (4'-CH_3), 70.6 (5-CHOH), 96.0 (C-2), 113.5 (C-3'), 113.6 (C-3''), 122.9 (C-2'), 124.4 (C-3a), 128.4 (C-2''), 128.5 (C-4), 131.9 (C-1'), 135.4 (C-1''), 135.8 (C-5), 147.0 (C-3), 155.4 (C-4'), 156.3 (C-6), 156.7 (C-7a), 158.3 (C-4''), 163.9 (CONH). ν_{max} (ATR)/cm^{-1} 3459 (C-OH), 3338 (N-H amide and N-H amine), 2928 (C-H alkane), 2031 (C-H aromatic), 1596 (C=C aromatic), 1254 (C-N aromatic), 1172 (C-O ether), 1033 (C-N aliphatic). *m/z* (ESI^+): 472 (MNa^+, 100%), 413 (14%). HRMS (ESI^+) found (MNa^+): 472.1294 $C_{24}H_{23}N_3NaO_4S$ requires 472.1301.

3-Amino-*N*-(4'-chlorophenyl)-5-(hydroxy(4''-methoxyphenyl)methyl)-6-methylthieno[2,3-*b*]pyridine-2-carboxamide 8c

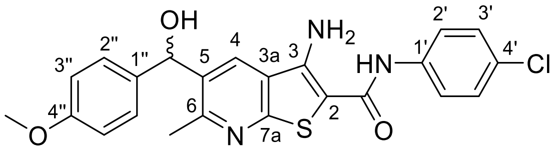

The reaction was carried out following General procedure 3.2.5 using ketone **5c** (30 mg, 0.066 mmol) and $NaBH_4$ (5 mg, 0.133 mmol) in THF/MeOH (4:1, 3 mL) to give the *title compound* **8c** (28 mg, 93%) as a yellow solid. m.p. >230 °C. R_f = 0.54 (1:1 EtOAc/Petroleum Ether). δ_H (400 MHz, $(CD_3)_2SO$) 2.44 (3H, s, 6-CH_3), 3.72 (3H, s, 4''-OCH_3), 5.89 (1H, d, *J* = 3.6 Hz, 5-C<u>H</u>OH), 6.01 (1H, d, *J* = 3.6 Hz, 5-CHO<u>H</u>), 6.88 (2H, d, *J* = 8.8 Hz, H-3''), 7.23 (2H, d, *J* = 8.8 Hz, H-2''), 7.36 and 7.49 (1H, dt, *J* = 8.9, 2.5 Hz, H-3'), 7.48 (2H, m, H-3' and NH_2), 7.69 (1H, d, *J* = 8.9, 2.5 Hz, H-2'), 7.74 (1H, d, *J* = 8.9, 2.5 Hz, H-2'), 8.68 (1H, s, H-4), 9.48 (1H, br s, NH). δ_C (100 MHz, $(CD_3)_2SO$) 22.7 (6-CH_3), 55.7 (4''-OCH_3), 70.6 (5-CHOH), 95.4 (C-2), 113.6 (C-3''), 122.4 (C-2'), 124.3 (C-3a), 126.9 (C-4'), 128.2 (C-3'), 128.4 (C-2''), 128.7 (C-4), 131.2 (C-3'), 135.4 (C-1''), 135.5 (C-5), 138.9 (C-1'), 147.6 (C-3), 156.5 (C-6), 157.1 (C-7a), 158.3 (C-4''), 164.2 (CONH). ν_{max} (ATR)/cm^{-1} 3375 (C-OH), 3346 (N-H amide), 3277 (N-H amine), 2996 (C-H alkane), 2035 (C-H aromatic), 1643 (C=O), 1584 (C=C aromatic), 1252 (C-N aromatic), 1151 (C-O ether), 1031 (C-N aliphatic), 765 (C-Cl). *m/z* (ESI^+): 478 ($^{37}ClMNa^+$, 28%), 476 ($^{35}ClMNa^+$, 71%), 449 (39%), 413 (100%), 381 (89%), 332 (26%), 304 (22%), 227 (64%), 159 (42%), 101 (56%). HRMS (ESI^+) found ($^{37}ClMNa^+$): 478.0774 $C_{23}H_{20}{}^{37}ClN_3NaO_3S$ requires 478.0784. Found ($^{35}ClMNa^+$): 476.0798 $C_{23}H_{20}{}^{35}ClN_3NaO_3S$ requires 476.0806.

3-Amino-*N*-(4'-bromophenyl)-5-(hydroxy(4''-methoxyphenyl)methyl)-6-methylthieno[2,3-*b*]pyridine-2-carboxamide 8d

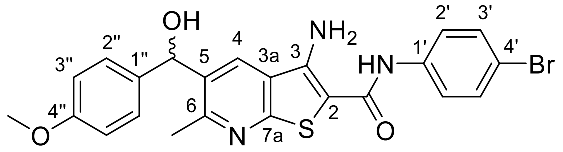

The reaction was carried out following General procedure 3.2.5 using ketone **5d** (25 mg, 0.05 mmol) and $NaBH_4$ (4 mg, 0.101 mmol) in THF/MeOH (4:1, 3 mL) to give the *title compound* **8d** (25 mg, quantitative) as a brown solid. m.p. >230 °C. R_f = 0.41 (1:1 EtOAc/Petroleum Ether). δ_H (400 MHz, $(CD_3)_2SO$) 2.44 (3H, s, 6-CH_3), 3.72 (3H, s, 4''-OCH_3), 5.89 (1H, d, J = 3.5 Hz, 5-C$\underline{H}$OH), 6.01 (1H, d, J = 3.5 Hz, 5-CHO$\underline{H}$), 6.88 (2H, dt, J = 8.8, 2.4 Hz, H-3''), 7.23 (2H, dt, J = 8.8, 2.5 Hz, H-2''), 7.47- 7.51 (4H, m, NH_2 and H-3'), 7.69 (2H, dt, J = 9.0, 2.6 Hz, H-2'), 8.68 (1H, s, H-4), 9.48 (1H, br s, NH). δ_C (100 MHz, $(CD_3)_2SO$) 22.7 (6-CH_3), 55.0 (4''-OCH_3), 70.6 (5-CHOH), 95.4 (C-2), 113.6 (C-3''), 114.9 (C-4'), 122.8 (C-2'), 124.3 (C-3a), 128.4 (C-2''), 128.7 (C-4), 131.1 (C-3'), 135.4 (C-1''), 135.5 (C-5), 138.5 (C-1'), 147.8 (C-3), 156.5 (C-6), 157.1 (C-7a), 158.3 (C-4''), 164.1 (CONH). ν_{max} (ATR)/cm^{-1} 3374 (C-OH), 3220 (N-H amide and N-H amine), 2927 (C-H alkane), 2036 (C-H aromatic), 1644 (C=O), 1583 (C=C aromatic), 1251 (C-N aromatic), 1151 (C-O ether), 1031 (C-N aliphatic), 666 (C-Br). *m/z* (ESI^+): 522 ($^{81}BrMNa^+$, 100%), 520 ($^{79}BrMNa^+$, 93%), 500 (23%), 498 (19%), 449 (41%), 413 (91%), 398 (28%), 381 (32%), 295 (10%), 227 (39%), 159 (26%), 101 (39%). HRMS (ESI^+) found ($^{81}BrMNa^+$): 522.0261 $C_{23}H_{20}{}^{81}BrN_3NaO_3S$ requires 522.0283. Found ($^{79}BrMNa^+$): 520.0283 $C_{23}H_{20}{}^{79}BrN_3NaO_3S$ requires 520.0301.

3-Amino-*N*-(3'-chlorophenyl)-5-(hydroxy(4''-methoxyphenyl)methyl)-6-methylthieno[2,3-*b*]pyridine-2-carboxamide 8e

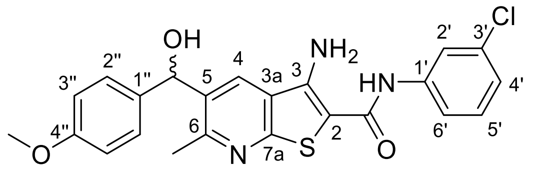

The reaction was carried out following General procedure 3.2.5 using ketone **5e** (25 mg, 0.055 mmol) and $NaBH_4$ (4 mg, 0.11 mmol) in THF/MeOH (4:1, 3 mL) to give the *title compound* **8e** (30 mg, quantitative) as a yellow solid. m.p. 217-220 °C. R_f = 0.66 (1:1 EtOAc/Petroleum Ether). δ_H (400 MHz, $(CD_3)_2SO$) 2.44 (3H, s, 6-CH_3), 3.72 (3H, s, 4''-OCH_3), 5.89 (1H, d, *J* = 3.5 Hz, 5-C<u>H</u>OH), 6.01 (1H, d, *J* = 3.5 Hz, 5-CHO<u>H</u>), 6.88 (2H, dt, *J* = 8.7, 2.2 Hz, H-3''), 7.11 (1H, dt, *J* = 8.2, 1.7 Hz, H-4'), 7.23 (2H, dt, *J* = 8.7, 2.3 Hz, H-2''), 7.34 (1H, t, *J* = 8.2 Hz, H-5'), 7.52 (2H, br s, NH_2), 7.65 (1H, d, *J* = 8.2 Hz, H-6'), 7.91 (1H, t, *J* = 1.7 Hz, H-2'), 8.69 (1H, s, H-4), 9.51 (1H, br s, NH). δ_C (100 MHz, $(CD_3)_2SO$) 22.7 (6-CH_3), 55.0 (4''-OCH_3), 70.6 (5-CHOH), 95.2 (C-2), 113.6 (C-3''), 119.1 (C-6'), 120.2 (C-2'), 122.8 (C-4'), 124.2 (C-3a), 128.4 (C-2''), 128.8 (C-4), 130.0 (C-5'), 132.7 (C-3'), 135.3 (C-1''), 135.6 (C-5), 140.7 (C-1'), 148.0 (C-3), 156.3 (C-6), 157.2 (C-7a), 158.3 (C-4''), 164.2 (CONH). ν_{max} (ATR)/cm^{-1} 3489 (C-OH), 3347 (N-H amide), 3279 (N-H amine), 2927 (C-H alkane), 2041 (C-H aromatic), 1641 (C=O), 1584 (C=C aromatic), 1252 (C-N aromatic), 1174 (C-O ether), 1074 (C-N aliphatic), 757 (C-Cl). *m/z* (ESI$^+$): 478 (^{37}ClMNa$^+$, 42%), 476 (^{35}ClMNa$^+$, 100%), 413 (66%), 381 (53%), 332 (21%), 305 (37%), 227 (62%), 159 (35%), 101 (46%). HRMS (ESI$^+$) found (^{37}ClMNa$^+$): 478.00789 $C_{23}H_{20}{}^{37}ClN_3NaO_3S$ requires 478.0784. Found (^{35}ClMNa$^+$): 476.0816 $C_{23}H_{20}{}^{35}ClN_3NaO_3S$ requires 476.0806.

3-Amino-*N*-(3'-bromophenyl)-5-(hydroxy(4''-methoxyphenyl)methyl)-6-methylthieno[2,3-*b*]pyridine-2-carboxamide 8f

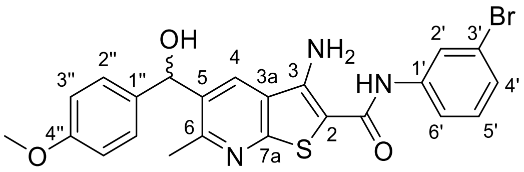

The reaction was carried out following General procedure 3.2.5 using ketone **5f** (25 mg, 0.05 mmol) and $NaBH_4$ (4 mg, 0.101 mmol) in THF/MeOH (4:1, 3 mL) to give the *title compound* **8f** (28 mg, quantitative) as a brown solid. m.p. 140-142 °C. R_f = 0.58 (1:1 EtOAc/Petroleum Ether). δ_H (400 MHz, $(CD_3)_2SO$) 2.44 (3H, s, 6-CH_3), 3.72 (3H, s, 4''-OCH_3), 5.89 (1H, d, *J* = 3.6 Hz, 5-C<u>H</u>OH), 6.01 (1H, d, *J* = 3.6 Hz, 5-CHO<u>H</u>), 6.88 (2H, dt, *J* = 8.8, 2.2 Hz, H-3''), 7.23 – 7.25 (3H, m, H-4', and H-2''), 7.28 (1H, t, *J* = 7.8 Hz, H-5'), 7.52 (2H, br s, NH_2), 7.70 (1H, dt, *J* = 7.8, 1.7 Hz, H-6'), 8.05 (1H, t, *J* = 1.7 Hz, H-2'), 8.69 (1H, s, H-4), 9.50 (1H, br s, NH). δ_C (100 MHz, $(CD_3)_2SO$) 22.7 (6-CH_3), 55.0 (4''-OCH_3), 70.6 (5-CHOH), 95.6 (C-2), 113.6 (C-3''), 119.5 (C-6'), 121.4 (C-3'), 122.8 (C-2'), 124.2 (C-3a), 125.7 (C-4'), 127.7 (C-2''), 128.4 (C-4), 129.5 (C-5'), 135.3 (C-1''), 135.7 (C-5), 140.8 (C-1'), 148.0 (C-3), 157.3 (C-6), 157.6 (C-7a), 158.3 (C-4''), 164.6 (CONH). ν_{max} (ATR)/cm^{-1} 3418 (C-OH), 3320 (N-H amide), 3211 (N-H amine), 2928 (C-H alkane), 2040 (C-H aromatic), 1598 (C=O), 1572 (C=C aromatic), 1251 (C-N aromatic), 1172 (C-O ether), 1074 (C-N aliphatic), 673 (C-Br). *m/z* (ESI^+): 522 ($^{81}BrMNa^+$, 100%), 520 ($^{79}BrMNa^+$, 91%), 500 (33%), 498 (31%), 447 (26%), 413 (34%), 381 (45%), 304 (40%), 270 (26%), 227 (55%), 159 (35%), 101 (50%). HRMS (ESI^+) found ($^{81}BrMNa^+$): 522.0259 $C_{23}H_{20}{}^{81}BrN_3NaO_3S$ requires 522.0283. Found ($^{79}BrMNa^+$): 520.0289 $C_{23}H_{20}{}^{79}BrN_3NaO_3S$ requires 520.0301.

3-Amino-5-(hydroxy(4''-methoxyphenyl)methyl)-*N*-(3'-methoxyphenyl)-6-methylthieno[2,3-*b*]pyridine-2-carboxamide 8g

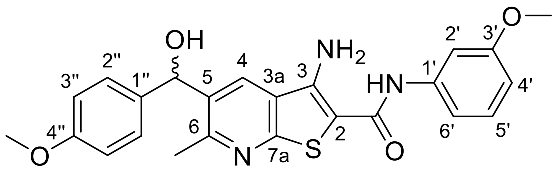

The reaction was carried out following General procedure 3.2.5 using ketone **5g** (25mg, 0.056 mmol) and $NaBH_4$ (4 mg, 0.112 mmol) in THF/MeOH (4:1, 3 mL) to give the *title compound* **8g** (13 mg, 52%) as a mustard solid. m.p. >230 °C. R_f = 0.32 (1:1 EtOAc/Petroleum Ether). δ_H (400 MHz, $(CD_3)_2SO$) 2.44 (3H, s, 6-CH_3), 3.72 (3H, s, 4''-OCH_3), 3.75 (3H, s, 3'-CH_3), 5.89 (1H, d, *J* = 3.5 Hz, 5-C<u>H</u>OH), 6.00 (1H, d, *J* = 3.5 Hz, 5-CHO<u>H</u>), 6.64 (1H, d, *J* = 8.5 Hz, H-4'), 6.88 (2H, dt, *J* = 8.8, 2.4 Hz, H-3''), 7.18-7.25 3, m, H-5' and H-2''), 7.33 (1H, d, *J* = 8.5 Hz, H-6'), 7.38 (1H, t, *J* = 2.2 Hz, H-2'), 7.45 (2H, br s, NH_2), 8.67 (1H, s, H-4), 9.31 (1H, br s, NH). δ_C (100 MHz, $(CD_3)_2SO$) 22.7 (6-CH_3), 55.0 (3'-OCH_3), 55.0 (4''-OCH_3), 70.6 (5-CHOH), 95.8 (C-2), 106.5 (C-2'), 109.0 (C-4'), 113.1 (C-6'), 113.6 (C-3''), 124.3 (C-3a), 128.4 (C-2''), 128.6 (C-4), 129.1 (C-5'), 135.4 (C-5), 135.5 (C-1''), 140.3 (C-1'), 147.5 (C-3), 156.1 (C-6), 157.0 (C-7a), 158.3 (C-3'), 159.3 (C-4''), 164.1 (CONH). ν_{max} (ATR)/cm^{-1} 3411 (C-OH), 3336 (N-H amide), 3299 (N-H amine), 2932 (C-H alkane), 2037 (C-H aromatic), 1658 (C=O), 1588 (C=C aromatic), 1252 (C-N aromatic), 1172 (C-O ether), 1037 (C-N aliphatic). *m/z* (ESI^+): 472 (MNa^+, 100%) 413(10%), 227 (10%), 101 (10%). HRMS (ESI^+) found (MNa^+): 472.1295 $C_{24}H_{23}N_3NaO_4S$ requires 472.1301.

3-Amino-*N*-(3'-chloro-2'-methylphenyl)-5-(hydroxy(4''-methoxyphenyl)methyl)-6-methylthieno[2,3-*b*]pyridine-2-carboxamide 8h

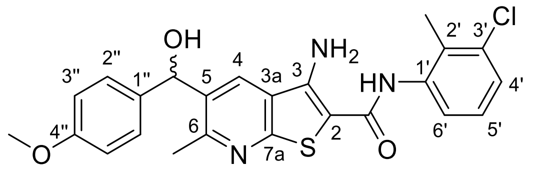

The reaction was carried out following General procedure 3.2.5 using ketone **5h** (25 mg, 0.054 mmol) and $NaBH_4$ (4 mg, 0.107 mmol) in THF/MeOH (4:1, 3 mL) to give the *title compound* **8h** (21 mg, 84%) as a brown solid. m.p. >230 °C. R_f = 0.44 (1:1 EtOAc/Petroleum Ether). δ_H (400 MHz, $(CD_3)_2SO$) 2.23 (3H, s, 2'-CH_3), 2.43 (3H, s, 6-CH_3), 3.72 (3H, s, 4''-OCH_3), 5.89 (1H, d, *J* = 3.3 Hz, 5-C<u>H</u>OH), 6.01 (1H, d, *J* = 3.3 Hz, 5-CHO<u>H</u>), 6.88 (2H, d, *J* = 8.6 Hz, H-3''), 7.22-7.28 (4H, m, H-2'' and H-5' and H-4' or H-6'), 7.32-7.40 (3H, m, NH_2 and H-4' or H-6'), 8.67 (1H, s, H-4), 9.32 (1H, br s, NH). δ_C (100 MHz, $(CD_3)_2SO$) 15.4 (2'-CH_3), 22.7 (6-CH_3), 55.0 (4''-OCH_3), 70.6 (5-CHOH), 95.6 (C-2), 113.6 (C-3''), 124.4 (C-3a), 126.2 (C-2''), 126.5 (C-5'), 126.7 (C-6'), 128.5 (C-4), 132.5 (C-4'), 132.5 (C-2'), 133.6 (C-3'), 135.4 (C-5), 135.5 (C-1''), 138.2 (C-1'), 147.3 (C-3), 156.4 (C-6), 156.9 (C-7a), 158.3 (C-4''), 164.2 (CONH). ν_{max} (ATR)/cm^{-1} 3407 (C-OH), 3300 (N-H amide and N-H amine), 2932 (C-H alkane), 2081 (C-H aromatic), 1659 (C=O), 1592 (C=C aromatic), 1251 (C-N aromatic), 1171 (C-O ether), 1024 (C-N aliphatic), 788 (C-Cl). *m/z* (ESI^+): 492 ($^{37}ClMNa^+$, 41%), 490 ($^{35}ClMNa^+$, 100%), 468 (20%), 413 (17%), 381 (12%), 332 (20%), 304 (20%), 227 (18%), 159 (10%), 101 (15%). HRMS (ESI^+) found ($^{37}ClMNa^+$): 492.0931 $C_{24}H_{22}{}^{37}ClN_3NaO_3S$ requires 492.0941. Found ($^{35}ClMNa^+$): 490.0949 $C_{24}H_{22}{}^{35}ClN_3NaO_3S$ requires 490.0963.

3-Amino-*N*-(3'-bromo-2'-methylphenyl)-5-(hydroxy(4''-methoxyphenyl)methyl)-6-methylthieno[2,3-*b*]pyridine-2-carboxamide 8i

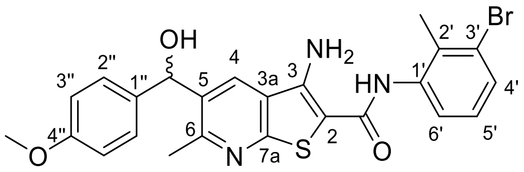

The reaction was carried out following General procedure 3.2.5 using ketone **5i** (25 mg, 0.049 mmol) and $NaBH_4$ (4 mg, 0.098 mmol) in THF/MeOH (4:1, 3 mL) to give the *title compound* **8i** (10 mg, 40%) as a yellow solid. m.p. >230 °C. R_f = 0.44 (1:1 EtOAc/Petroleum Ether). δ_H (400 MHz, $(CD_3)_2SO$) 2.26 (3H, s, 2'-CH_3), 2.43 (3H, s, 6-CH_3), 3.72 (3H, s, 4''-OCH_3), 5.89 (1H, d, *J* = 3.4 Hz, 5-C<u>H</u>OH), 6.01 (1H, d, *J* = 3.4 Hz, 5-CHO<u>H</u>), 6.88(2H, d, *J* = 8.6 Hz, H-3''), 7.16 (1H, t, *J* = 7.8 Hz, H-5'), 7.23 (2H, d, *J* = 8.6 Hz, H-2''), 7.30 (1H, d, *J* = 7.8 Hz, H-6'), 7.36 (2H, br s, NH_2), 7.51 (1H, d, *J* = 7.8 Hz, H-4'), 8.67 (1H, s, H-4), 9.34 (1H, br s, NH). δ_C (100 MHz, $(CD_3)_2SO$) 18.5 (2'-CH_3), 22.7 (6-CH_3), 55.0 (4''-OCH_3), 70.3 (5-CHOH), 95.7 (C-2), 113.6 (C-3''), 124.4 (C-3a), 124.5 (C-3'), 126.9 (C-6'), 127.2 (C-5'), 128.5 (C-2''), 128.7 (C-4), 129.9 (C-4'), 134.0 (C-2'), 134.3 (C-5), 135.4 (C-1''), 138.3 (C-1'), 147.3 (C-3), 156.4 (C-6), 157.0 (C-7a), 158.3 (C-4''), 164.2 (CONH). v_{max} (ATR)/cm^{-1} 3407 (C-OH), 3297 (N-H amide and N-H amine), 2932 (C-H alkane), 2037 (C-H aromatic), 1641 (C=O), 1582 (C=C aromatic), 1251 (C-N aromatic), 1172 (C-O ether), 1037 (C-N aliphatic), 681 (C-Br). *m/z* (ESI^+): 536 ($^{81}BrMNa^+$, 36%), 534 ($^{79}BrMNa^+$, 34%), 449 (43%), 413 (100%), 381 (14%), 332 (10%), 304 (10%), 227 (12%), 101 (15%). HRMS (ESI^+) found ($^{81}BrMNa^+$): 536.0430 $C_{24}H_{22}{}^{81}BrN_3NaO_3S$ requires 536.0439. Found ($^{79}BrMNa^+$): 534.0449 $C_{24}H_{22}{}^{79}BrN_3NaO_3S$ requires 534.0457.

3-Amino-5-(hydroxy(4''-methoxyphenyl)methyl)-6-methyl-*N*-(napthalen-1'-yl)thieno[2,3-*b*]pyridine-2-carboxamide 8j

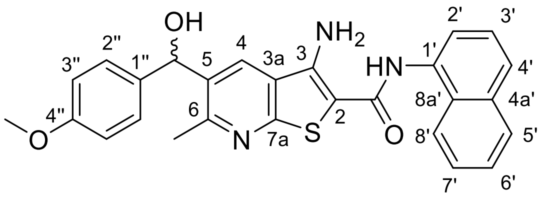

The reaction was carried out following General procedure 3.2.5 using ketone **5j** (25 mg, 0.05 mmol) and $NaBH_4$ (4 mg, 0.101 mmol) in THF/MeOH (4:1, 3 mL) to give the *title compound* **8j** (24 mg, 96%) as a brown solid. m.p. decomp. at 211 °C. R_f = 0.53 (1:1 EtOAc/Petroleum Ether). δ_H (400 MHz, $(CD_3)_2SO$) 2.45 (3H, s, 6-CH_3), 3.73 (3H, s, 4''-OCH_3), 5.91 (1H, d, *J* = 3.6 Hz, 5-C$\underline{H}$OH), 6.02 (1H, d, *J* = 3.6 Hz, 5-CHO$\underline{H}$), 6.89 (2H, d, *J* = 8.7 Hz, H-3''), 7.24 (2H, d, *J* = 8.7 Hz, H-2''), 7.36 (2H, br s, $NH_{2,}$), 7.52-7.56 (4H, m, Ar-H), 7.83-7.88 (1H, m, Ar-H), 7.91-7.94 (1H, m, Ar-H), 7.95 – 7.97 (1H, m, Ar-H), 8.68 (1H, s, H-4), 9.64 (1H, br s, NH). δ_C (100 MHz, $(CD_3)_2SO$) 22.7 (6-CH_3), 55.0 (4''-OCH_3), 70.6 (5-CHOH), 96.0 (C-2), 113.6 (C-3''), 123.5 (Ar-CH), 124.3(Ar-CH), 124.4 (C-3a), 125.5 (Ar-CH), 125.85 (Ar-CH), 125.94 (Ar-CH), 126.2 (Ar-CH), 128.0 (Ar-CH), 128.5 (C-2''), 128.6 (C-4), 129.7 (Ar-C), 133.7 (C-1' or Ar-C), 134.0 (C-1' or Ar-C), 135.1 (C-1''), 135.4 (C-5), 147.2 (C-3), 156.5 (C-6), 156.8 (C-7a), 158.3 (C-4''), 165.0 (CONH). ν_{max} (ATR)/cm^{-1} 3407 (C-OH), 3297 (N-H amide and N-H amine), 2932 (C-H alkane), 2037 (C-H aromatic), 1658 (C=O), 1584 (C=C aromatic), 1254 (C-N aromatic), 1173 (C-O ether), 1072 (C-N).*m/z* (ESI^+): 492 (MNa^+, 100%), 413 (13%). HRMS (ESI^+) found (MNa^+): 492.1341 $C_{21}H_{23}N_3NaO_3S$ requires 492.1352.

^{1}H and ^{13}C NMR spectra for compounds 4a-j, 5a-j, 6a-b, 7a-j and 8a-j:

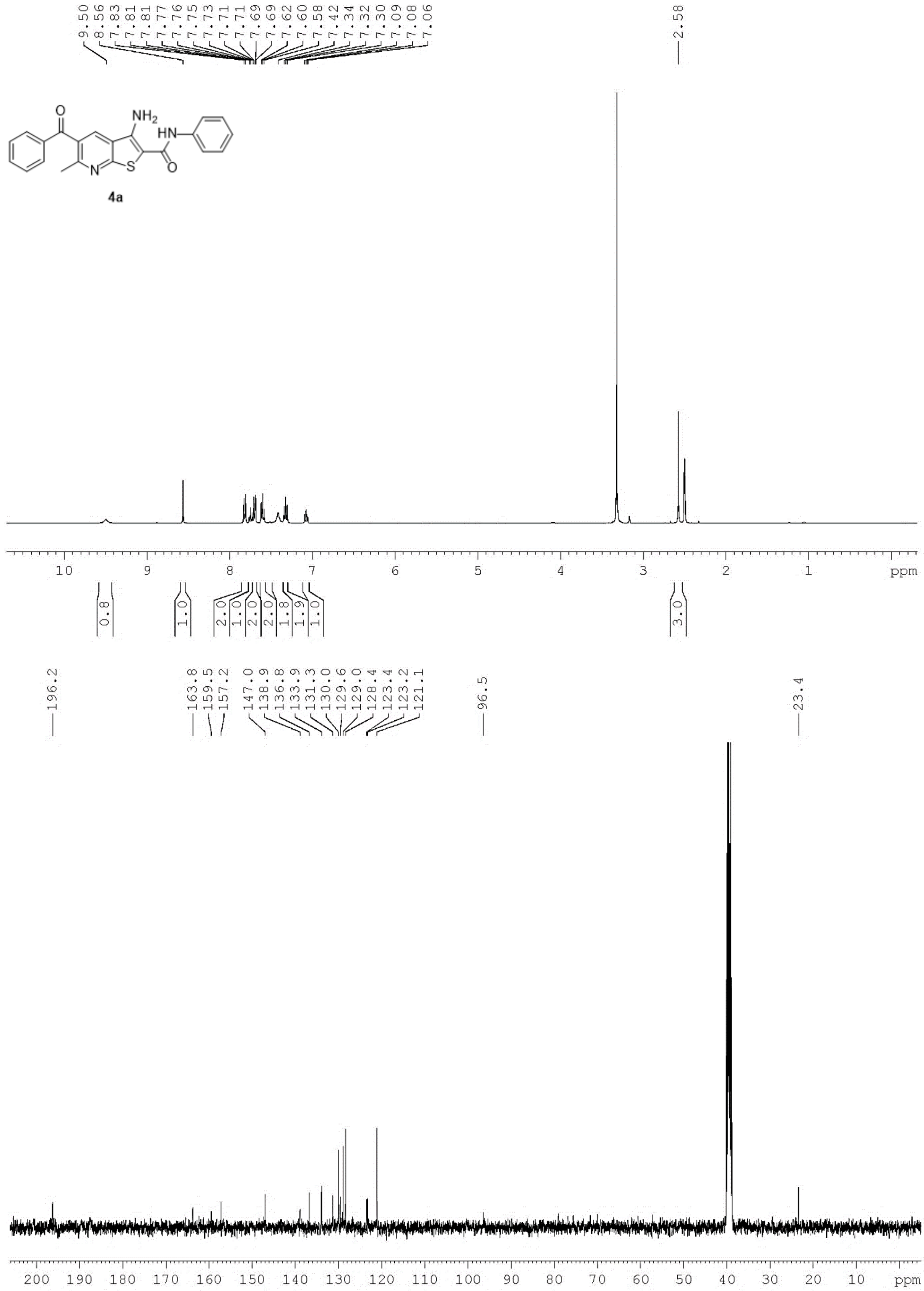

Figure S1: ^{1}H NMR and ^{13}C NMR spectra for **4a**.

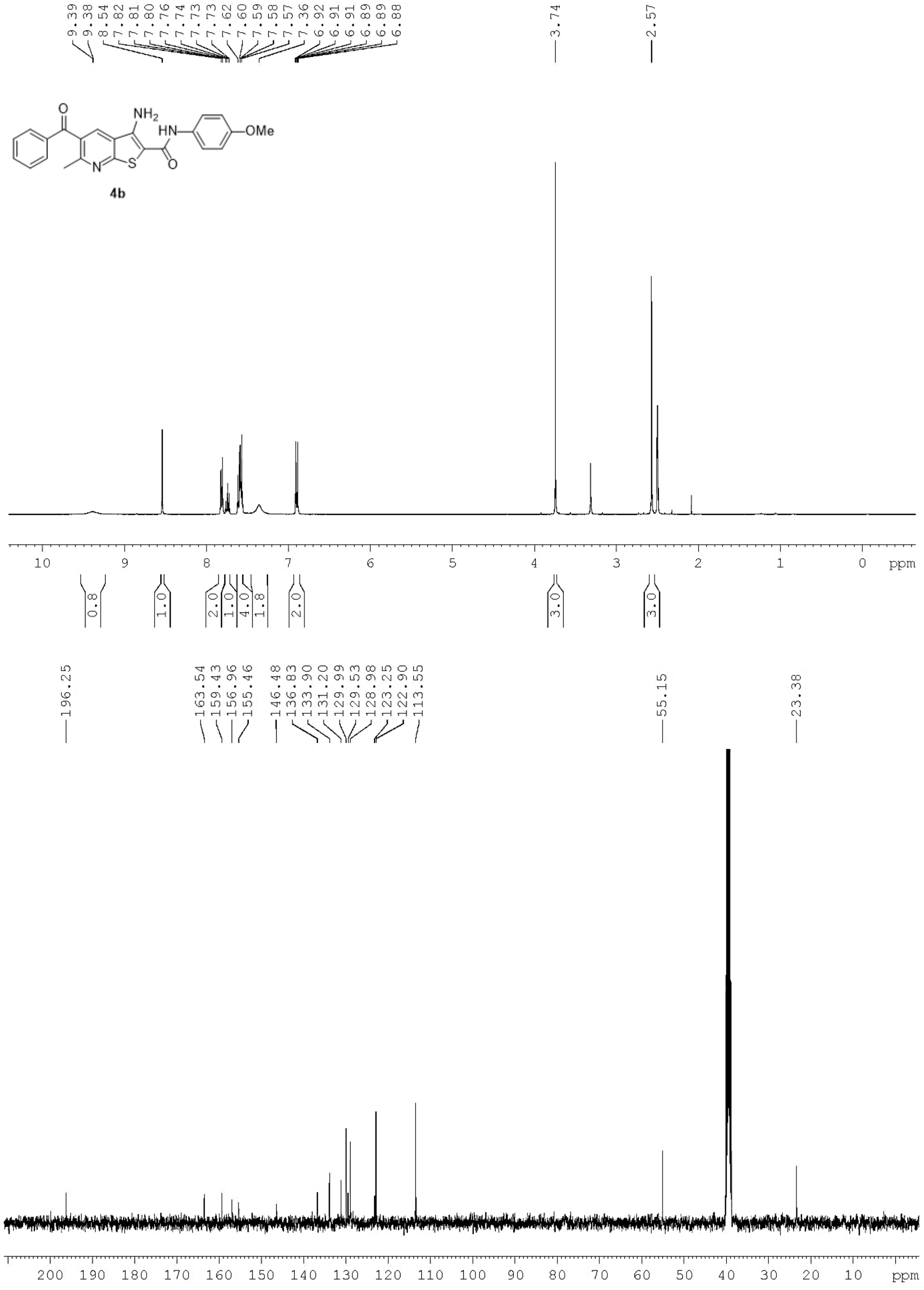

Figure S2: ^{1}H NMR and ^{13}C NMR spectra for **4b**.

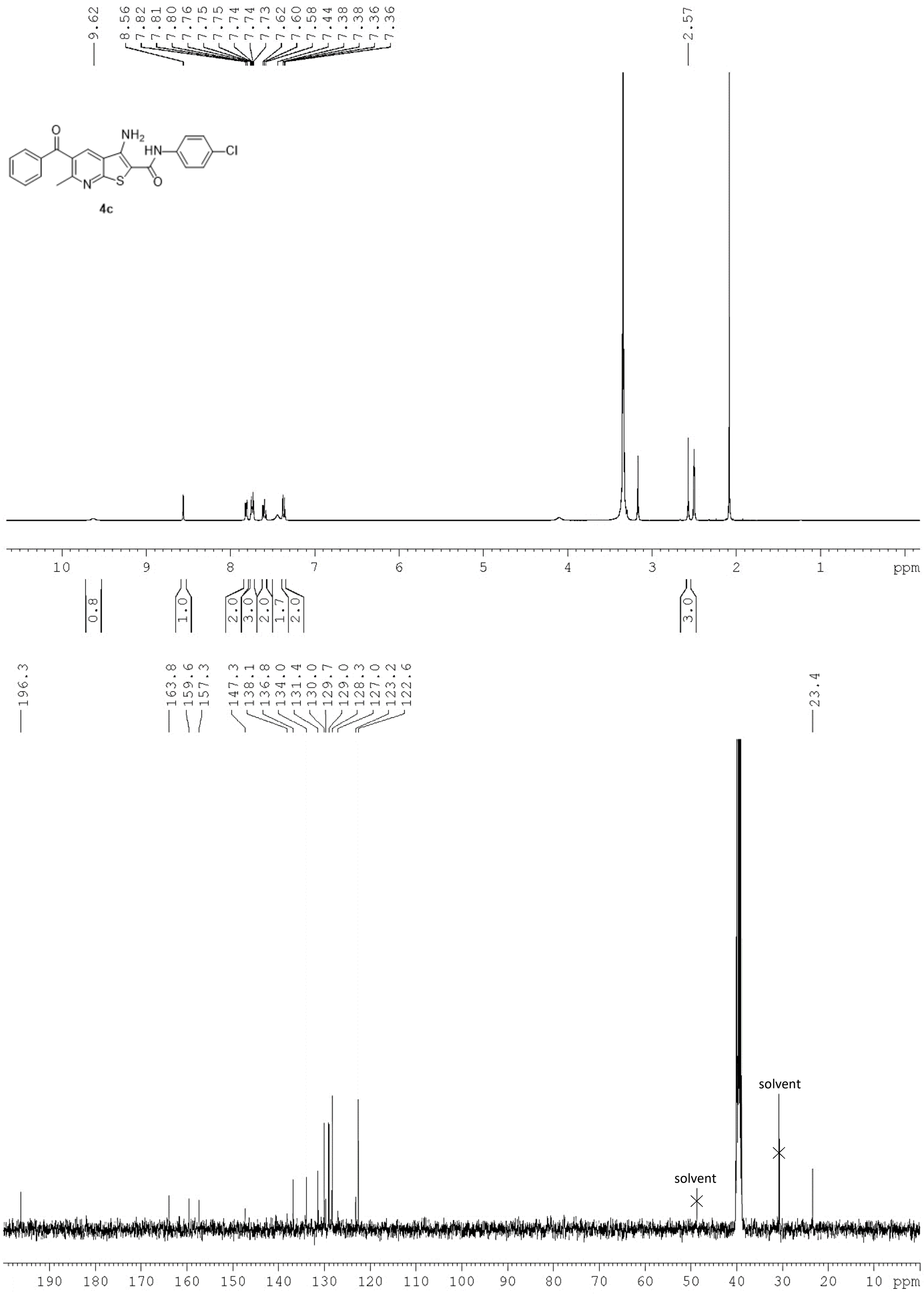

Figure S3: ^{1}H NMR and ^{13}C NMR spectra for **4c**.

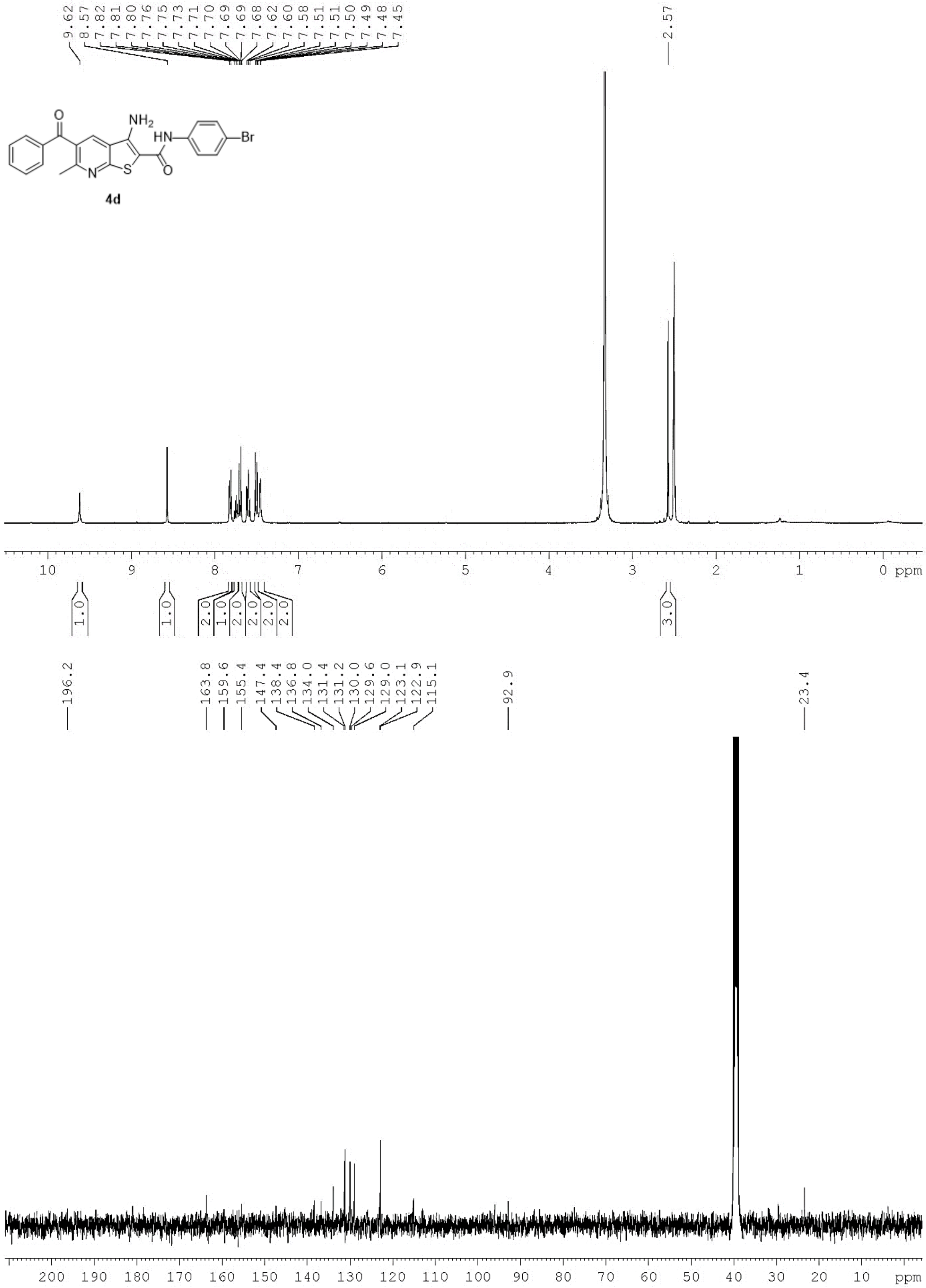

Figure S4: ^{1}H NMR and ^{13}C NMR spectra for **4d**.

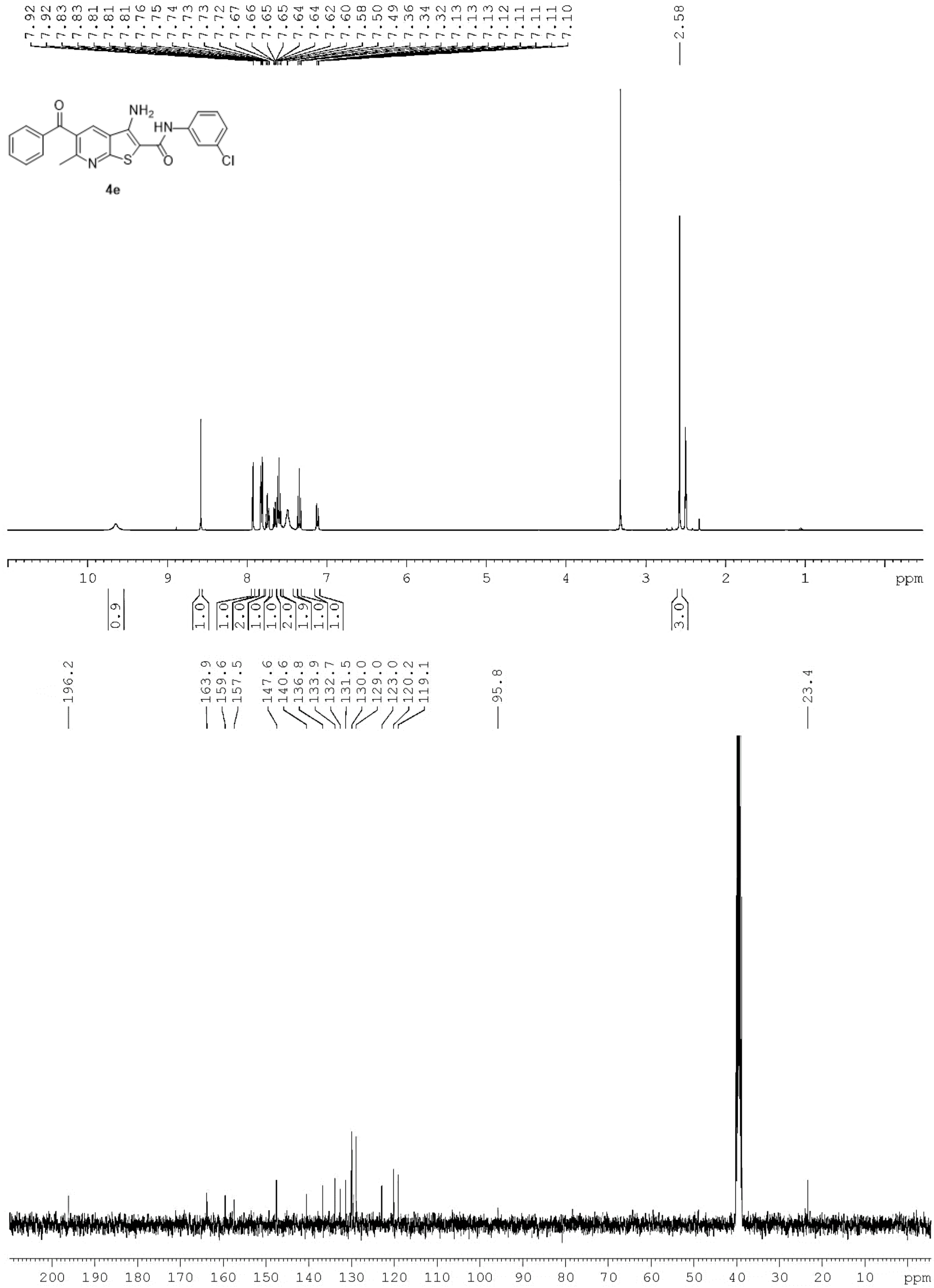

Figure S5: ^{1}H NMR and ^{13}C NMR spectra for **4e**.

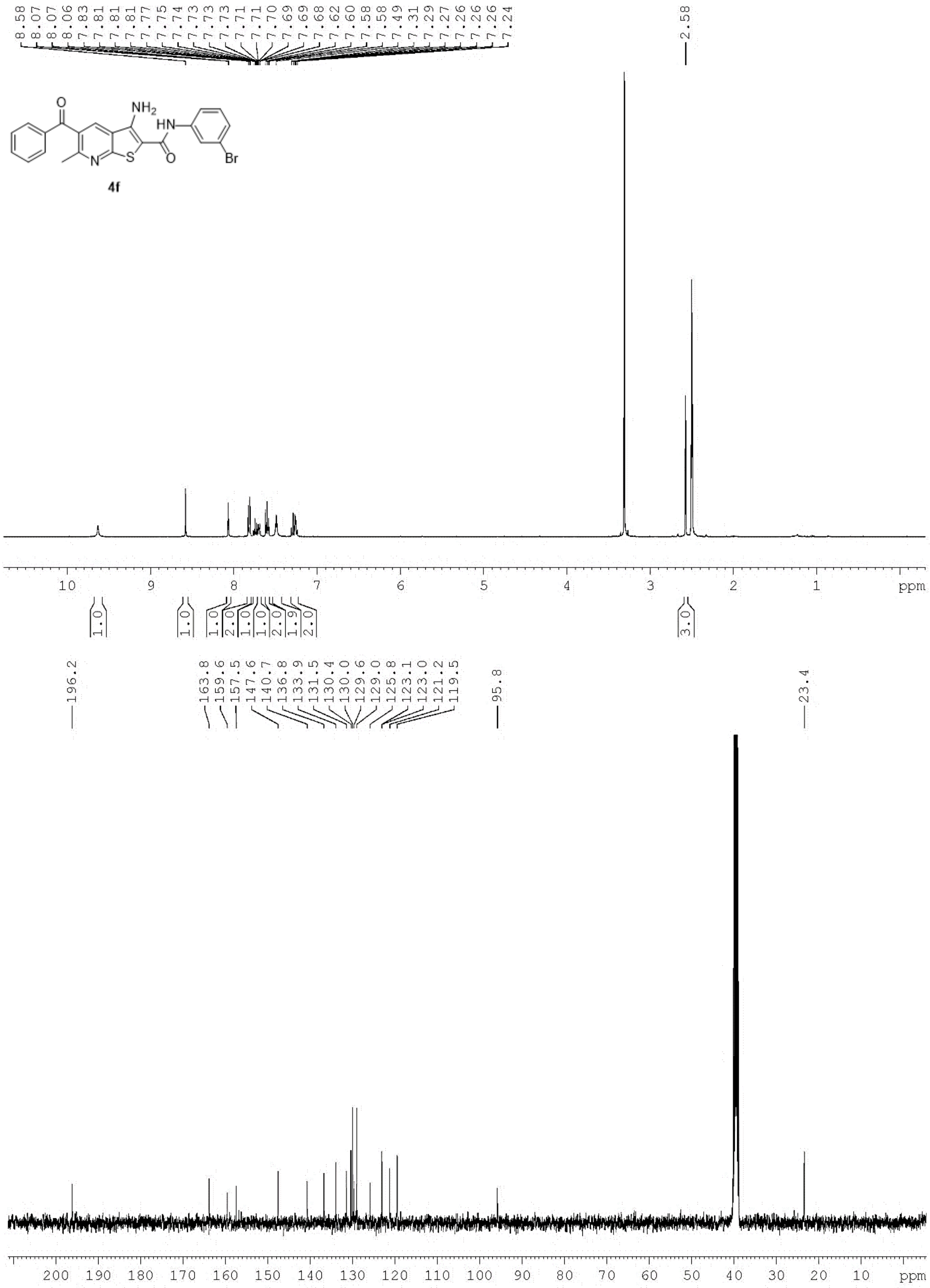

Figure S6: ^{1}H NMR and ^{13}C NMR spectra for **4f**.

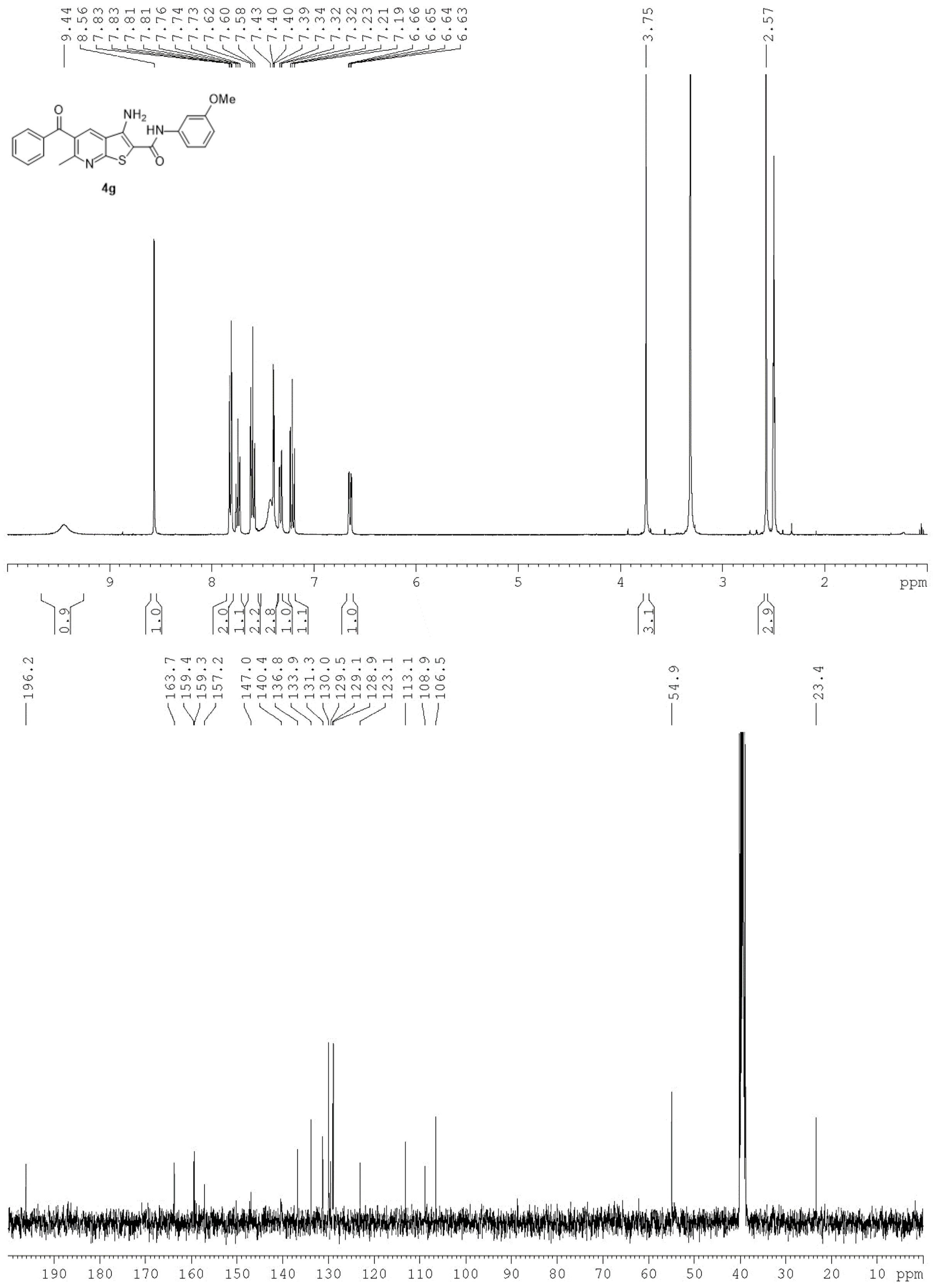

Figure S7: ^{1}H NMR and ^{13}C NMR spectra for **4g**.

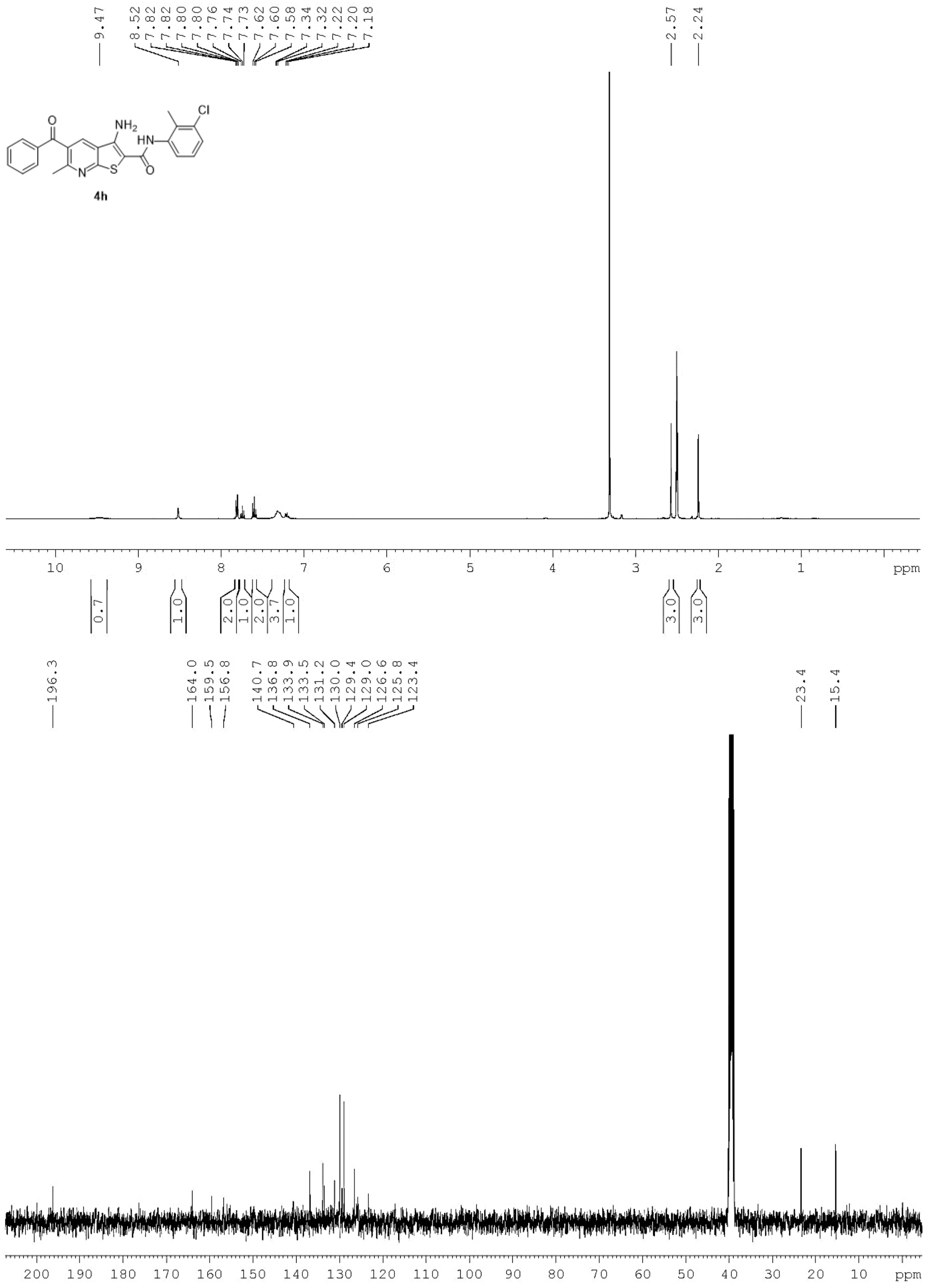

Figure S8: ^{1}H NMR and ^{13}C NMR spectra for **4h**.

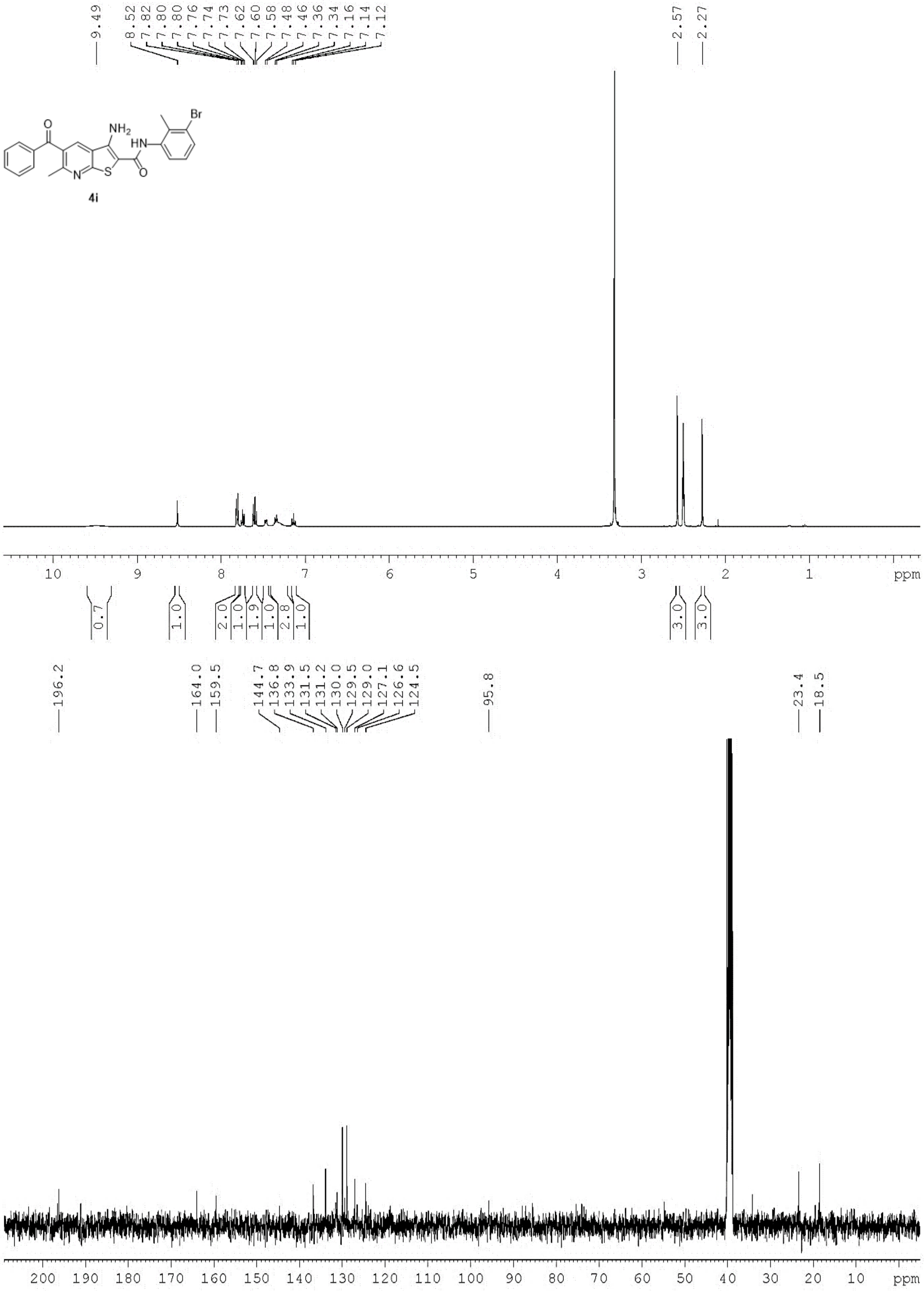

Figure S9: ^{1}H NMR and ^{13}C NMR spectra for **4i**.

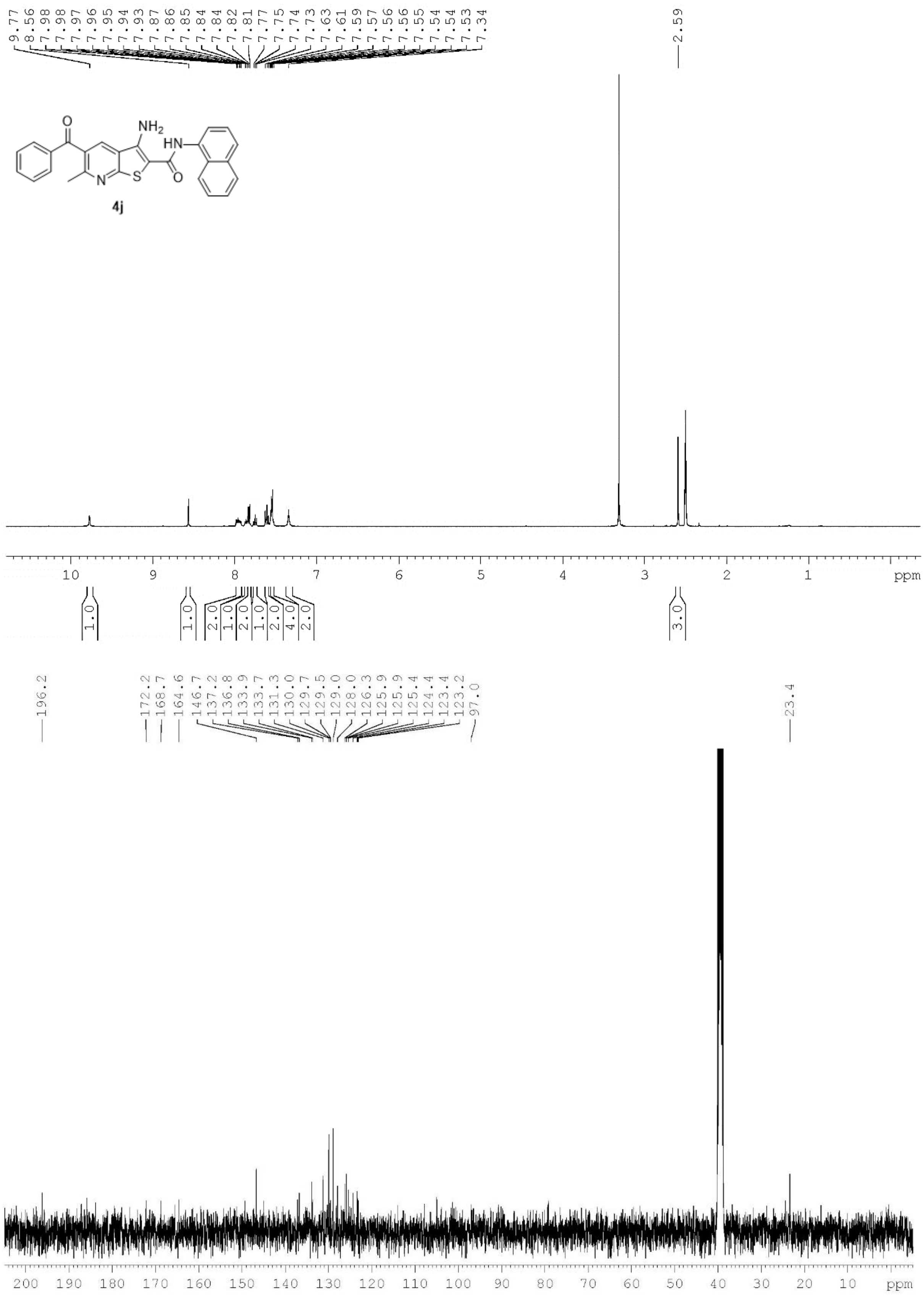

Figure S10: ^{1}H NMR and ^{13}C NMR spectra for **4j**.

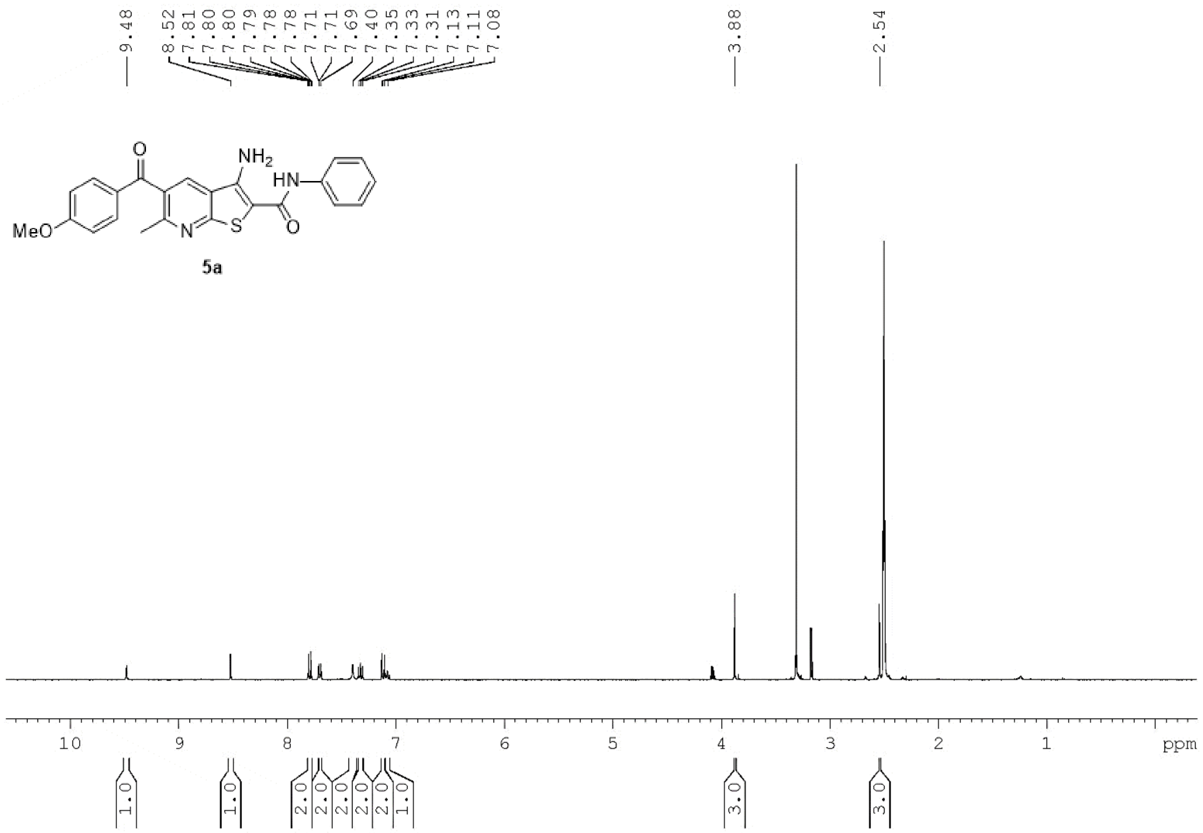

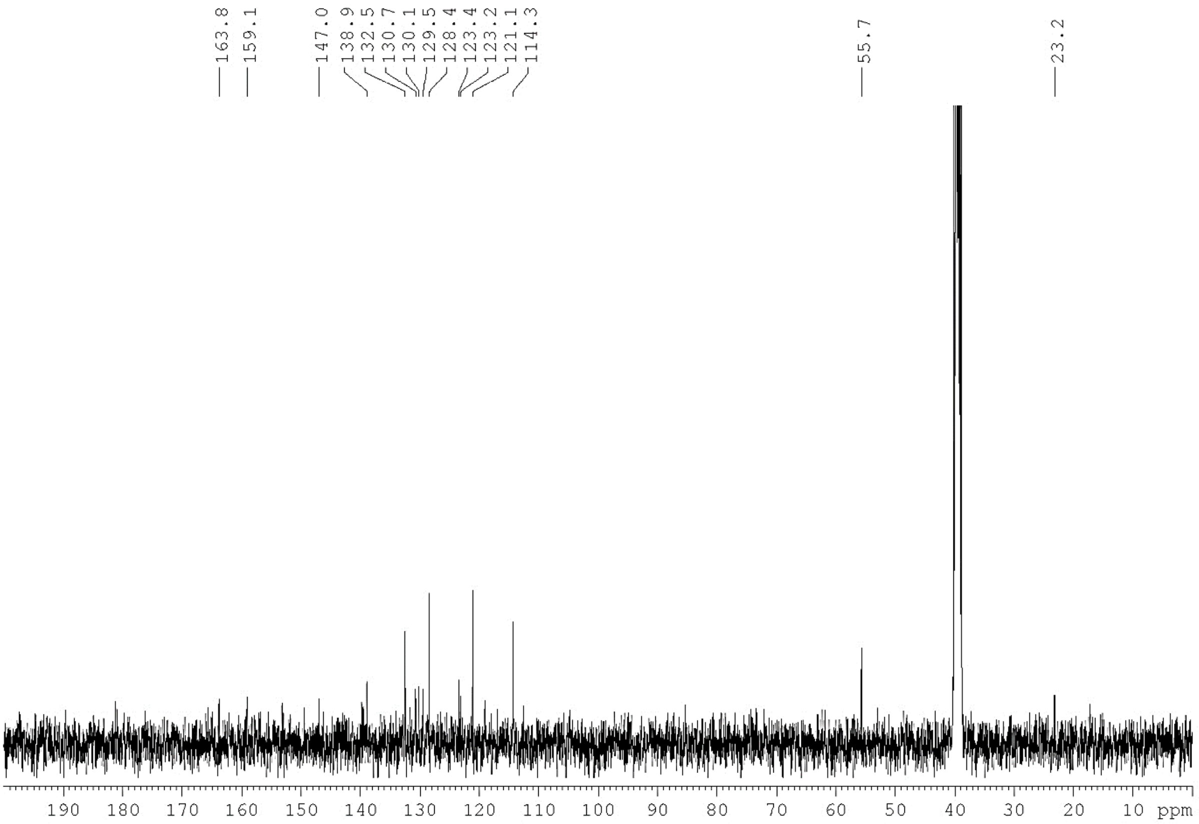

Figure S11: ^{1}H NMR and ^{13}C NMR spectra for **5a**.

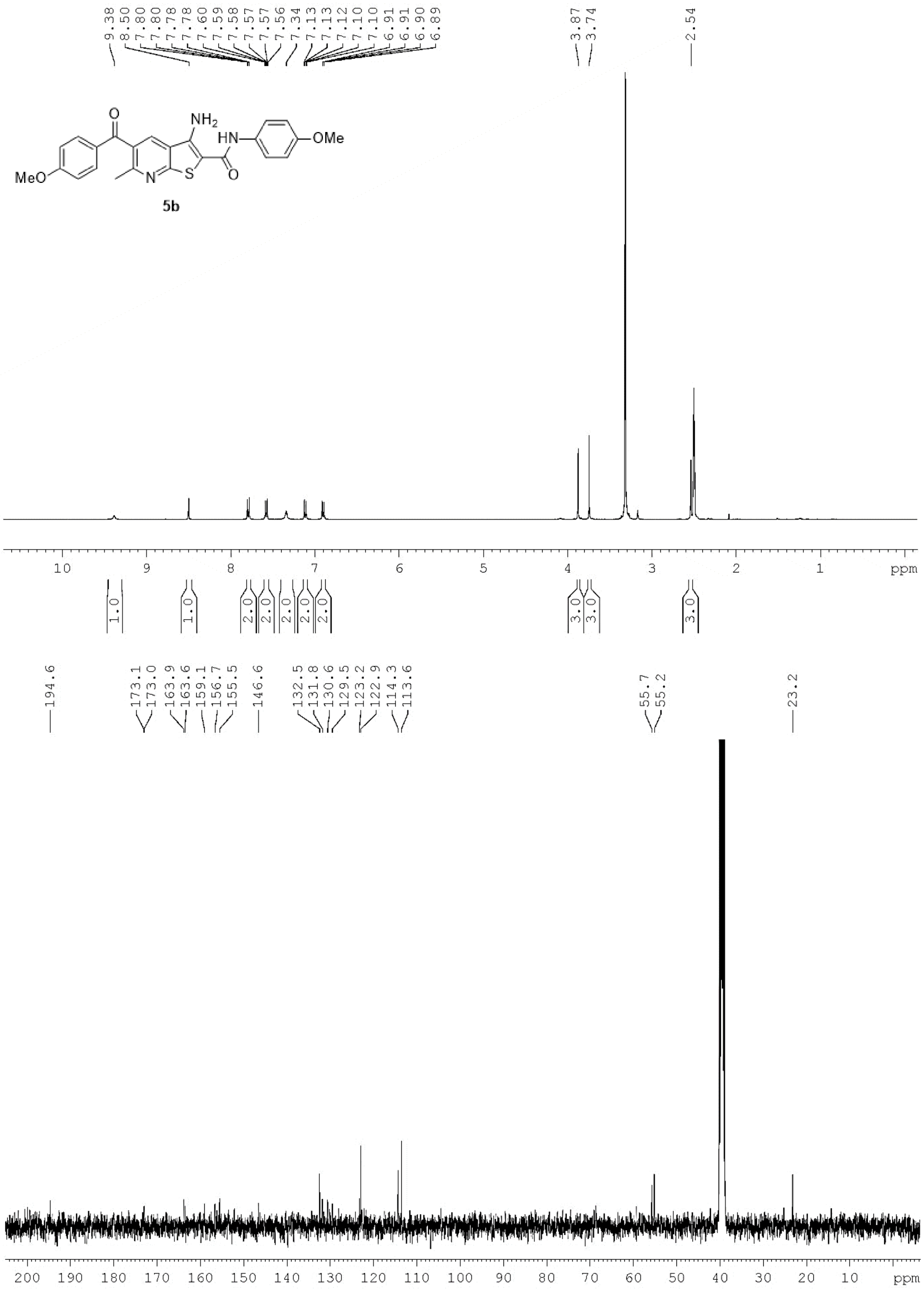

Figure S12: ^{1}H NMR and ^{13}C NMR spectra for **5b**.

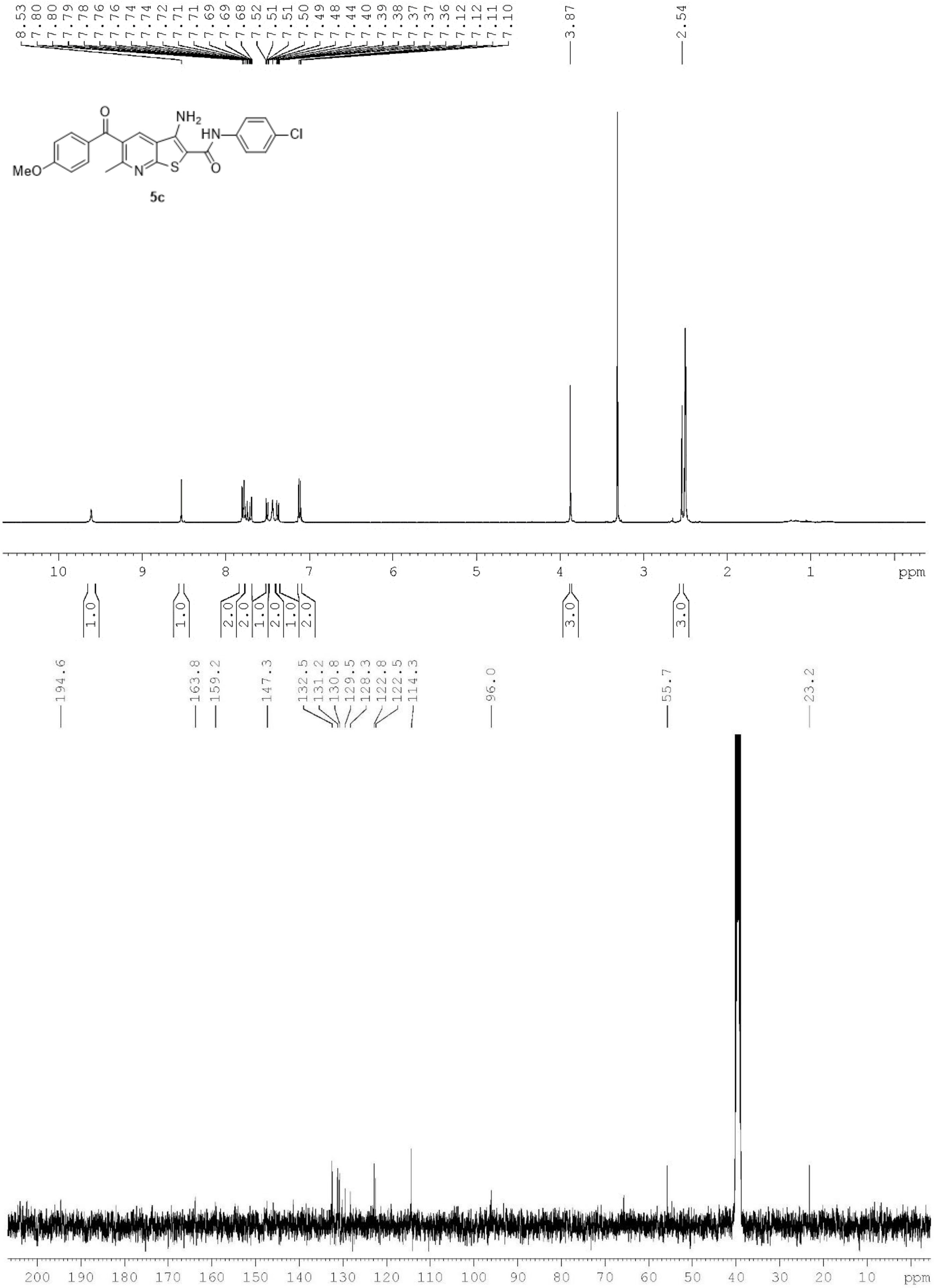

Figure S13: ^{1}H NMR and ^{13}C NMR spectra for **5c**.

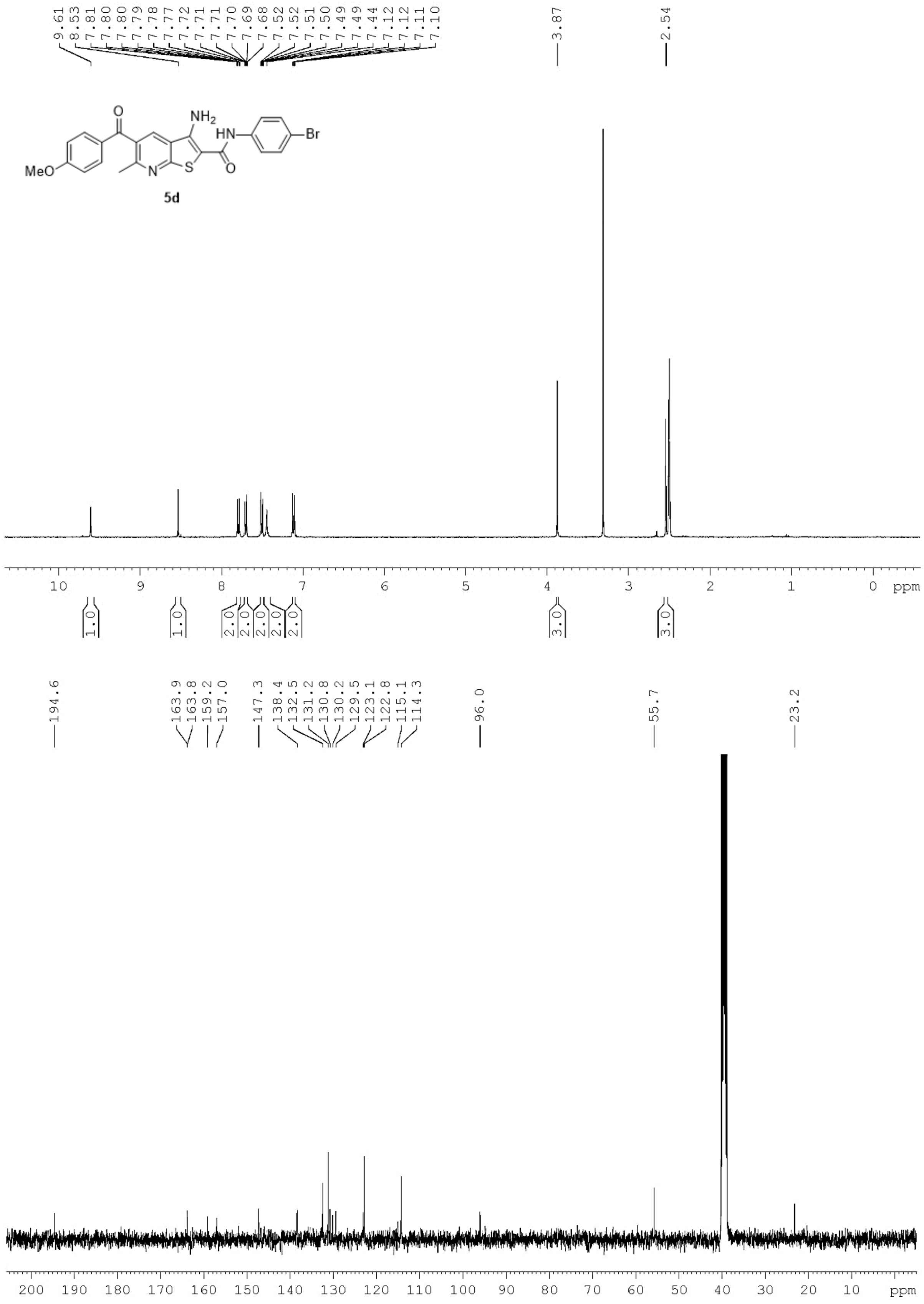

Figure S14: ^{1}H NMR and ^{13}C NMR spectra for **5d**.

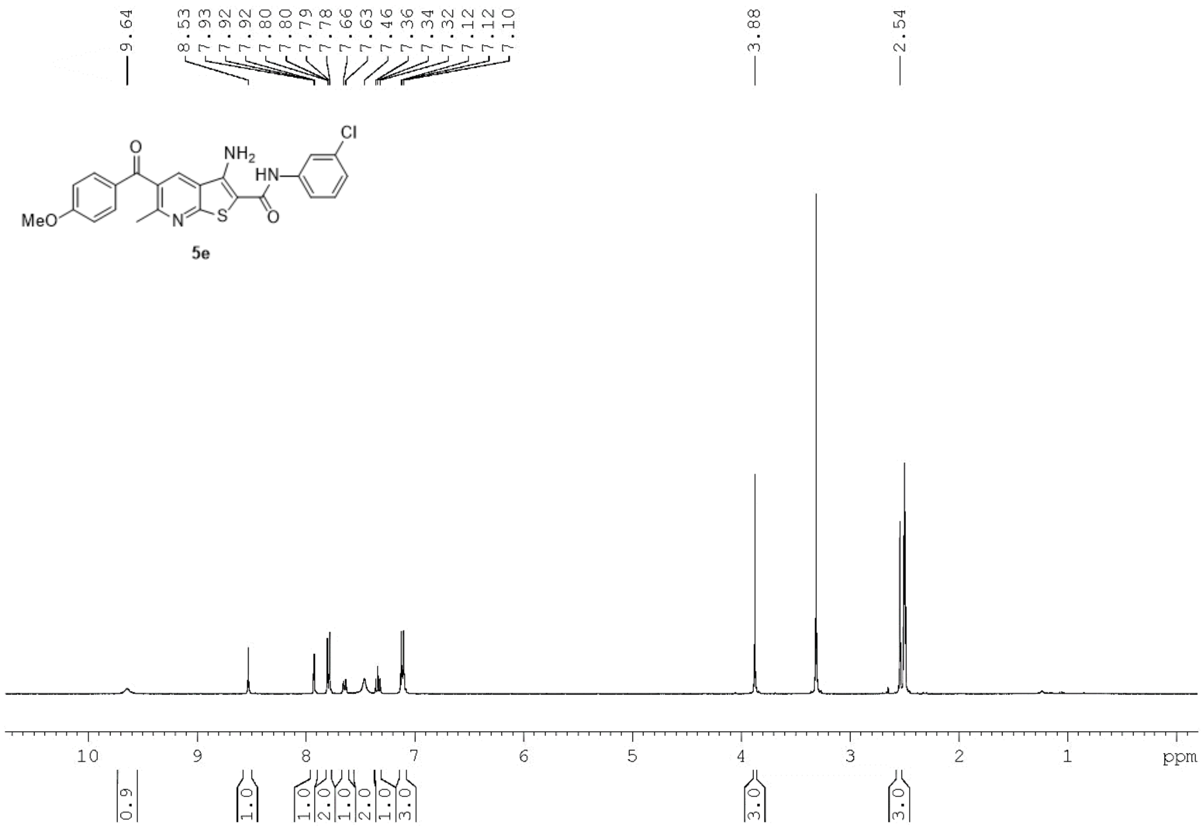

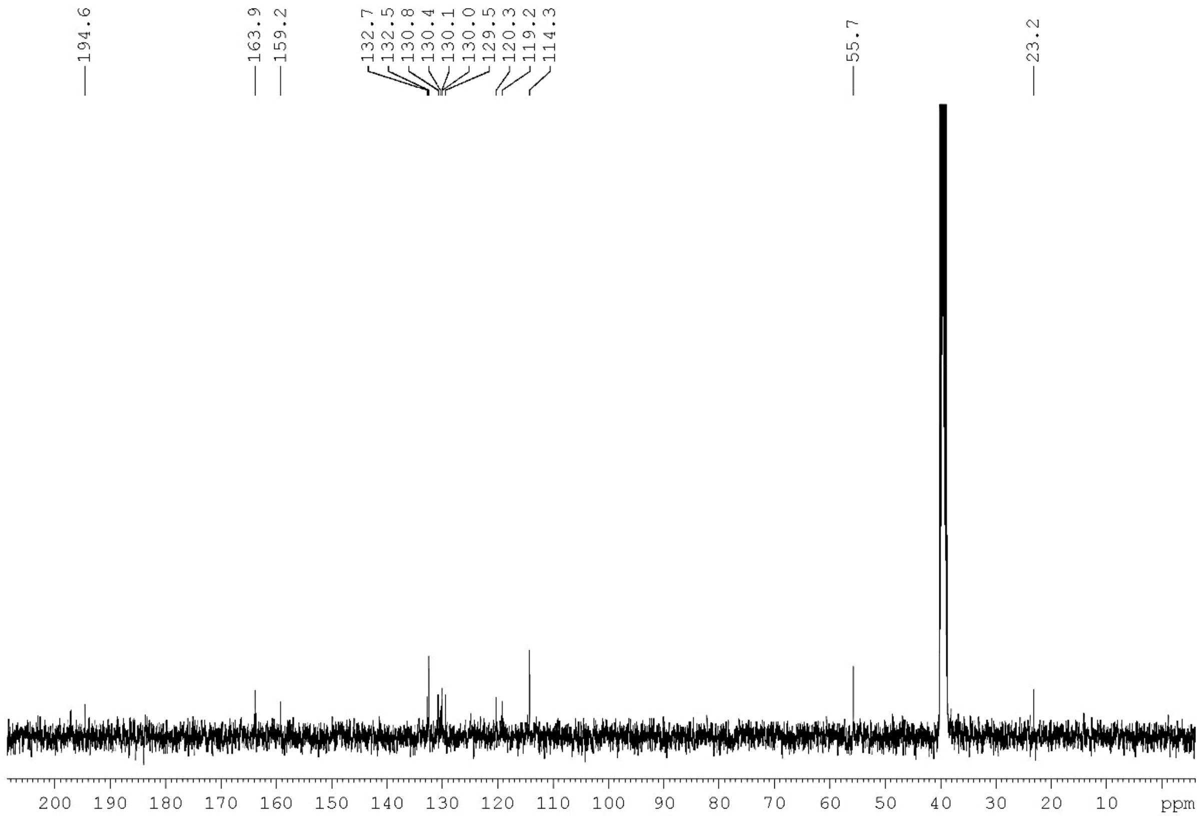

Figure S15: ^{1}H NMR and ^{13}C NMR spectra for **5e**.

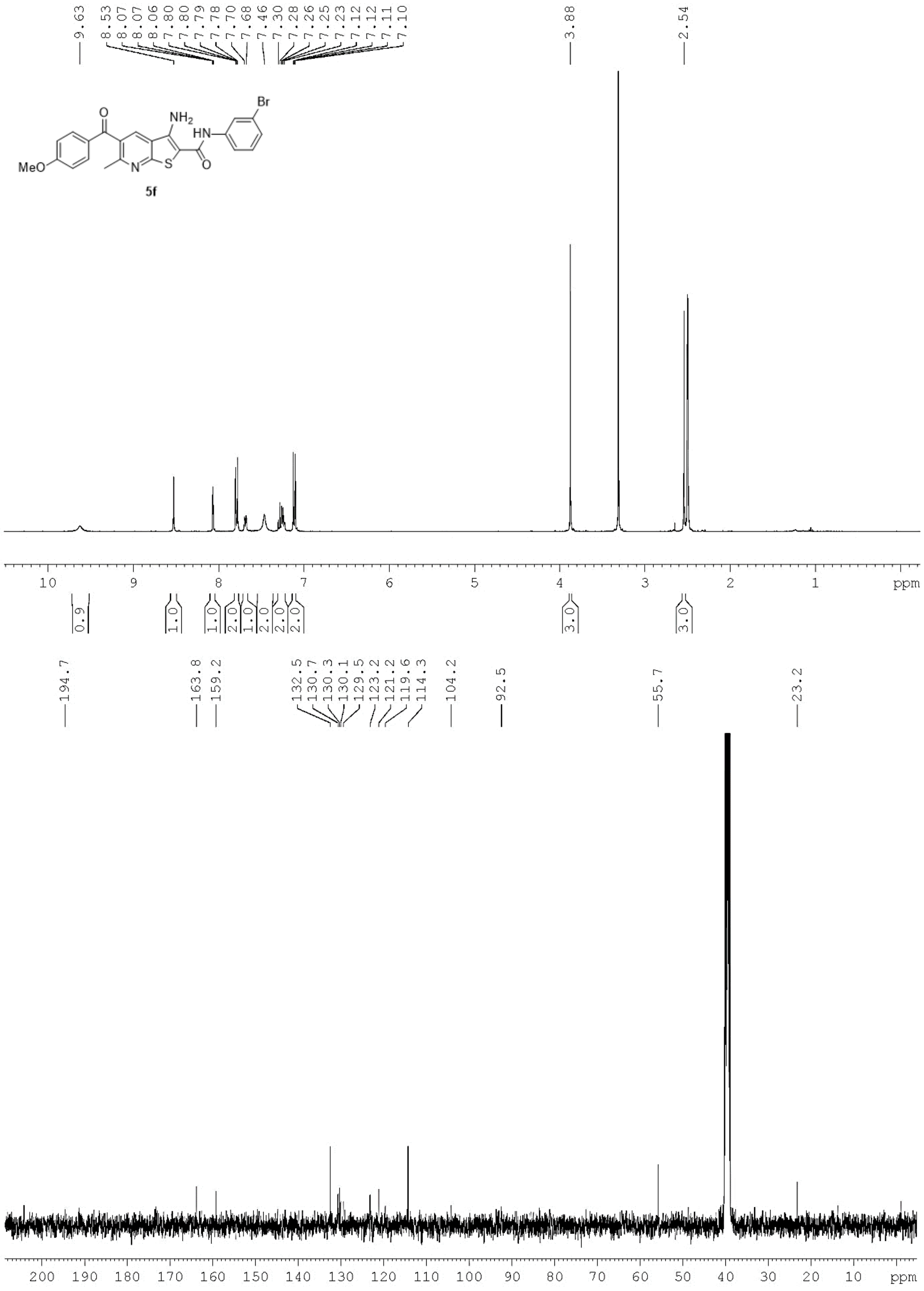

Figure S16: ^{1}H NMR and ^{13}C NMR spectra for **5f**.

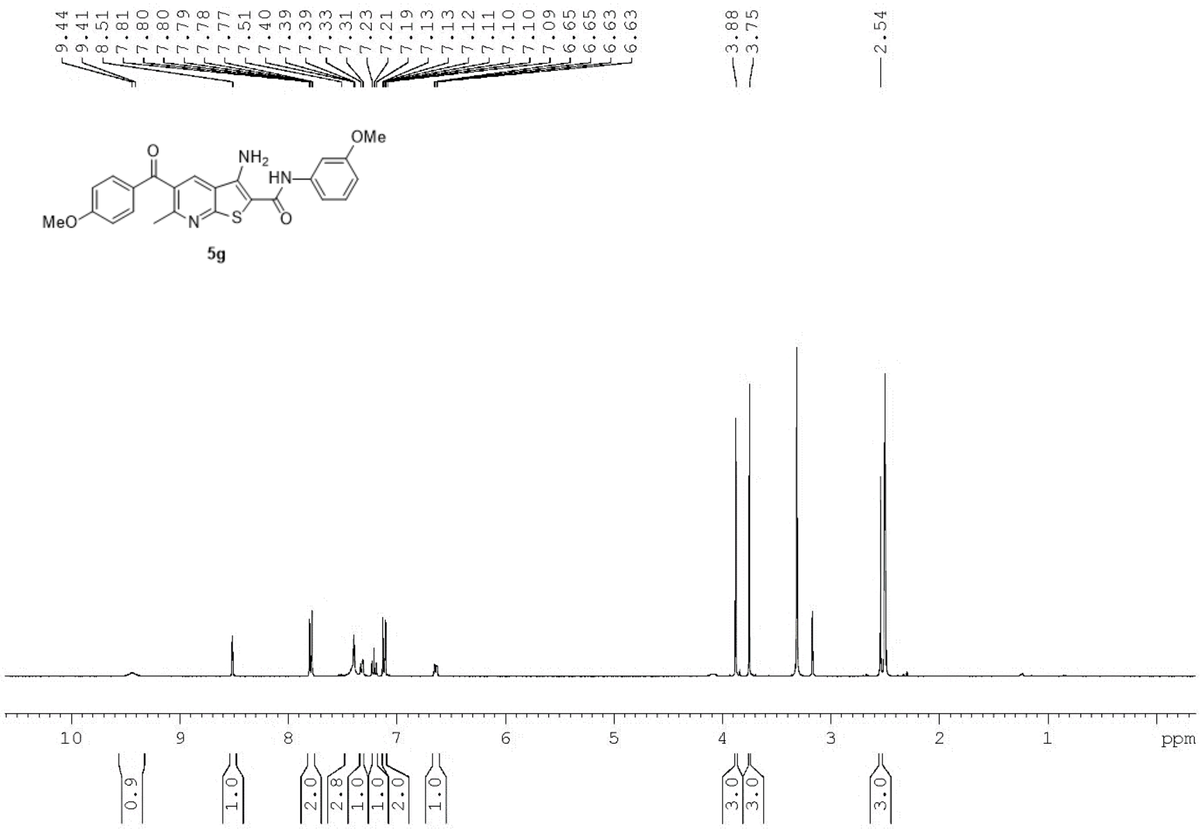

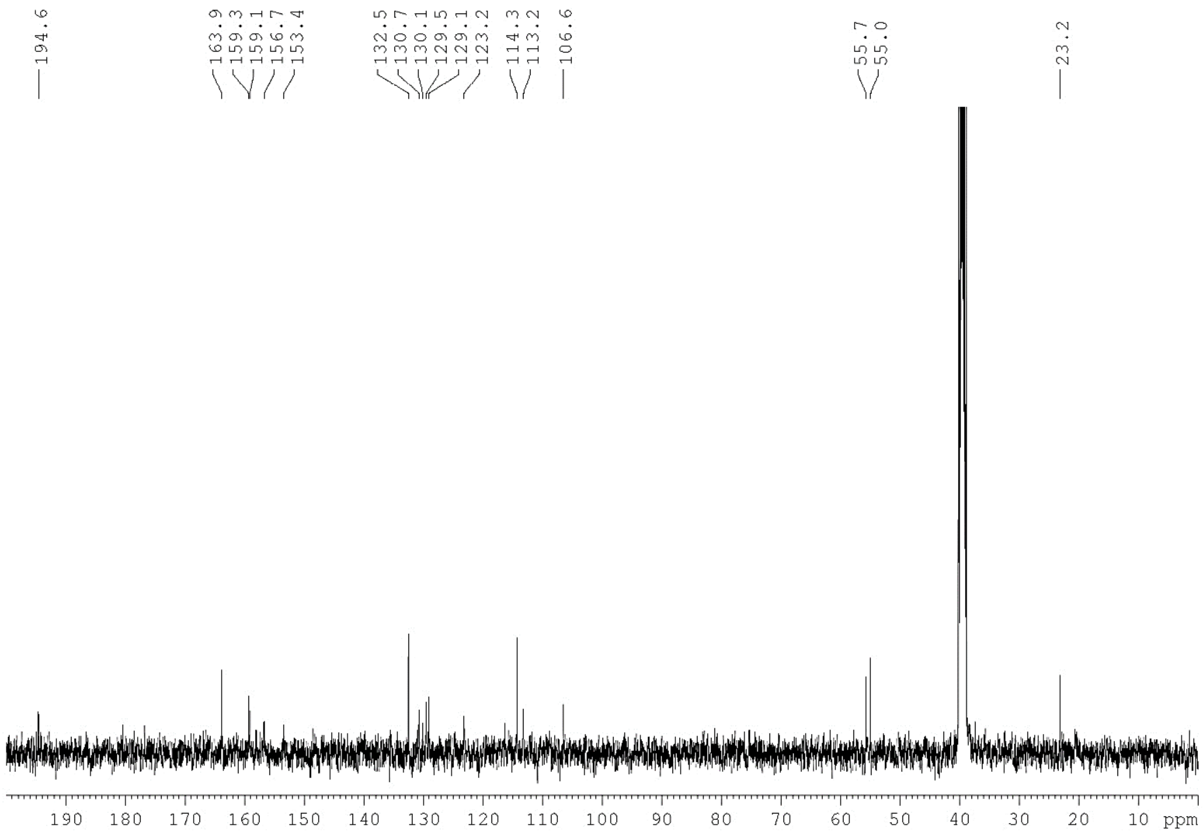

Figure S17: ^{1}H NMR and ^{13}C NMR spectra for **5g**.

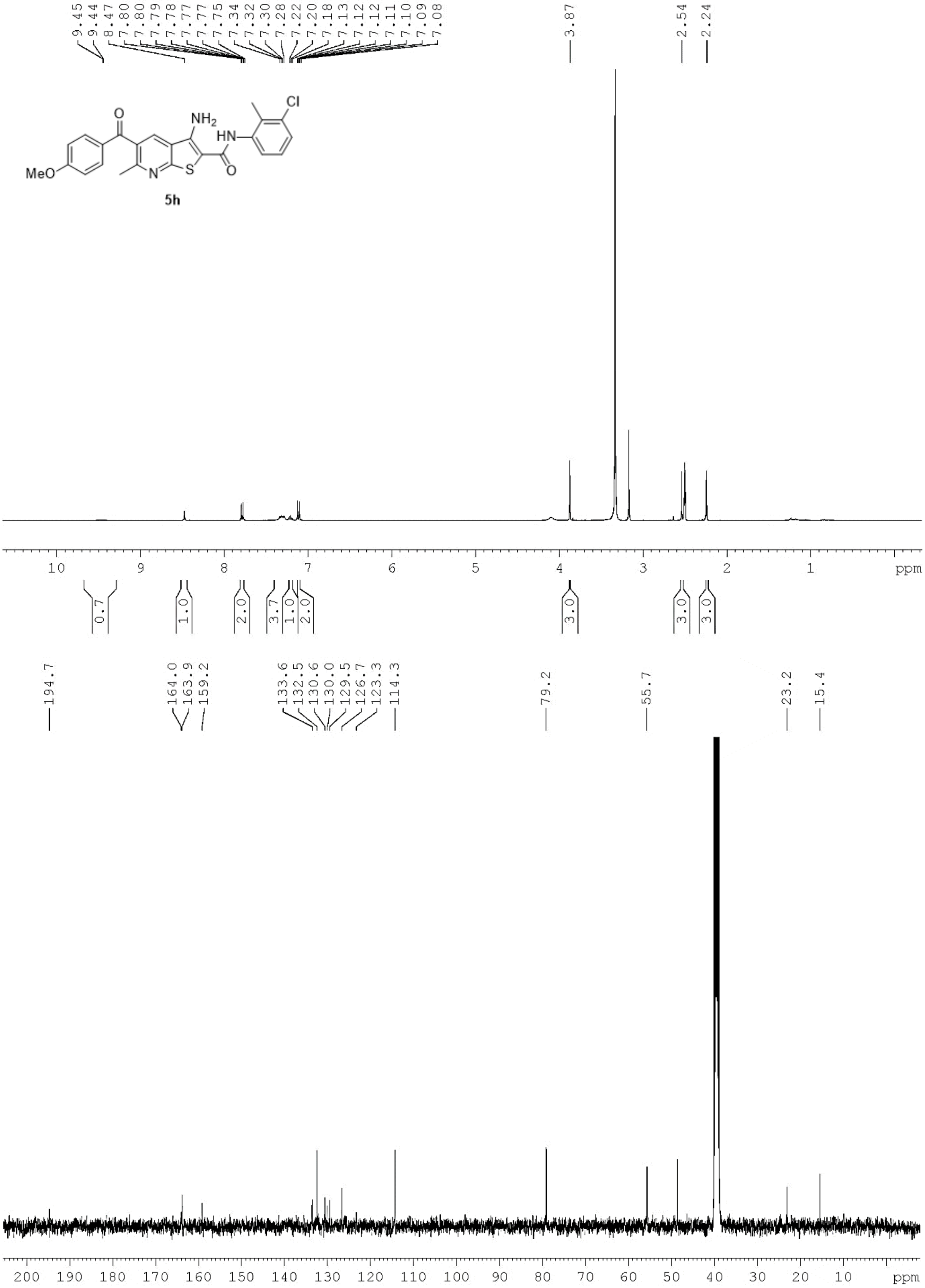

Figure S18: ^{1}H NMR and ^{13}C NMR spectra for **5h**.

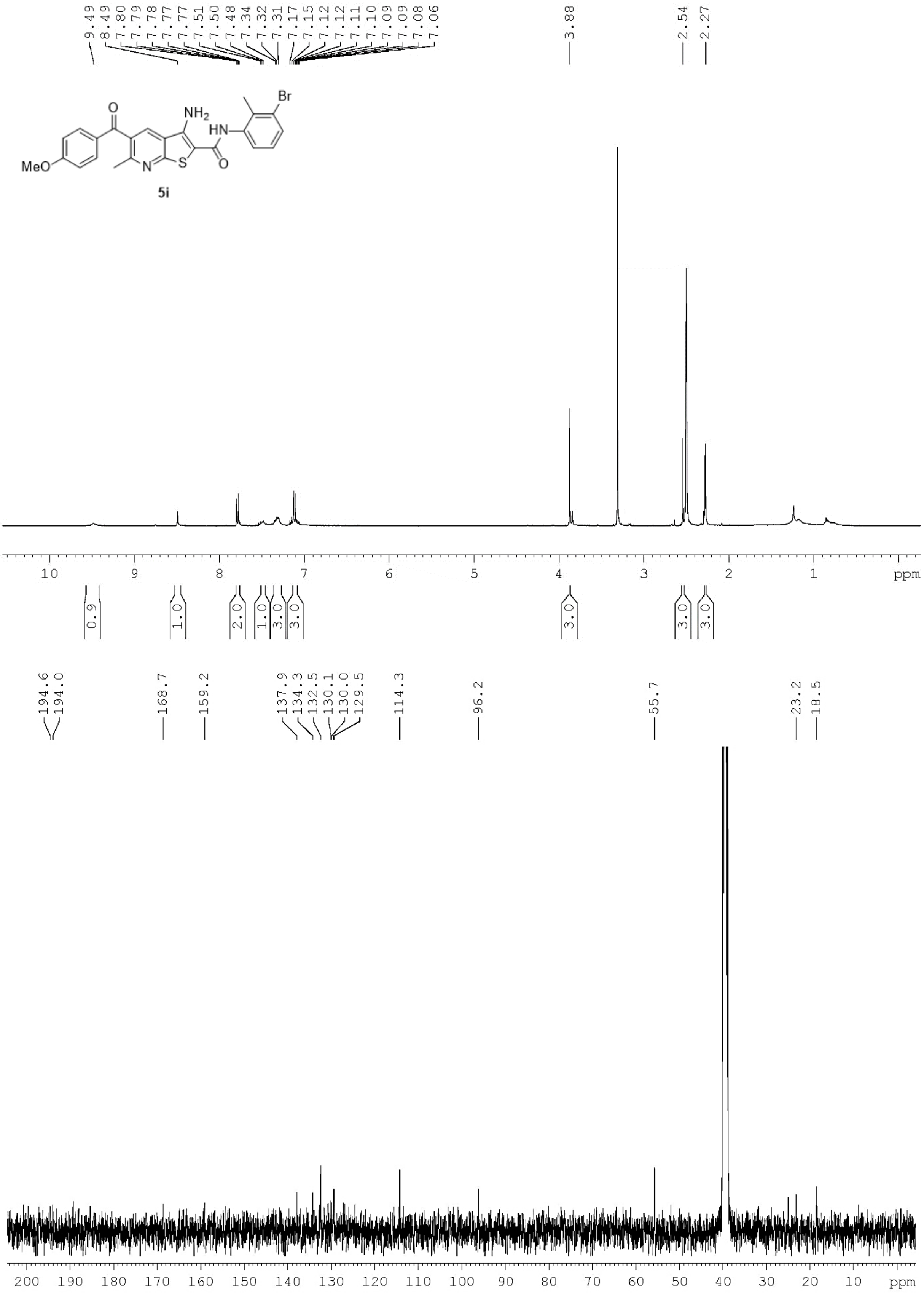

Figure S19: ^{1}H NMR and ^{13}C NMR spectra for **5i**.

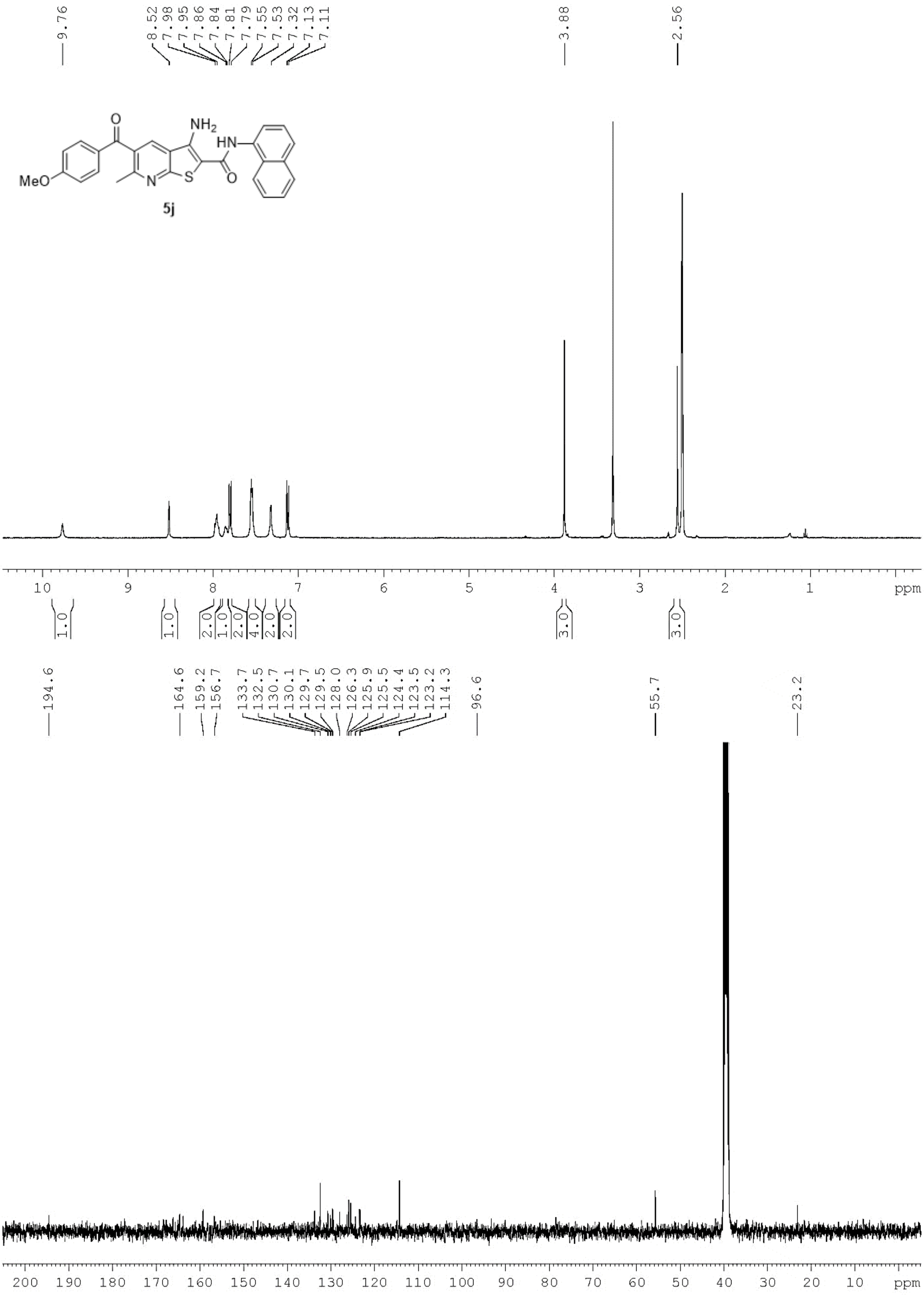

Figure S20: ^{1}H NMR and ^{13}C NMR spectra for **5j**.

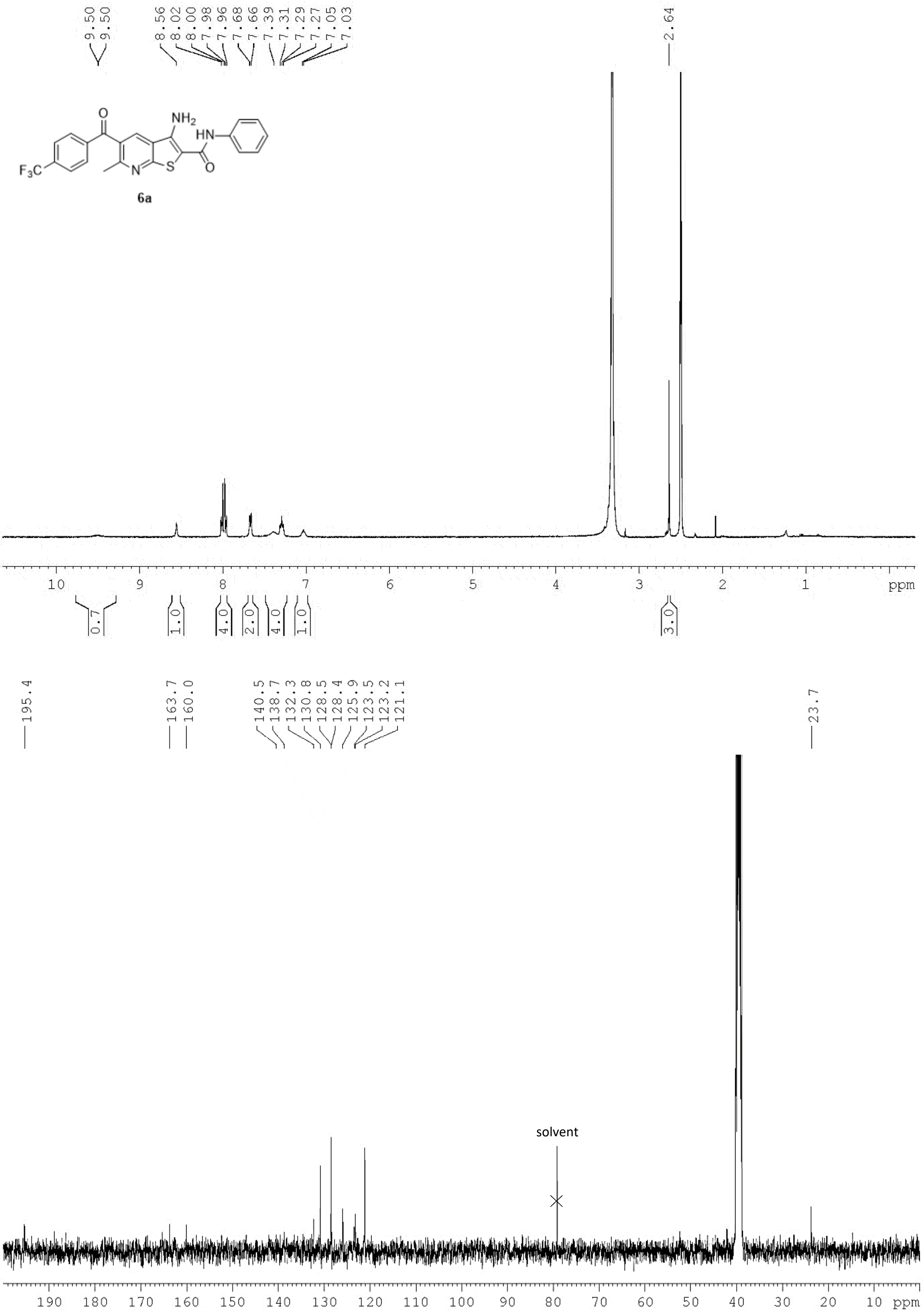

Figure S21: ^{1}H NMR and ^{13}C NMR spectra for **6a**.

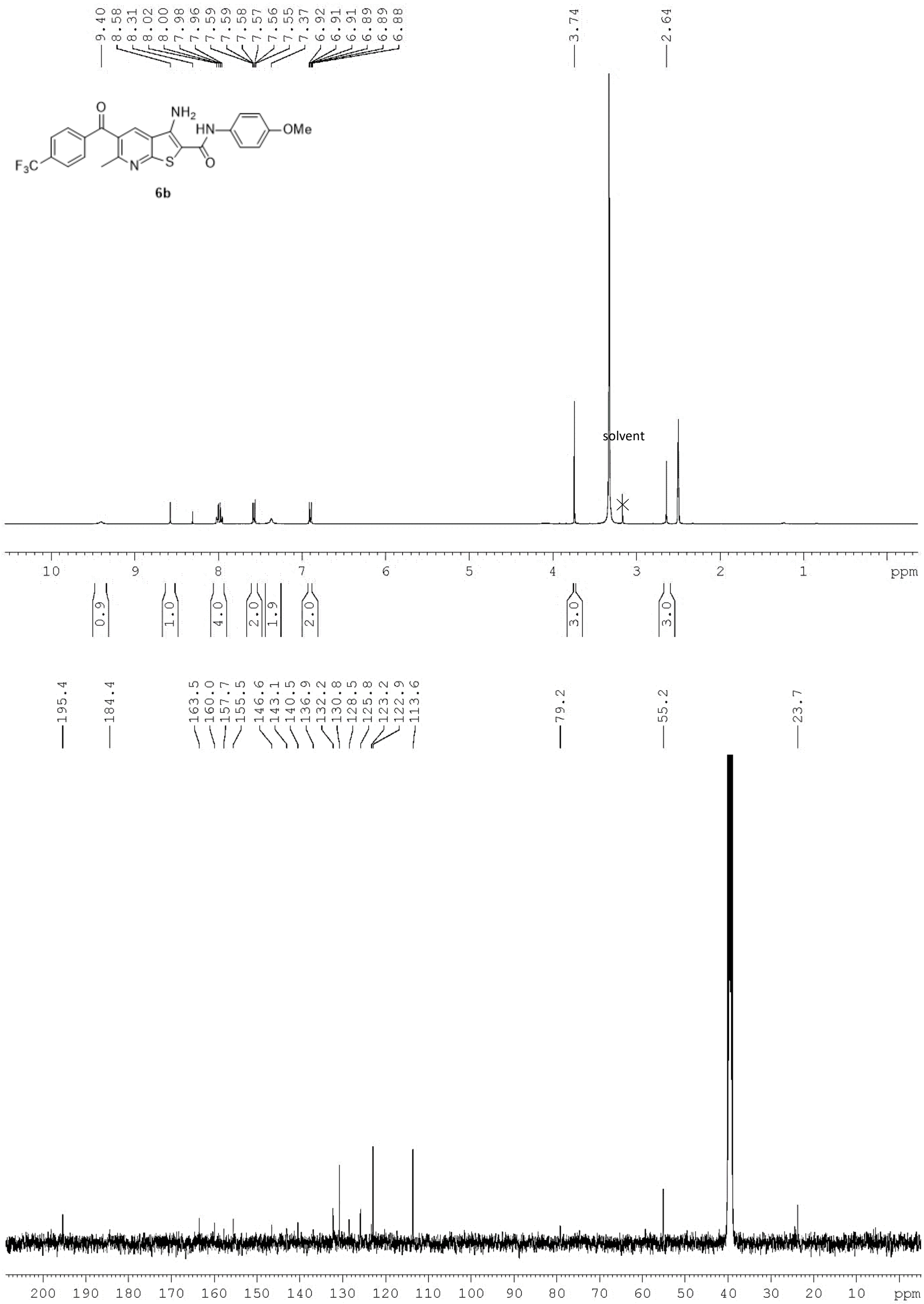

Figure S22: ^{1}H NMR and ^{13}C NMR spectra for **6b**.

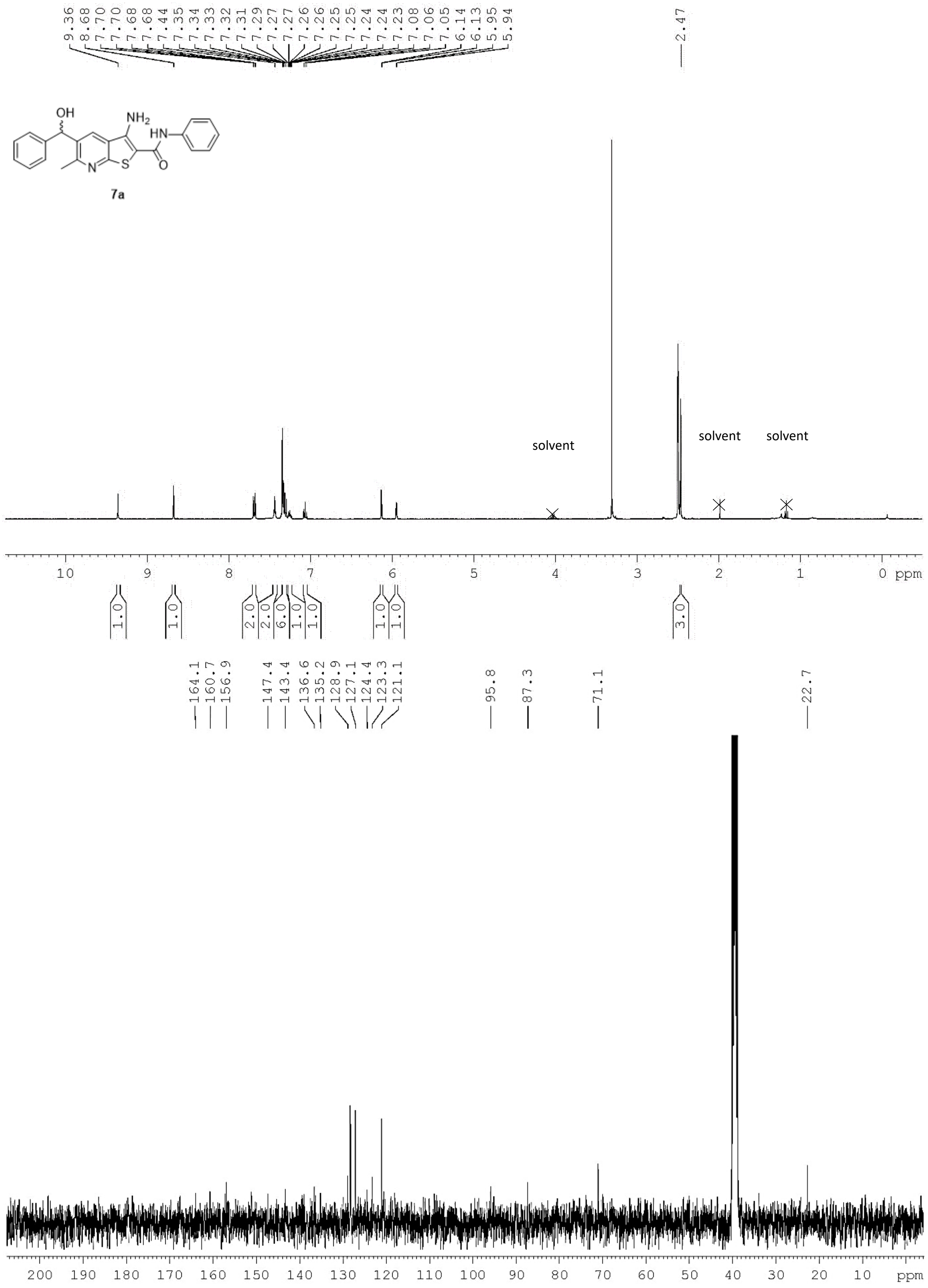

Figure S23: ^{1}H NMR and ^{13}C NMR spectra for **7a**.

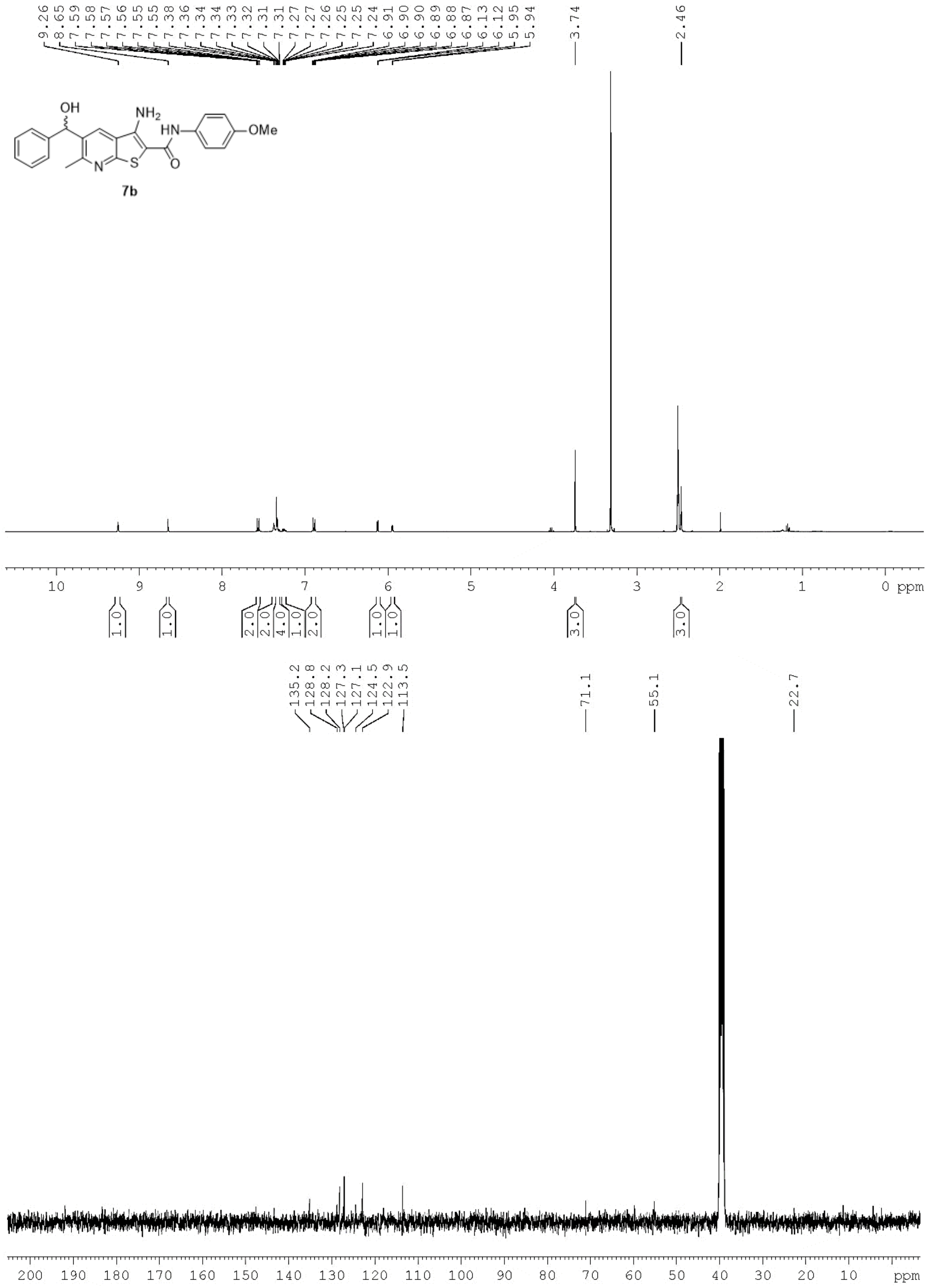

Figure S24: ^{1}H NMR and ^{13}C NMR spectra for **7b**.

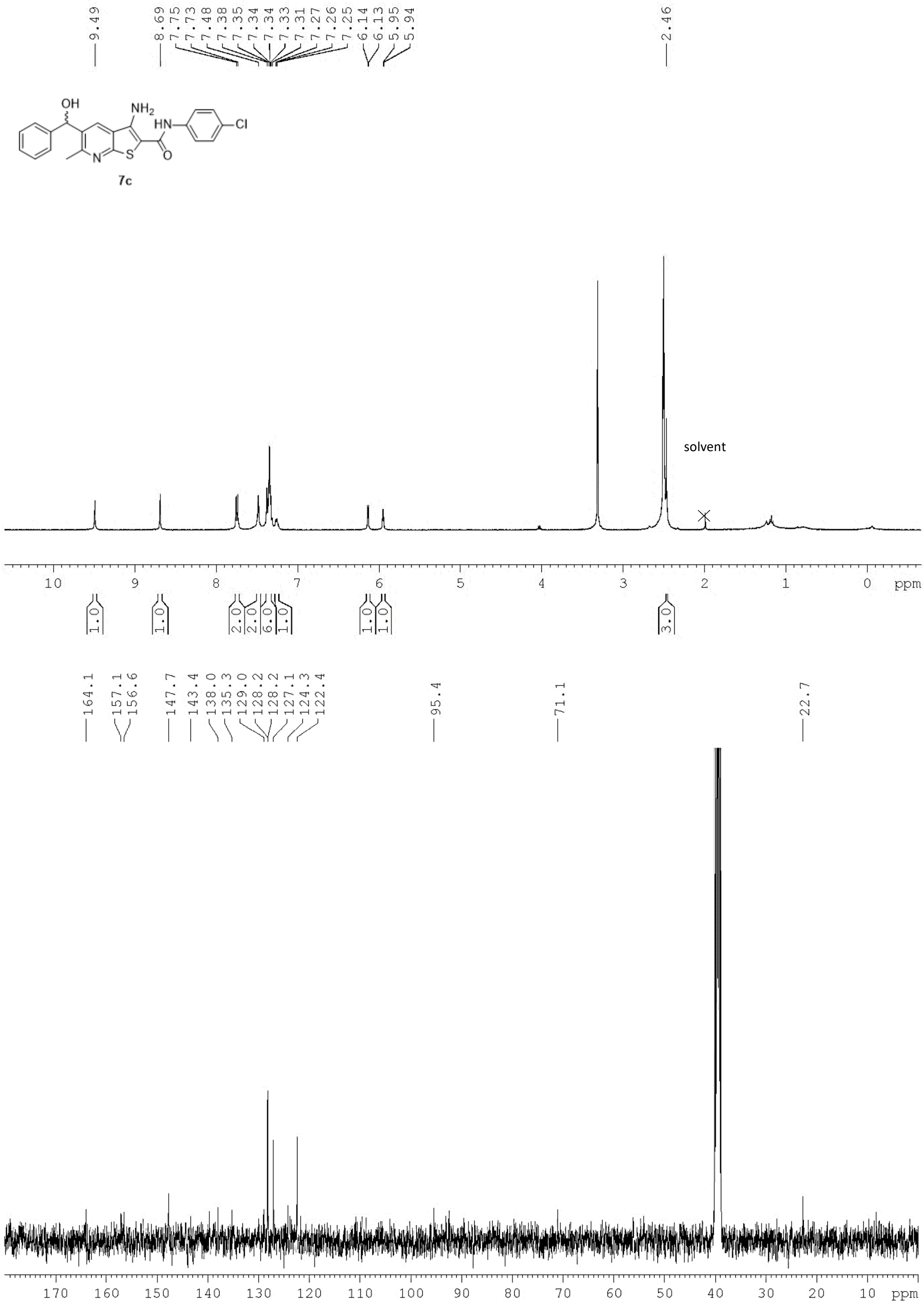

Figure S25: ^{1}H NMR and ^{13}C NMR spectra for **7c**.

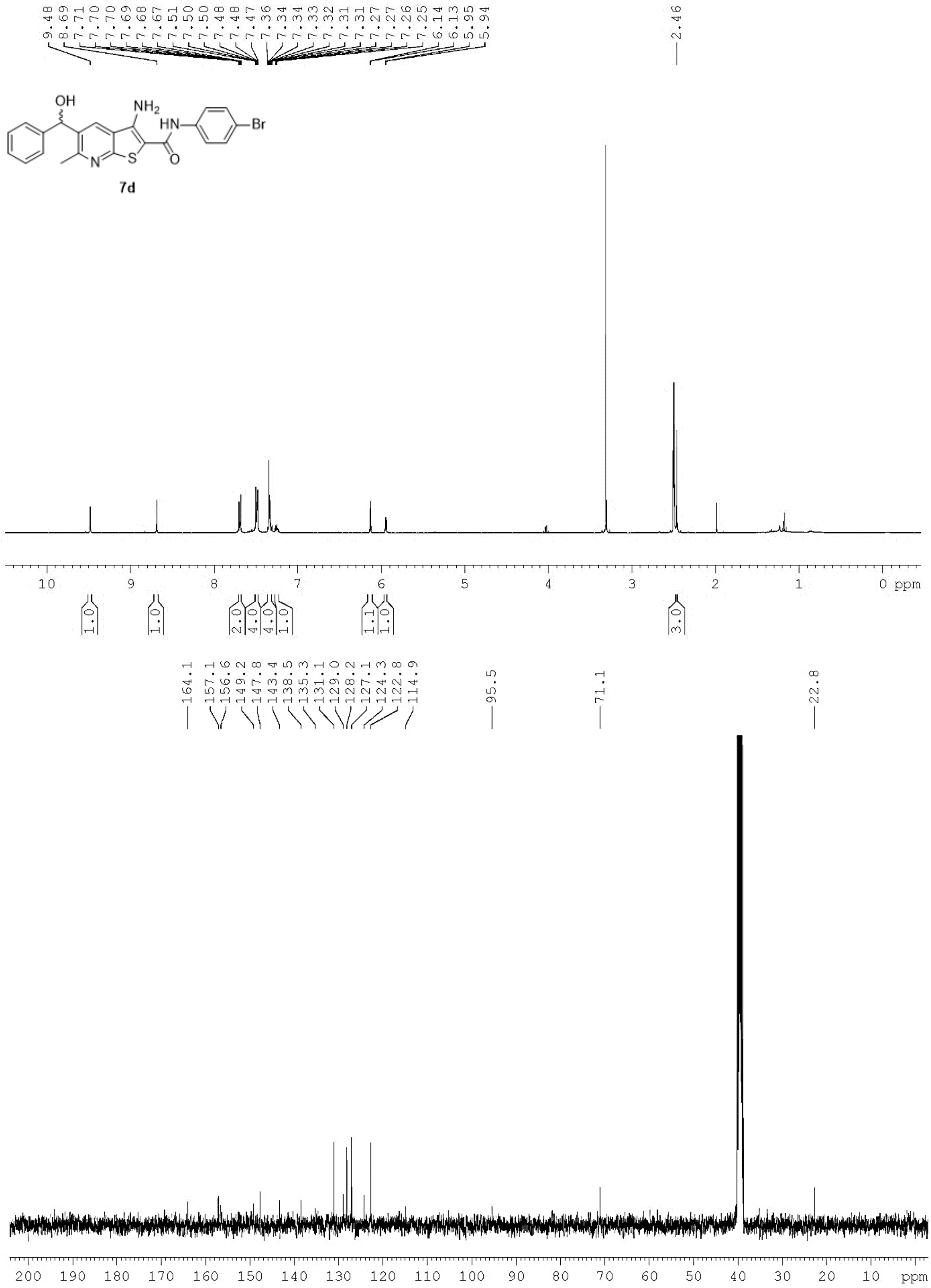

Figure S26: ^{1}H NMR and ^{13}C NMR spectra for **7d**.

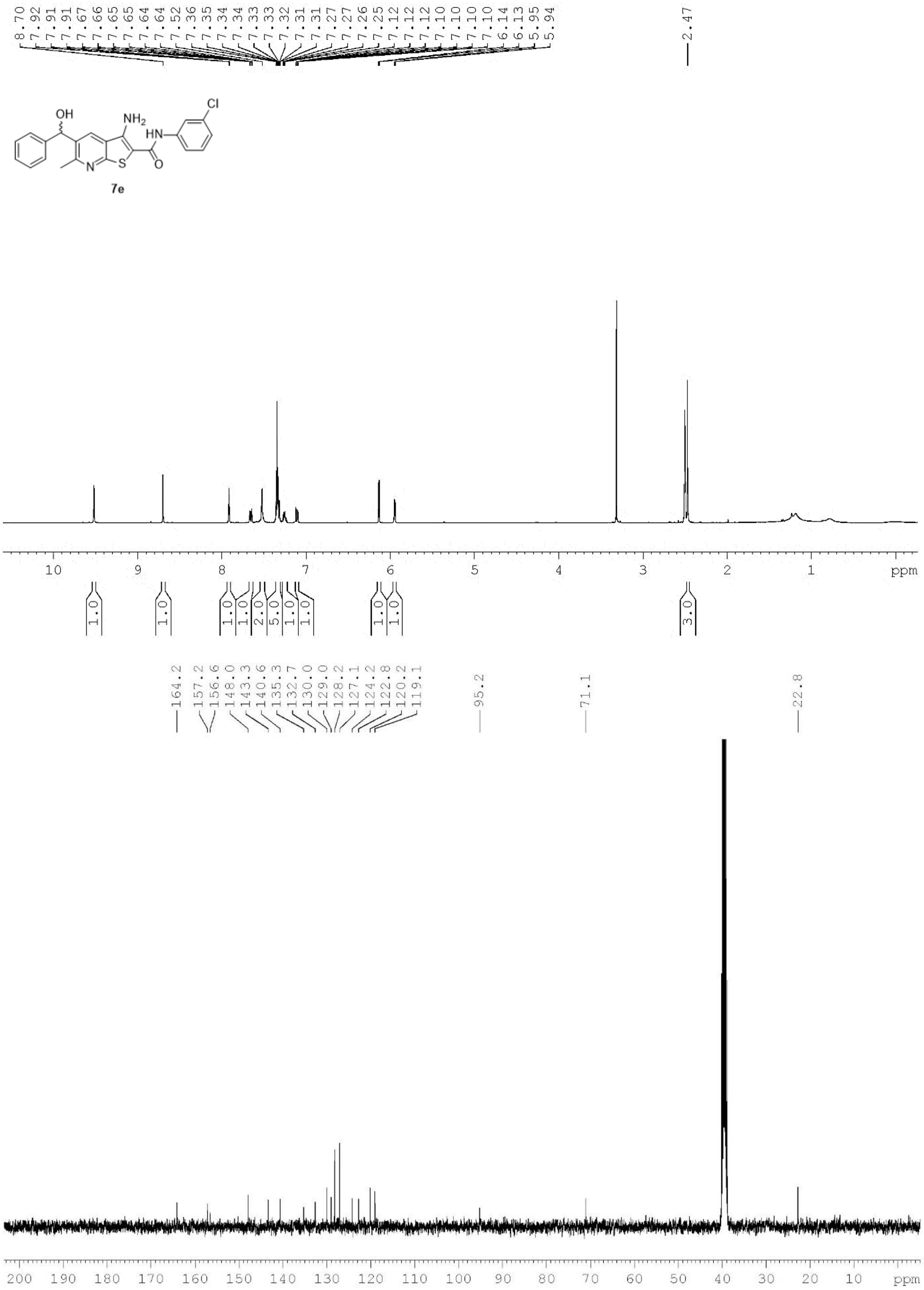

Figure S27: ^{1}H NMR and ^{13}C NMR spectra for **7e**.

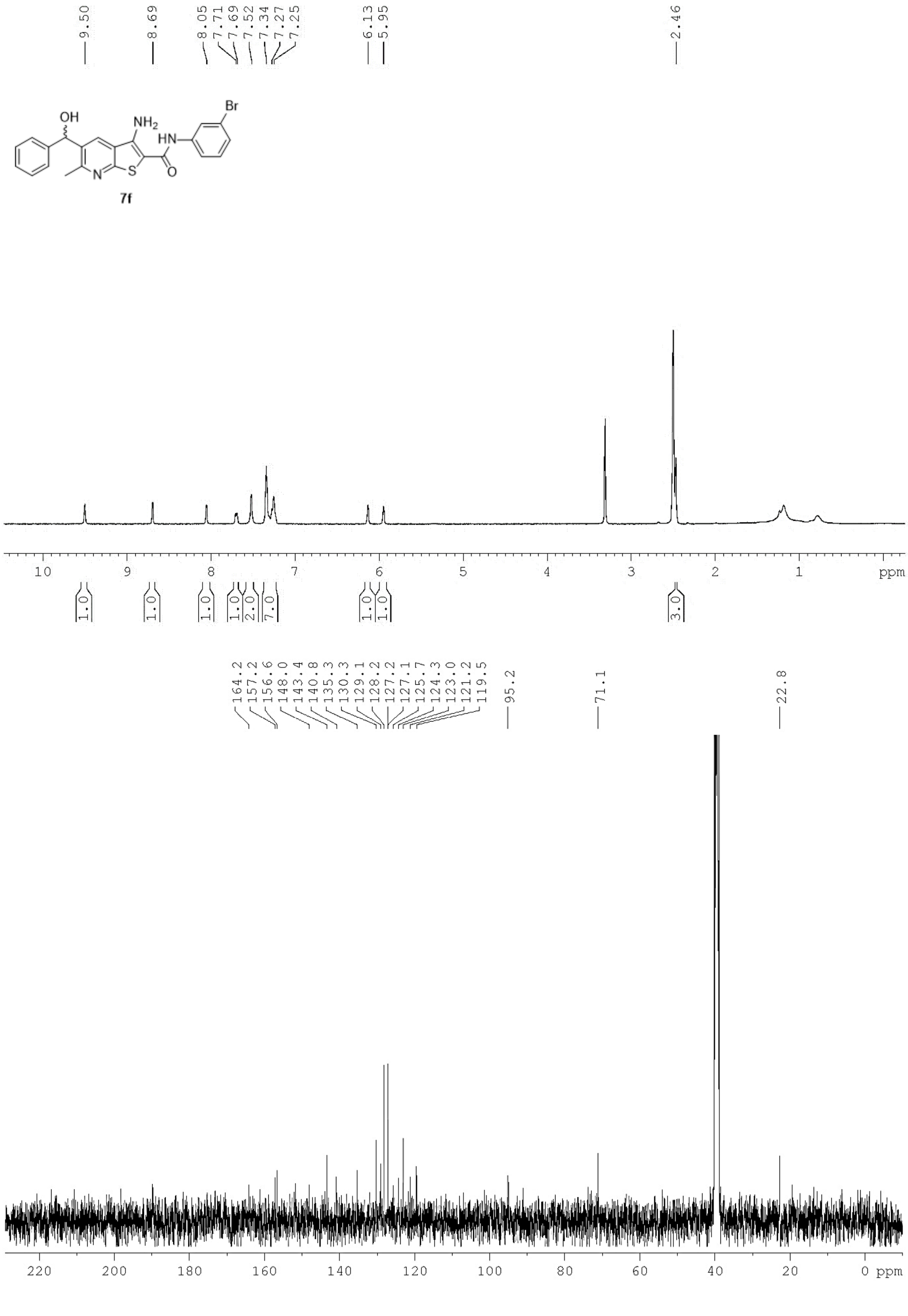

Figure S28: ^{1}H NMR and ^{13}C NMR spectra for **7f**.

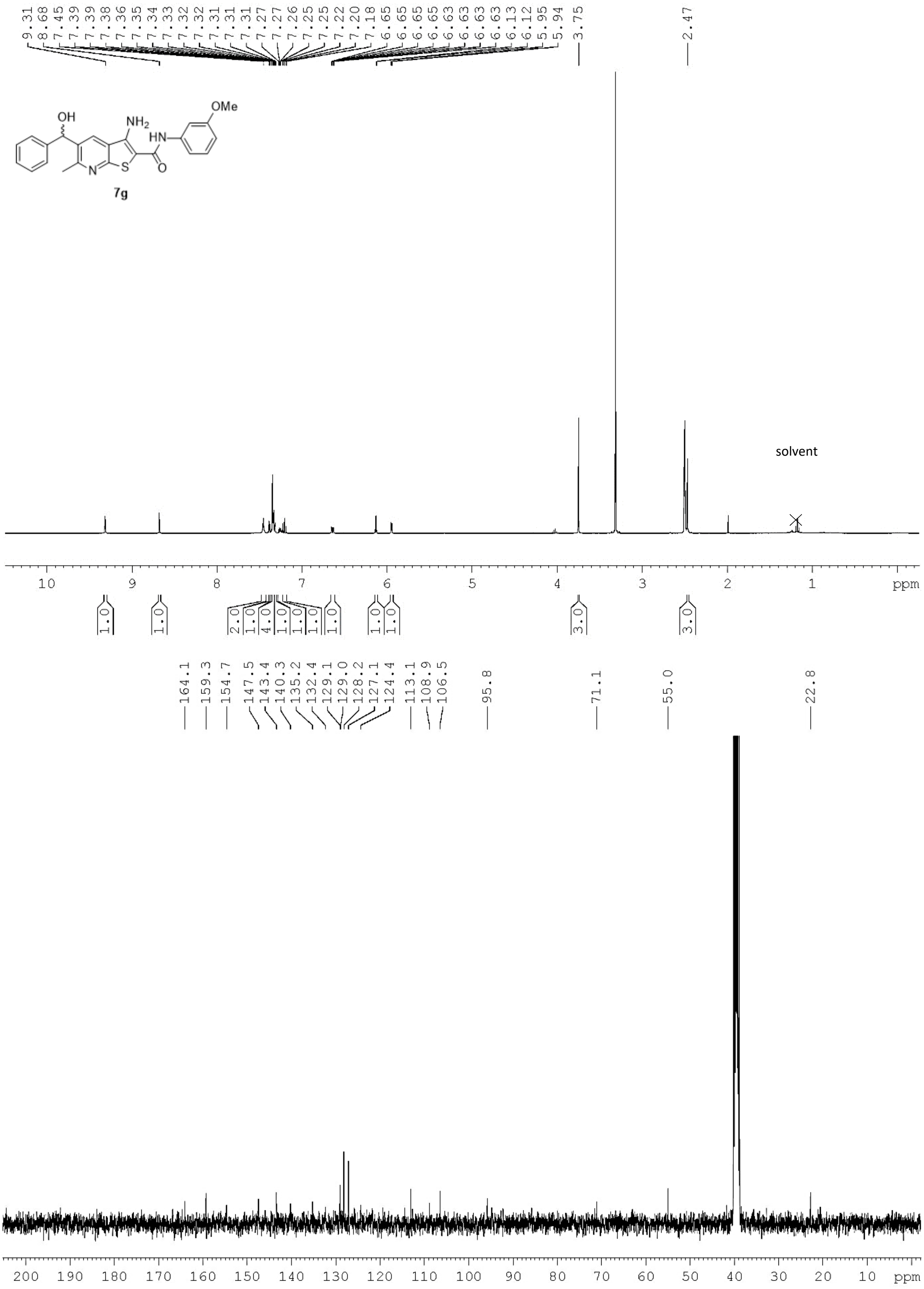

Figure S29: ^{1}H NMR and ^{13}C NMR spectra for **7g**.

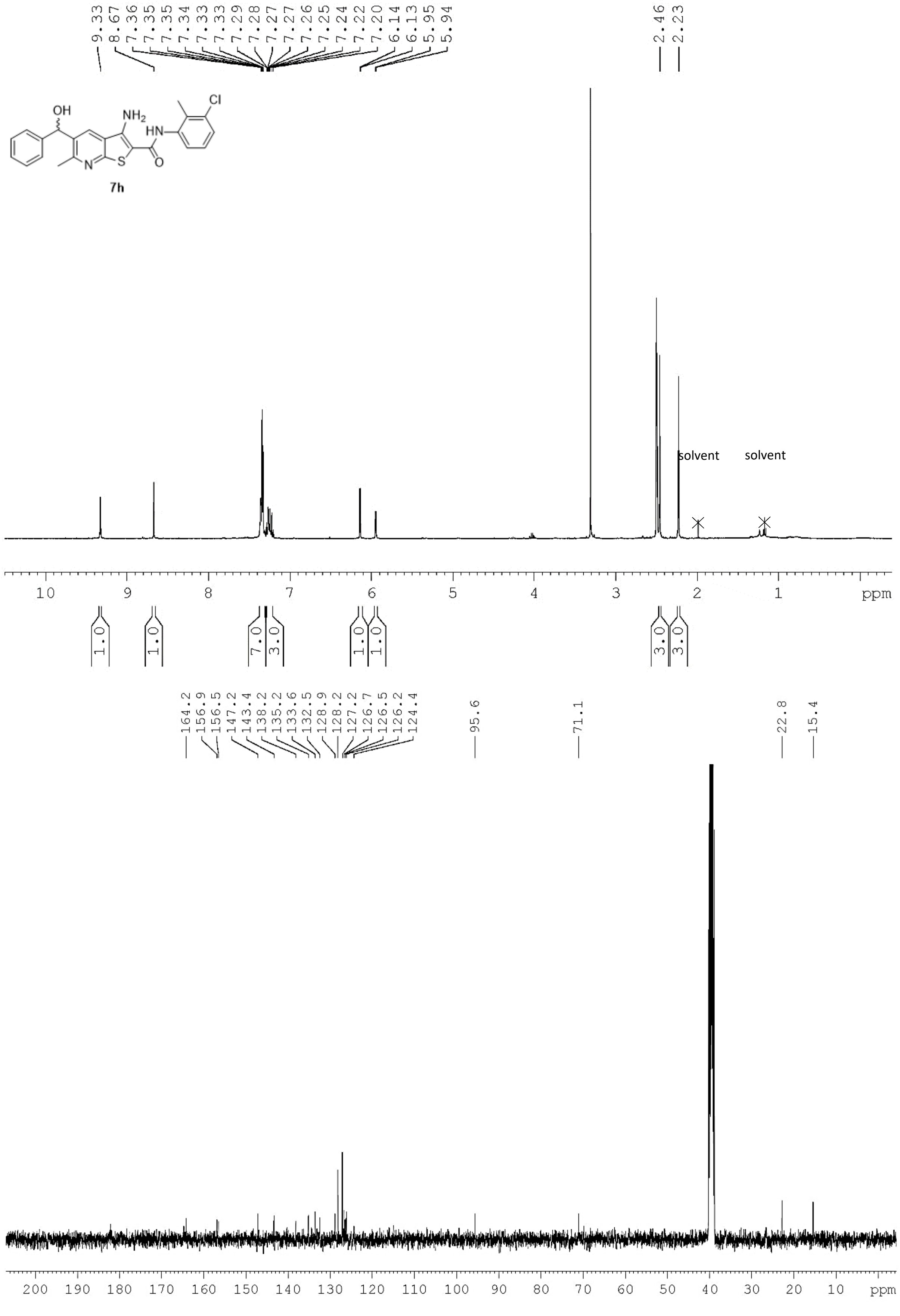

Figure S30: ^{1}H NMR and ^{13}C NMR spectra for **7h**.

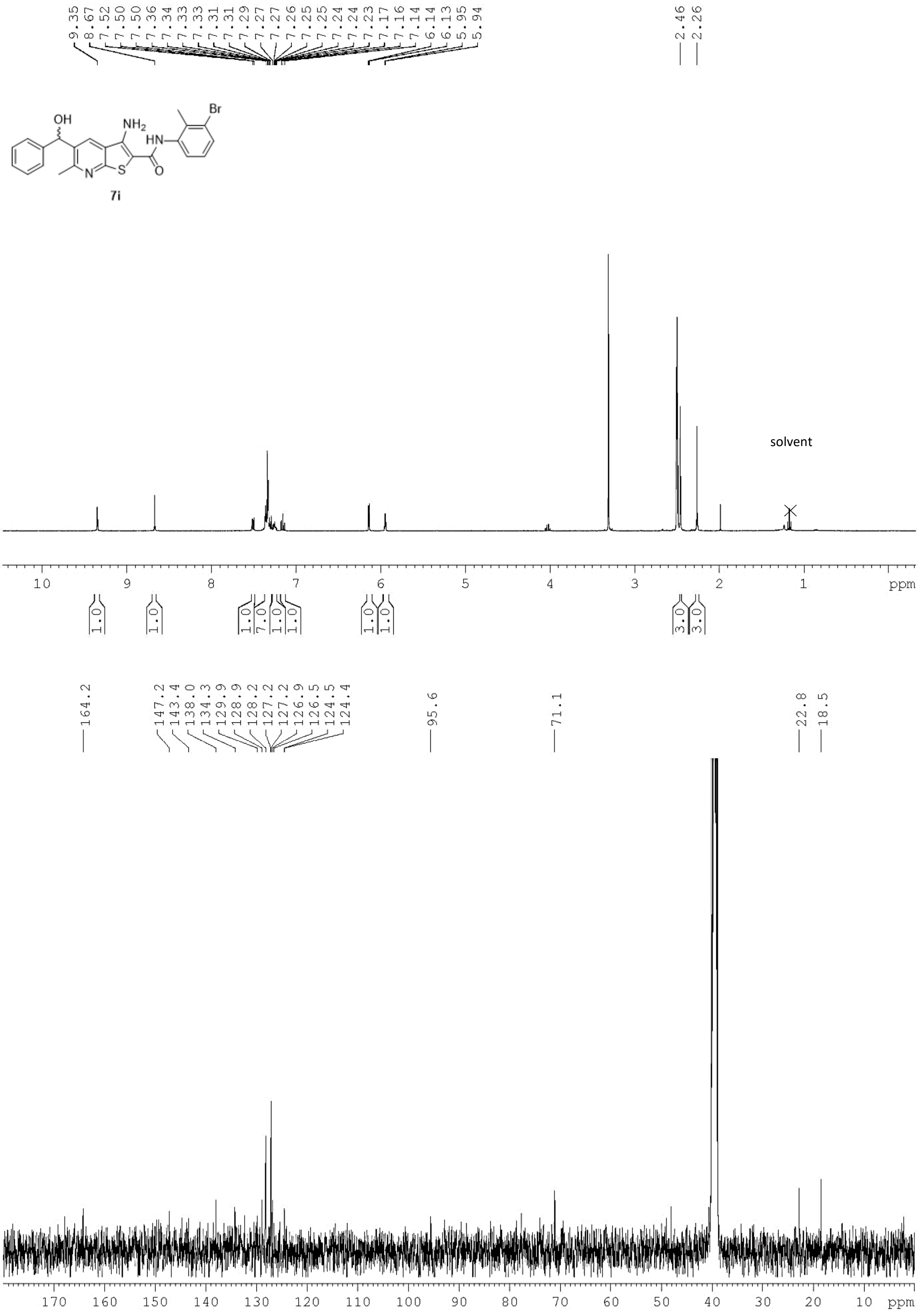

Figure S31: ^{1}H NMR and ^{13}C NMR spectra for **7i**.

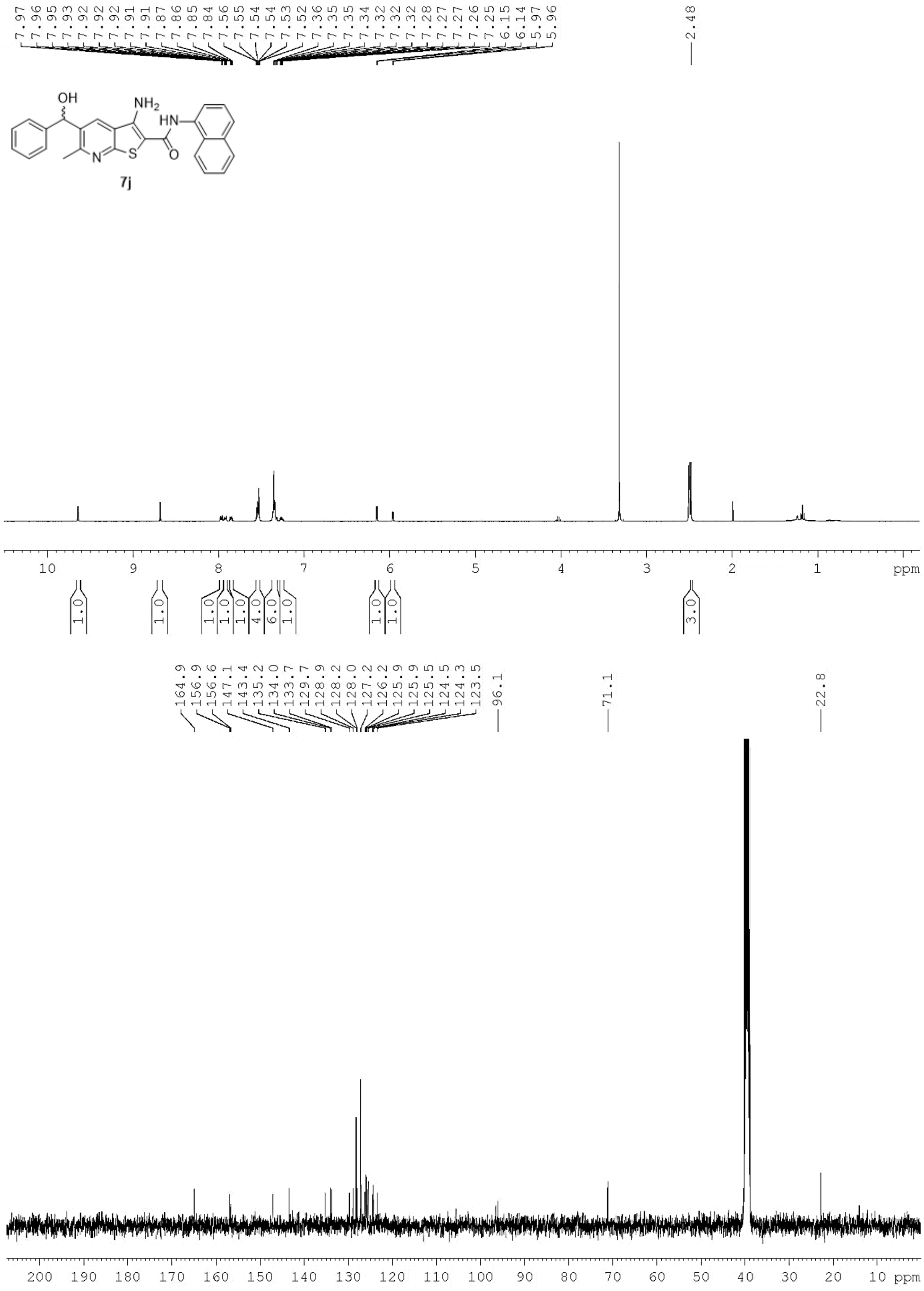

Figure S32: ^{1}H NMR and ^{13}C NMR spectra for **7j**.

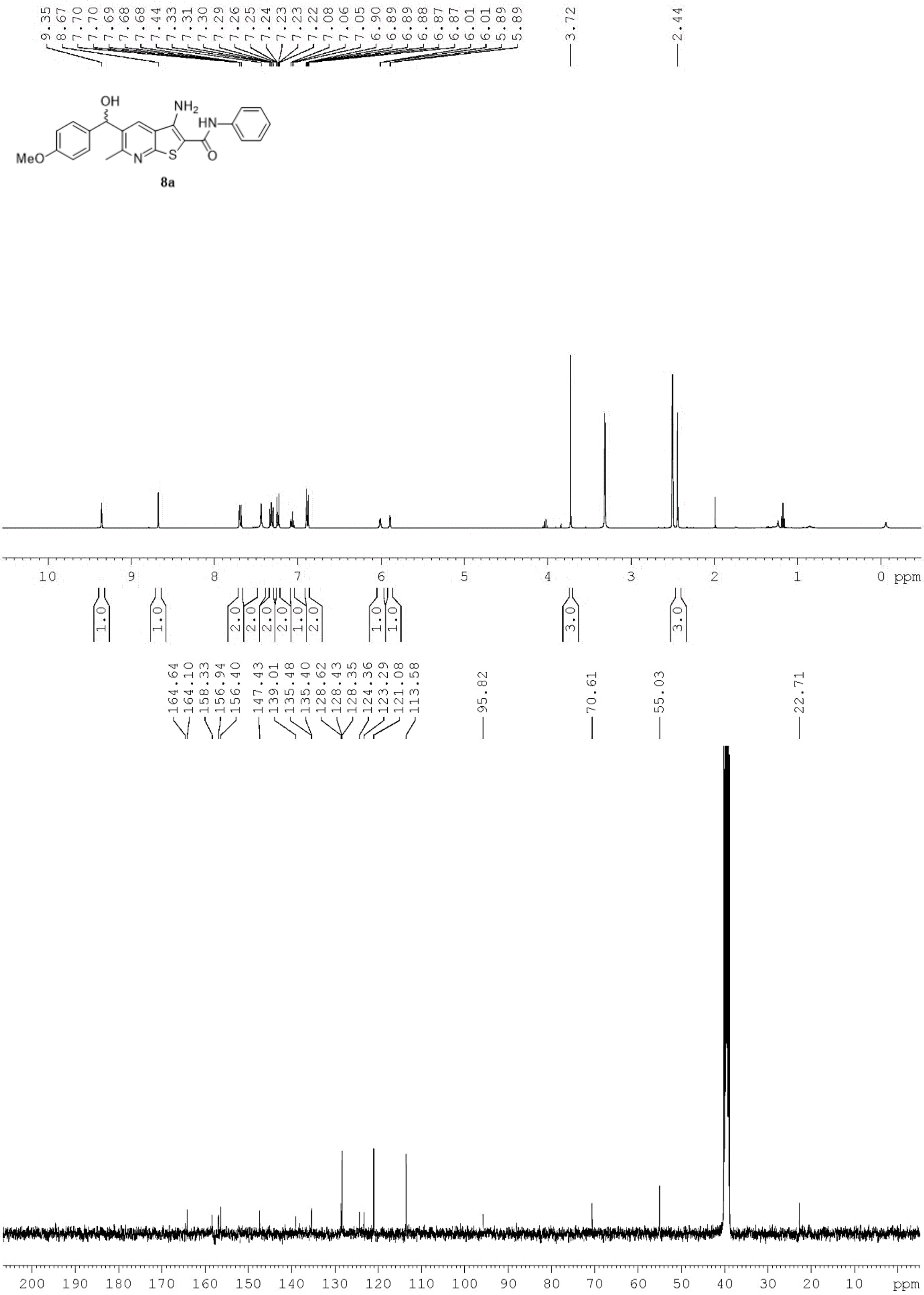

Figure S33: ^{1}H NMR and ^{13}C NMR spectra for **8a**.

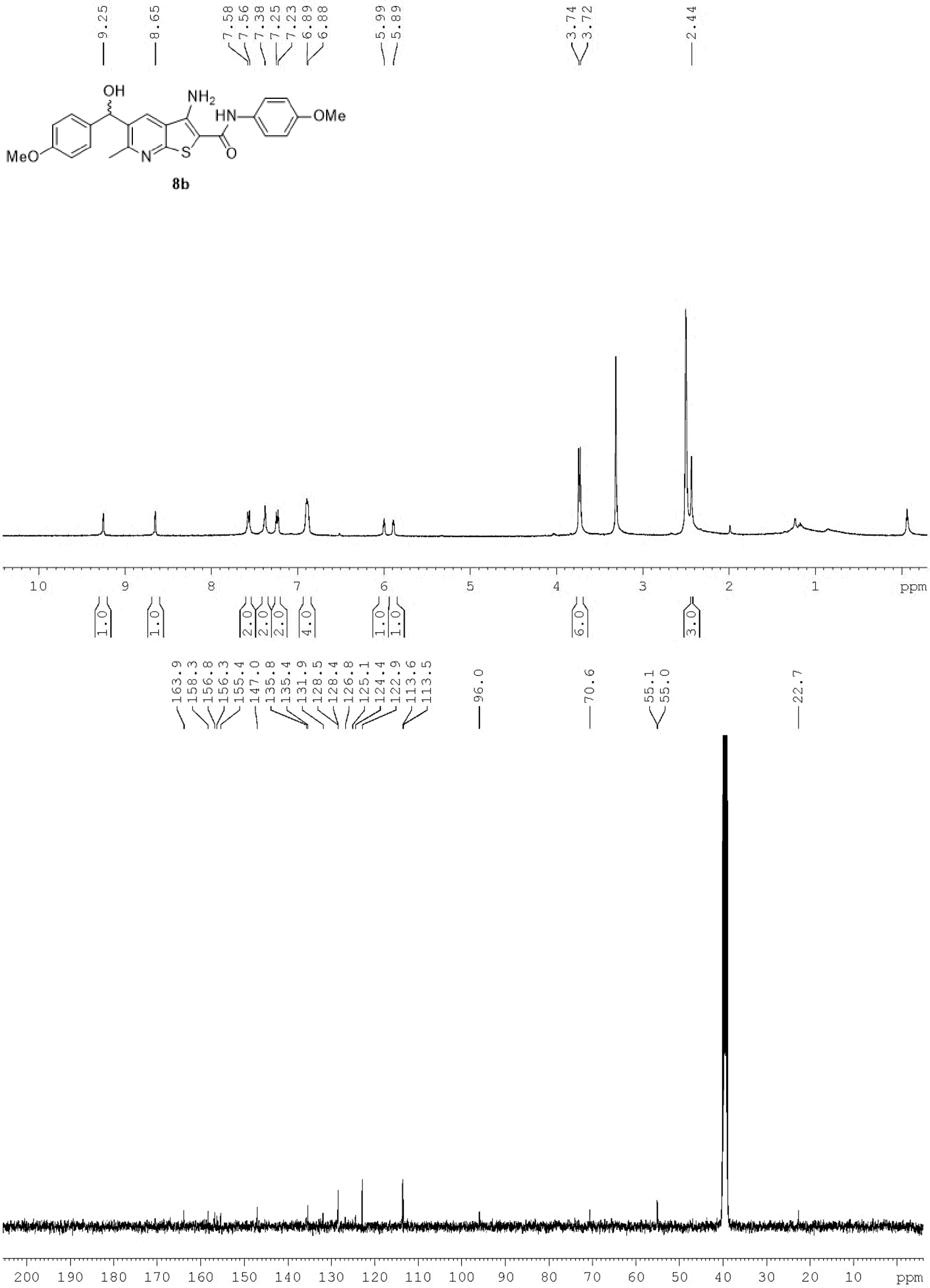

Figure S34: ^{1}H NMR and ^{13}C NMR spectra for **8b**.

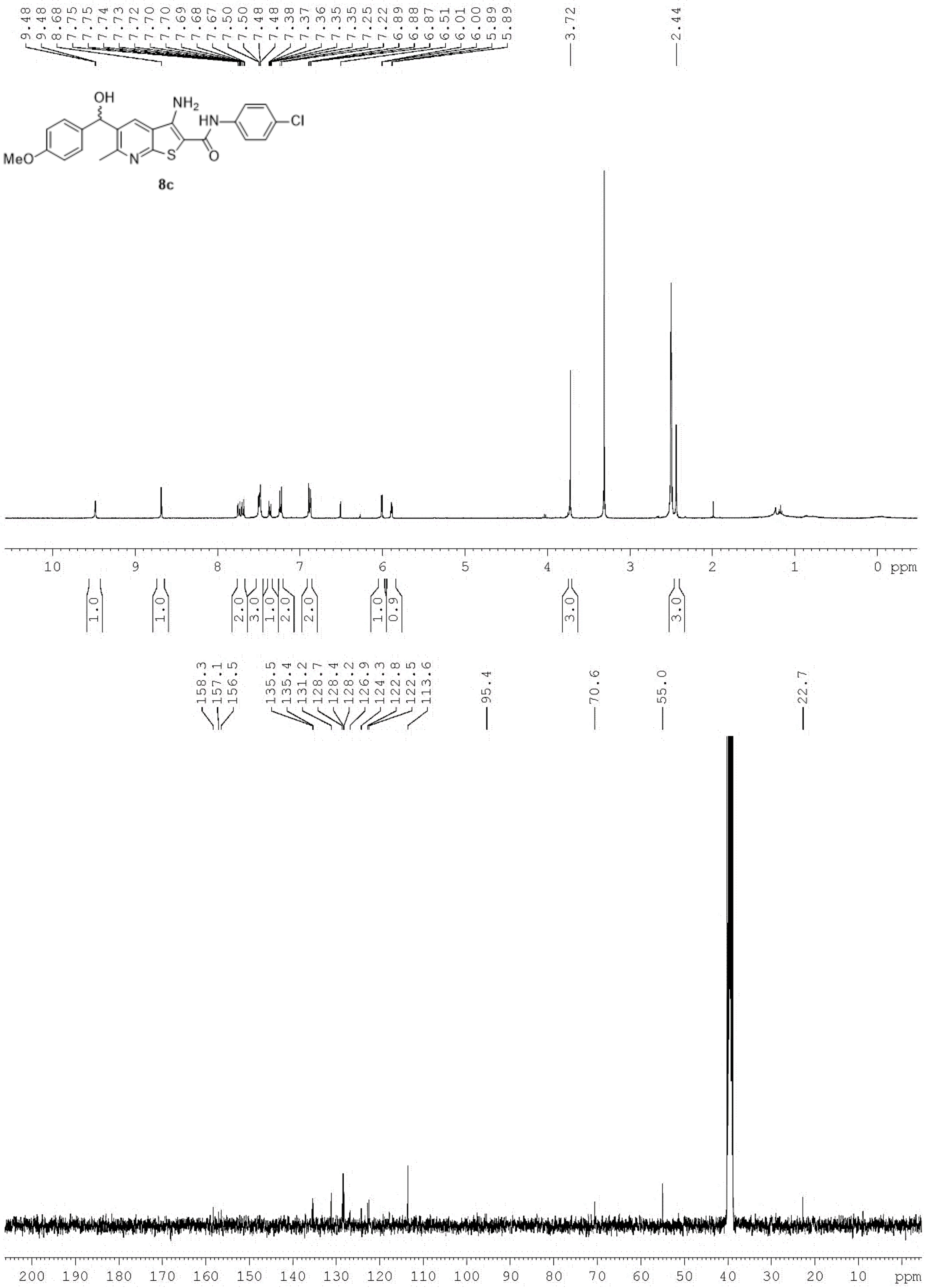

Figure S35: ^{1}H NMR and ^{13}C NMR spectra for **8c**.

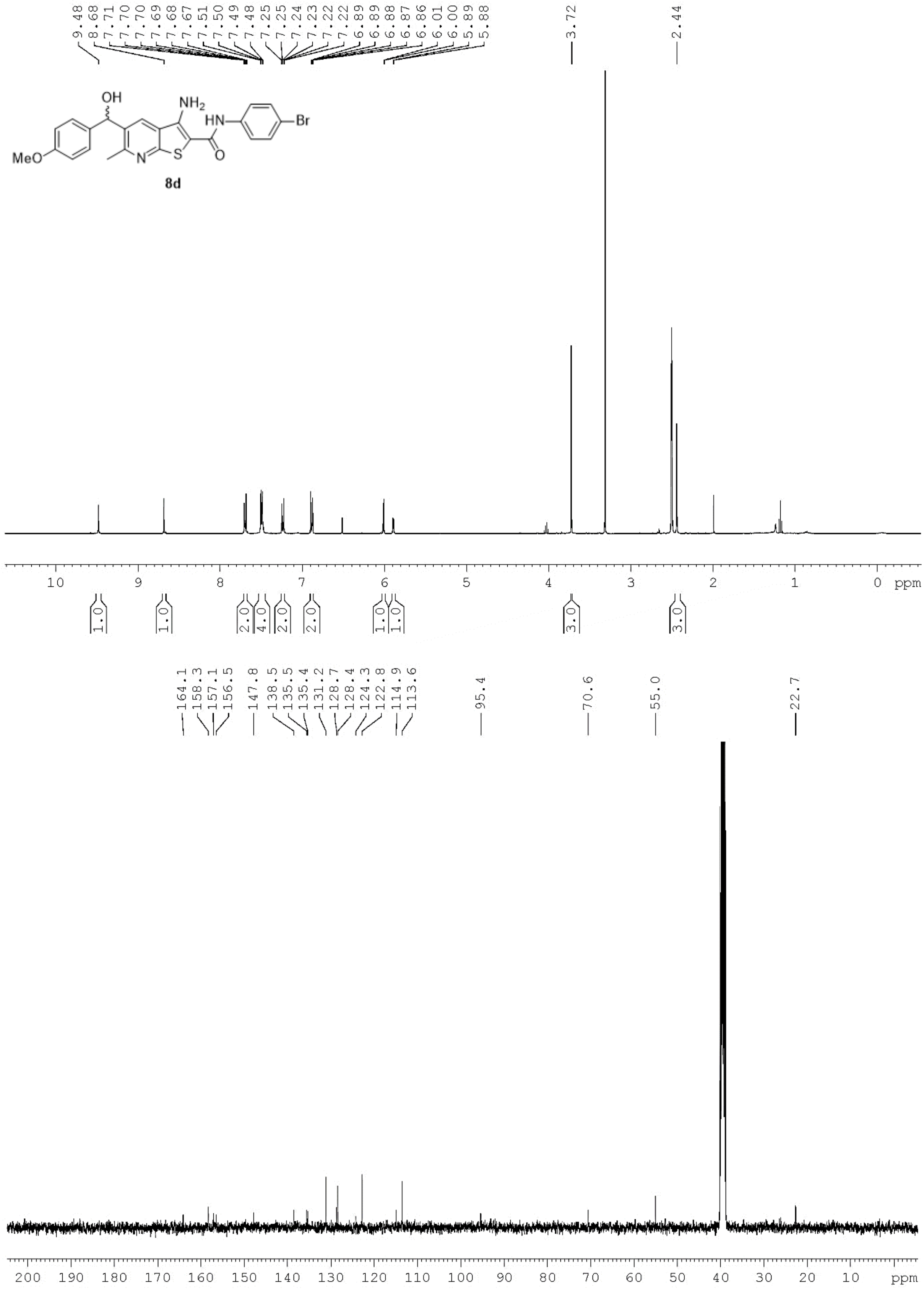

Figure S36: ^{1}H NMR and ^{13}C NMR spectra for **8d**.

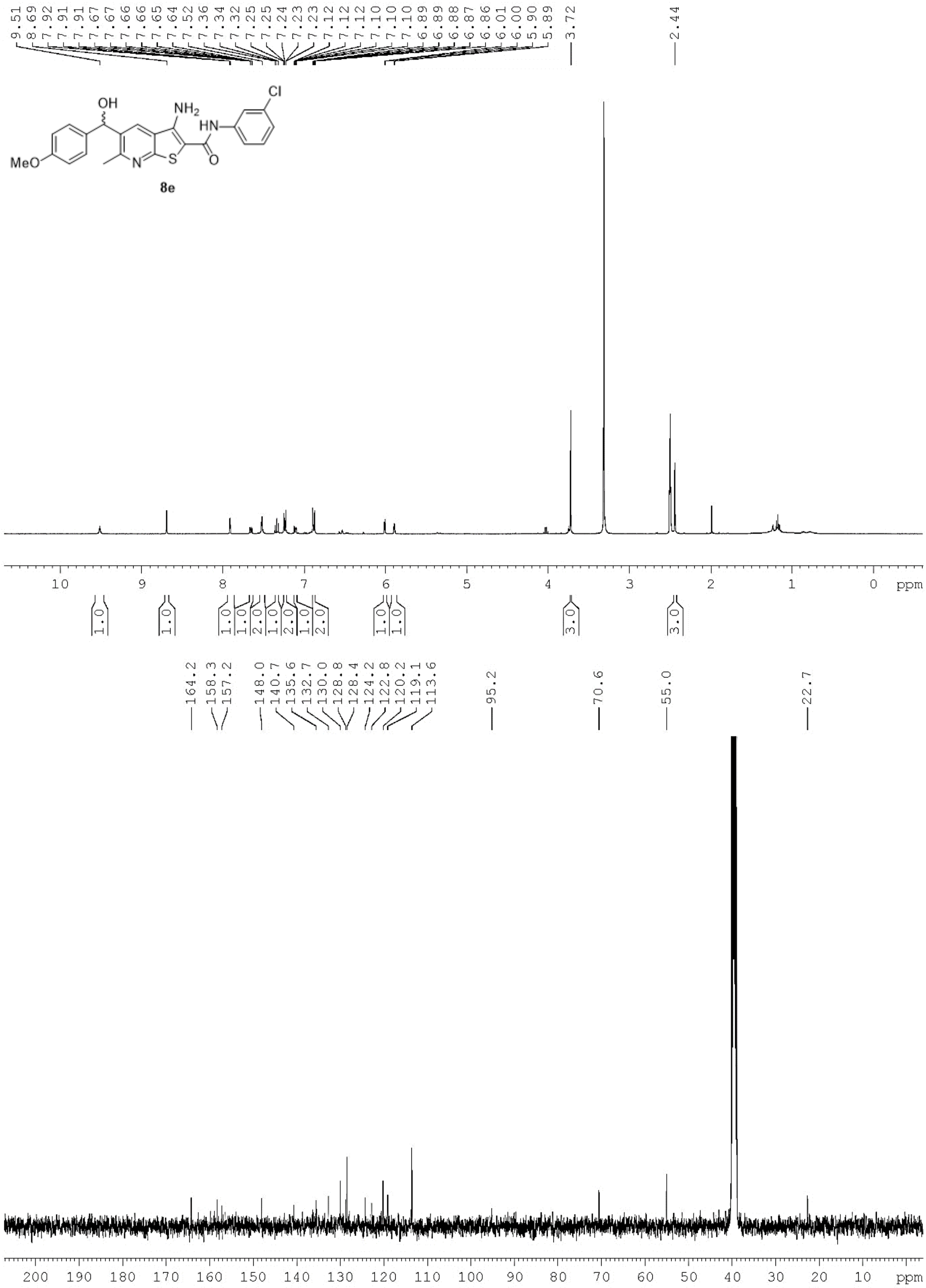

Figure S37: ^{1}H NMR and ^{13}C NMR spectra for **8e**.

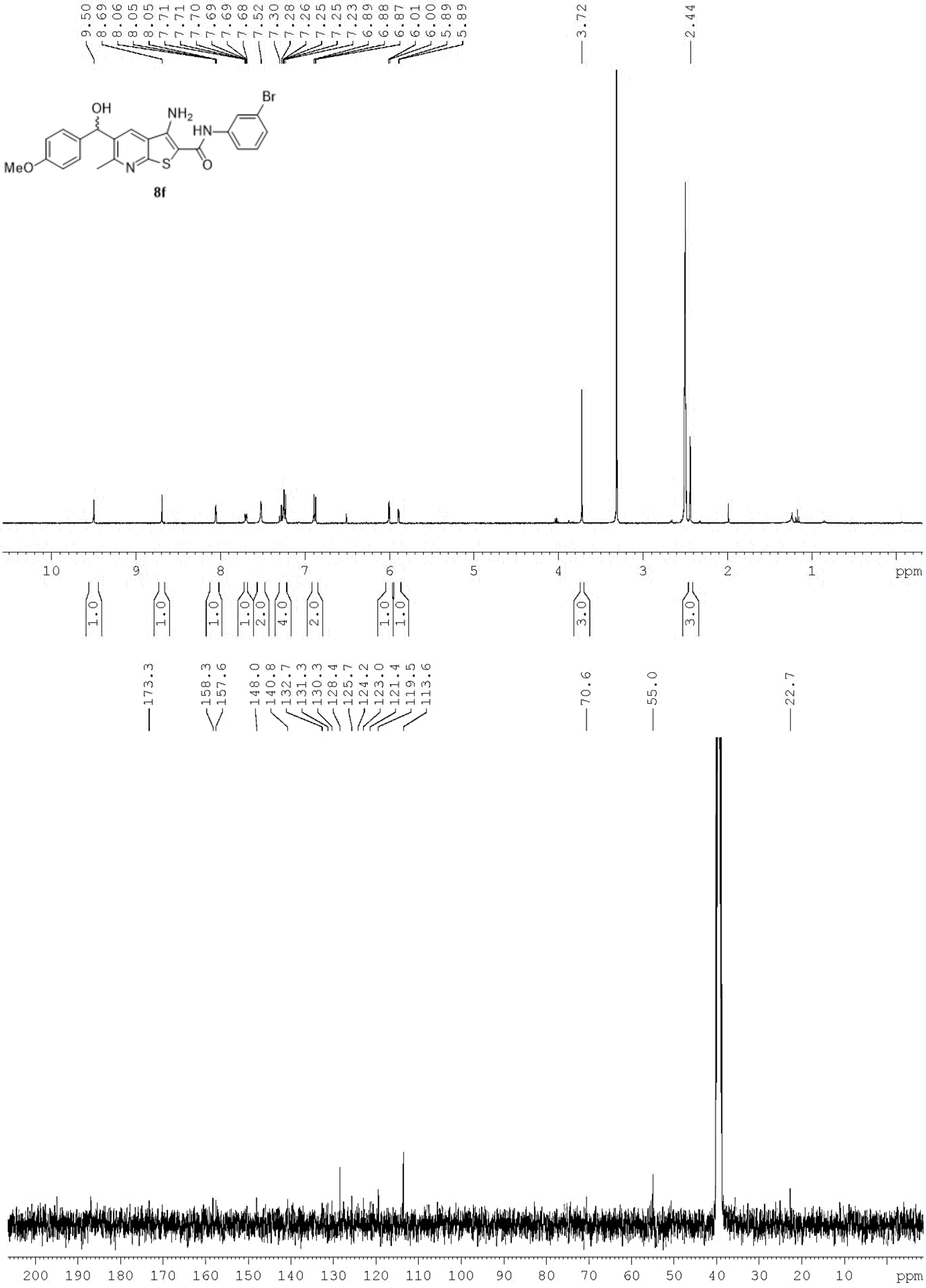

Figure S38: ^{1}H NMR and ^{13}C NMR spectra for **8f**.

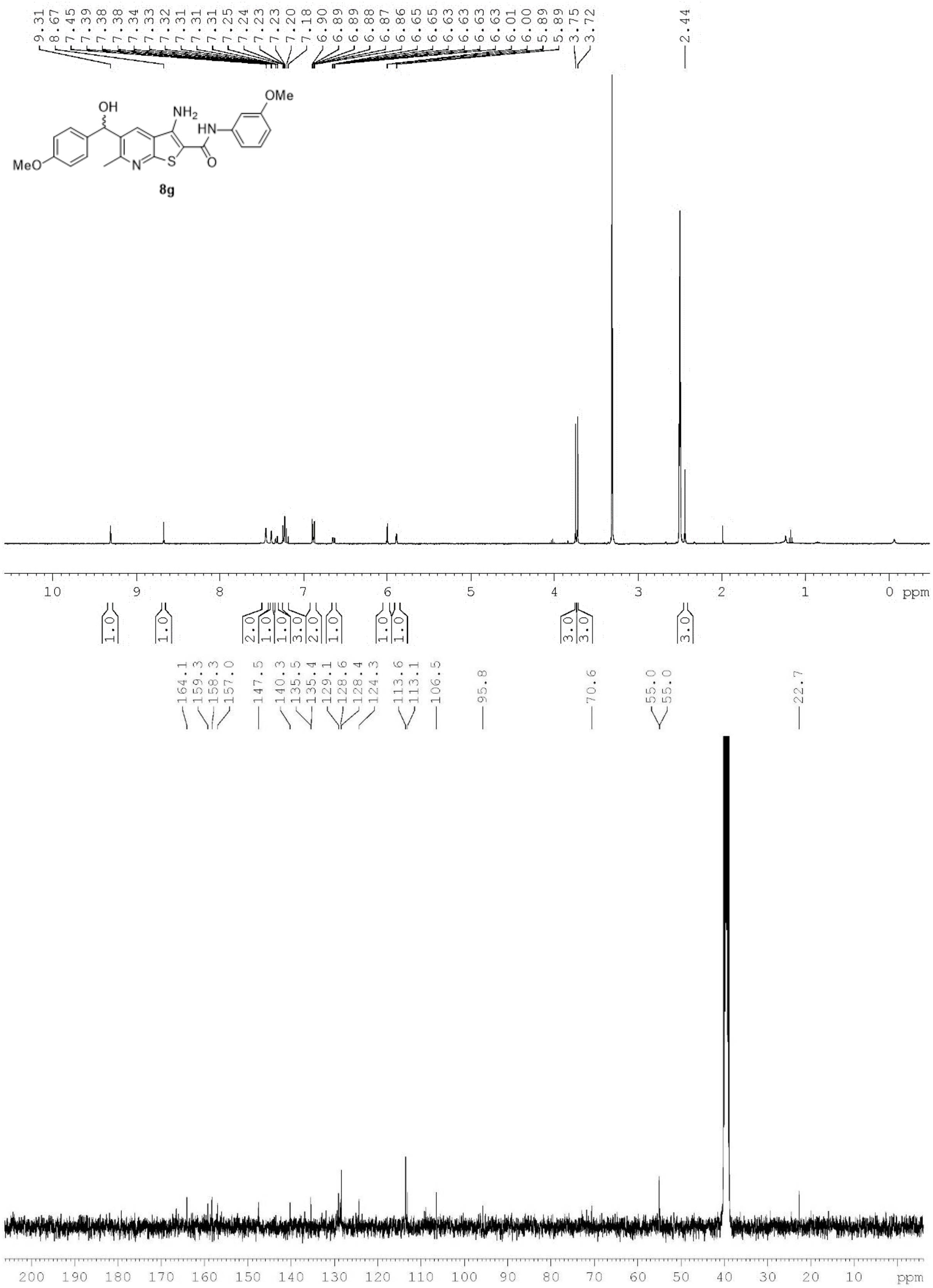

Figure S39: ^{1}H NMR and ^{13}C NMR spectra for **8g**.

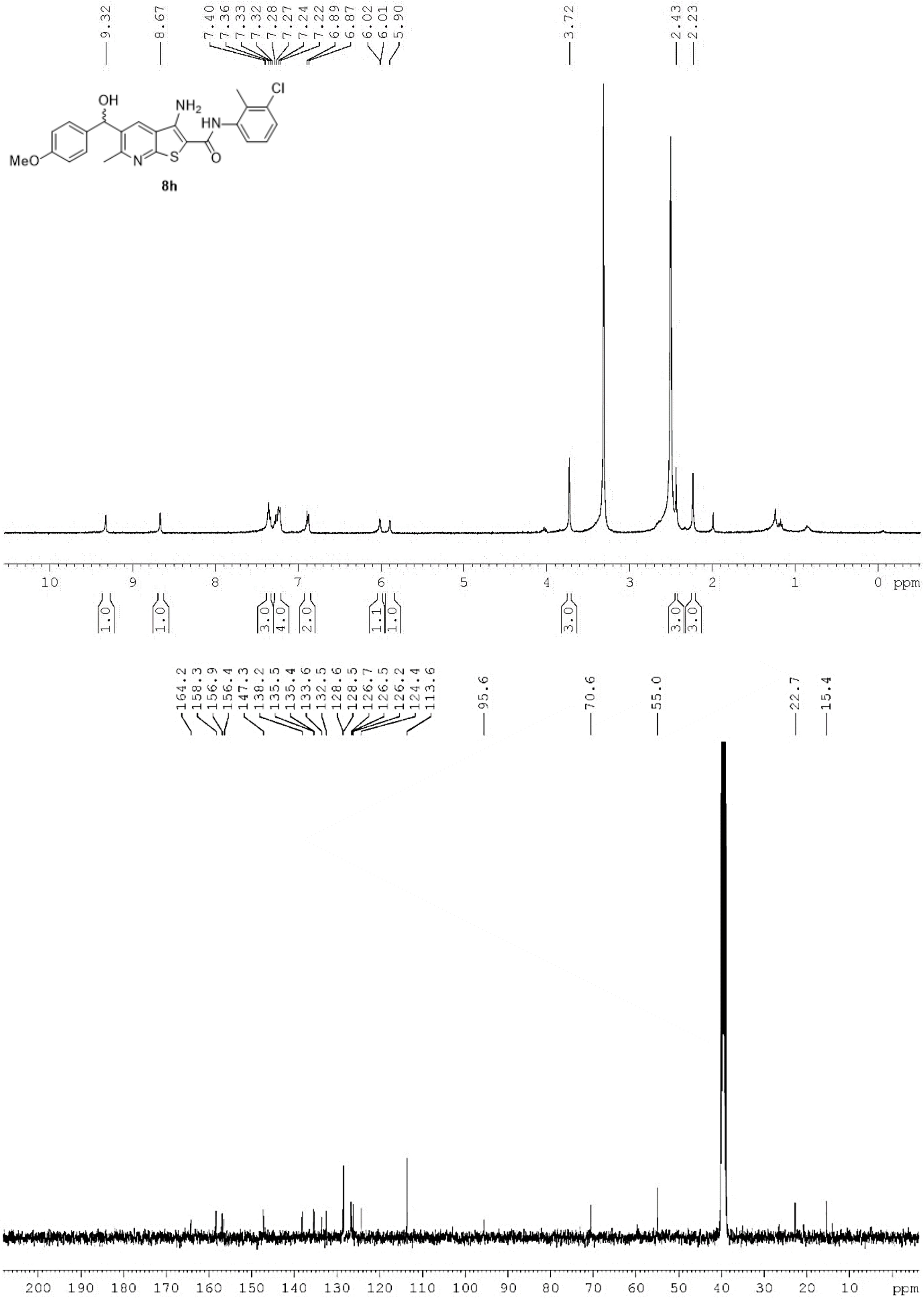

Figure S40: ^{1}H NMR and ^{13}C NMR spectra for **8h**.

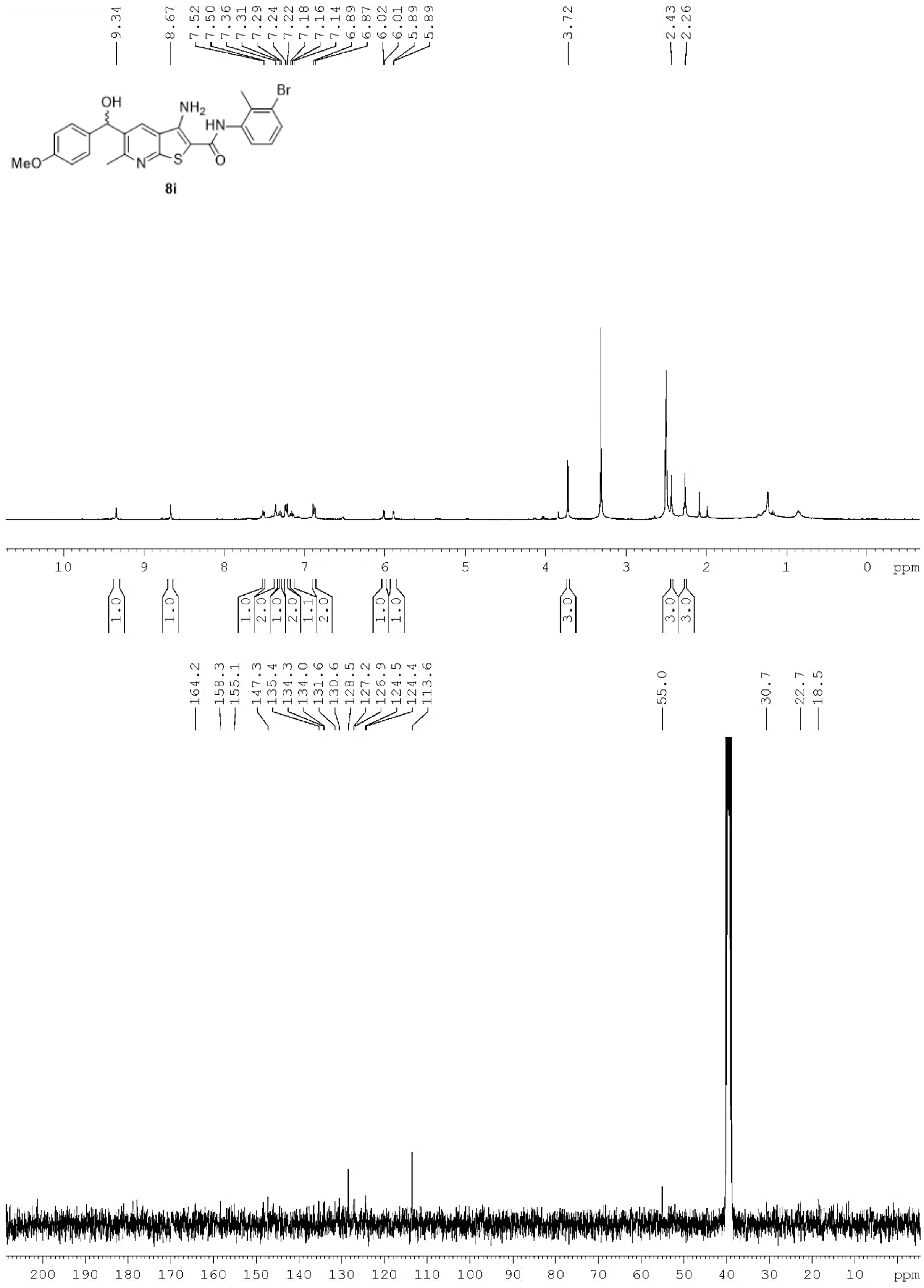

Figure S41: ^{1}H NMR and ^{13}C NMR spectra for **8i**.

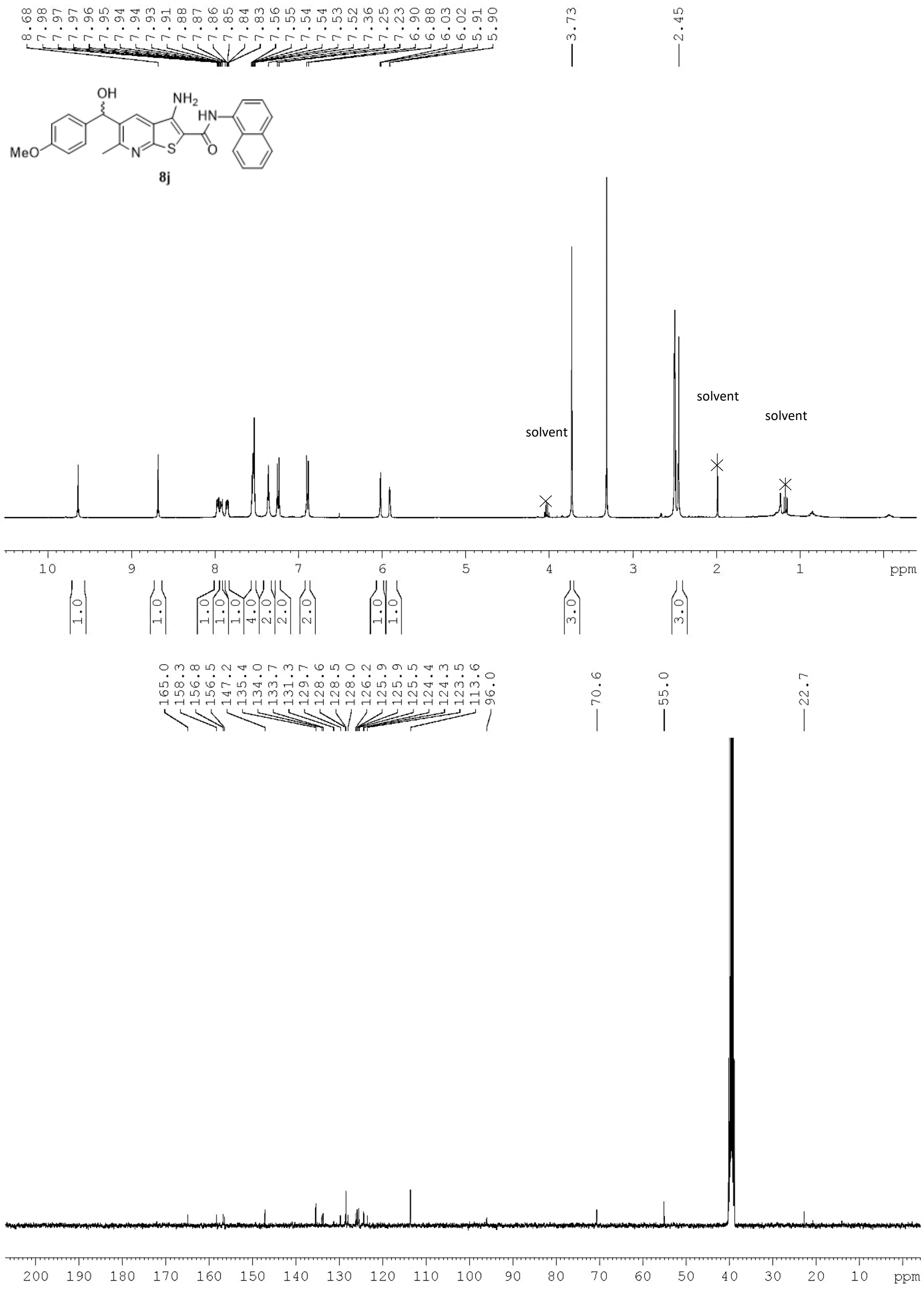

Figure S42: ^{1}H NMR and ^{13}C NMR spectra for **8j**.

Proposed mechanism for the formation of 12a-c and 12ai-ci:

It is postulated that this reaction mechanism between 2-cyanothioacetamide and **11a-c** involves conjugate addition, followed by E1cB elimination of dimethylamine to give the *E*-alkene intermediate followed by 6-exo-trig ring-closure by the thioamide group affording the desired products (**12a-c**, Figure S43). Isomers **12ai**, **12bi** and **12ci** are formed following ring closure onto the benzoyl ketone, which would have to result from the *Z*-alkene intermediate, formed less favourably due to increased steric congestion between the thioamide moiety and phenyl ring.

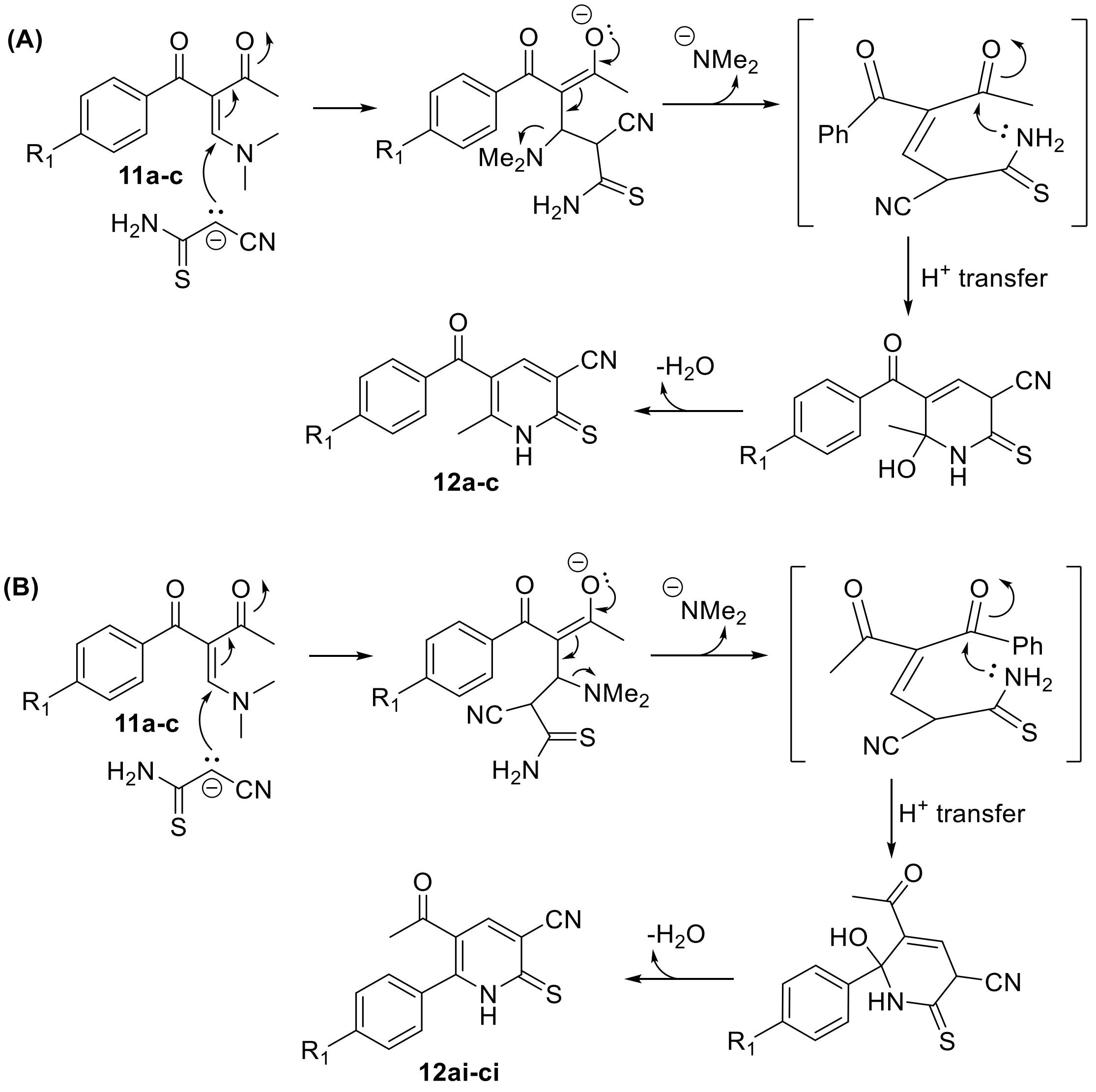

Figure S43: (A) Proposed mechanism for reaction between enamines **11a-c** and 2-cyanothioacetamide to afford carbonitriles **12a-c**. (B) Alternate mechanism demonstrating formation of minor isomers **12ai**, **12bi** and **12ci** via the *Z*-alkene intermediate.

Dose-Response Curves for compounds 5i, 7h, 7i and 8h:

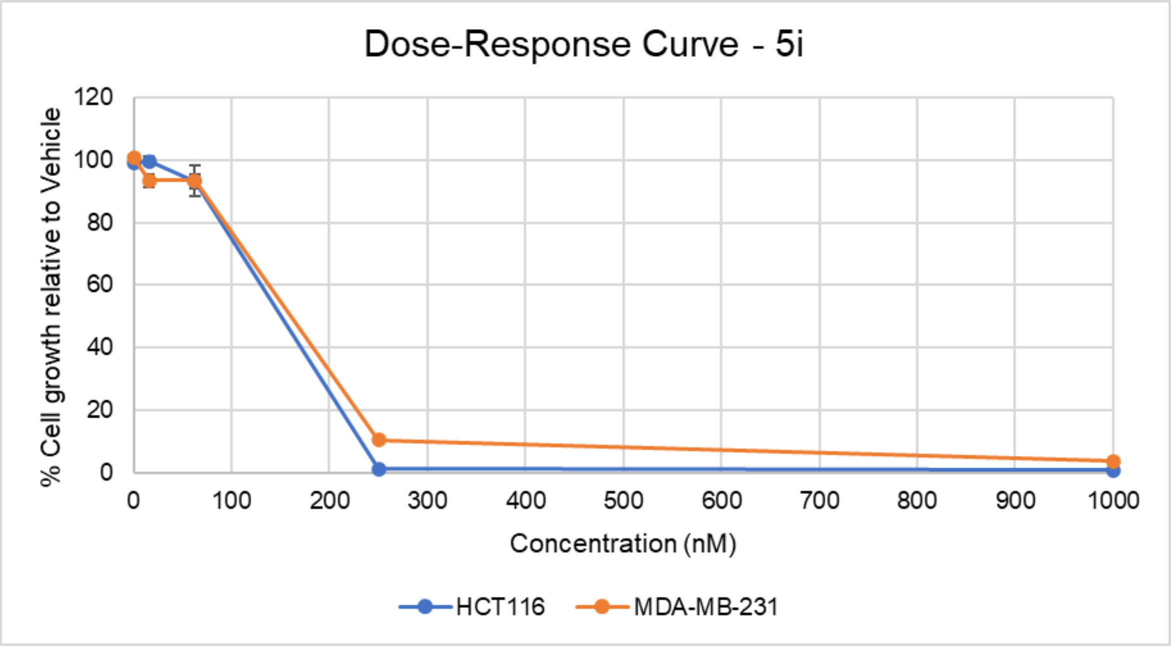

Figure S44: Dose-response curve for anti-proliferative activity induced by **5i** in HCT116 and MDA-MB-231 cell lines. Results are reported as mean ± s.e.m of two individual experiments.

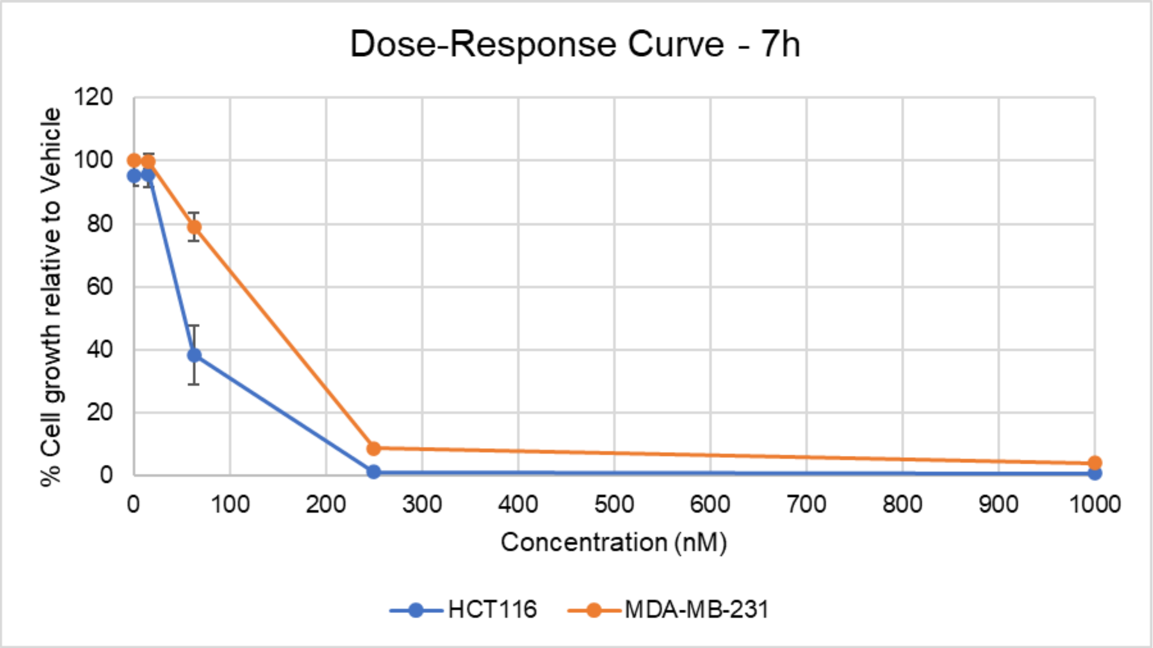

Figure S45: Dose-response curve for anti-proliferative activity induced by **7h** in HCT116 and MDA-MB-231 cell lines. Results are reported as mean ± s.e.m of two individual experiments.

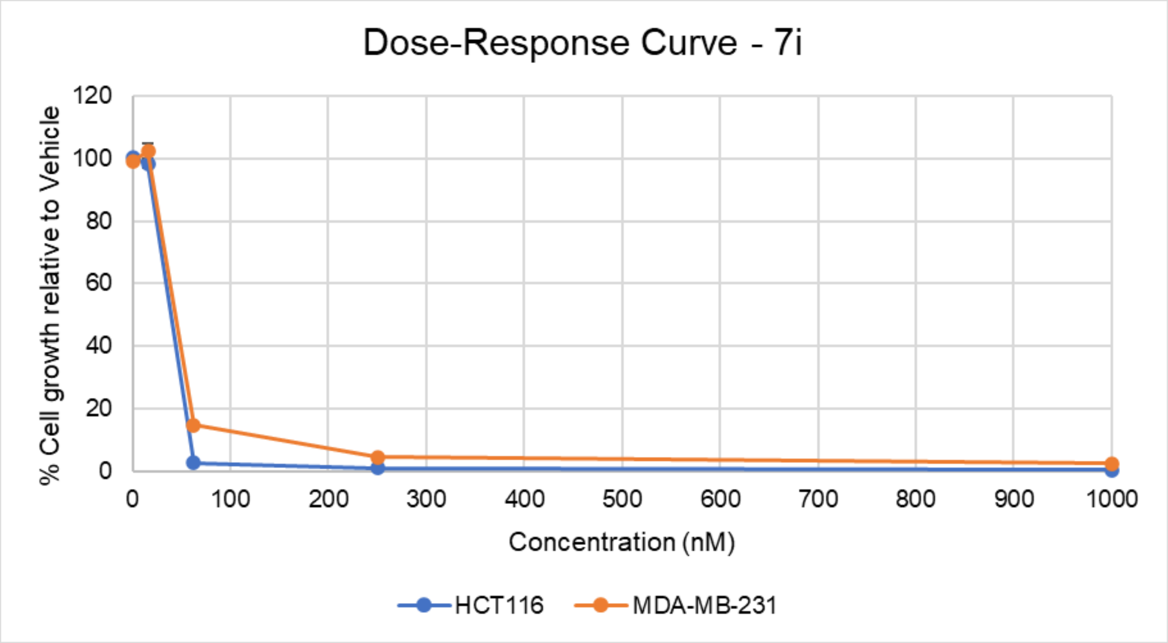

Figure S46: Dose-response curve for anti-proliferative activity induced by **7i** in HCT116 and MDA-MB-231 cell lines. Results are reported as mean ± s.e.m of two individual experiments.

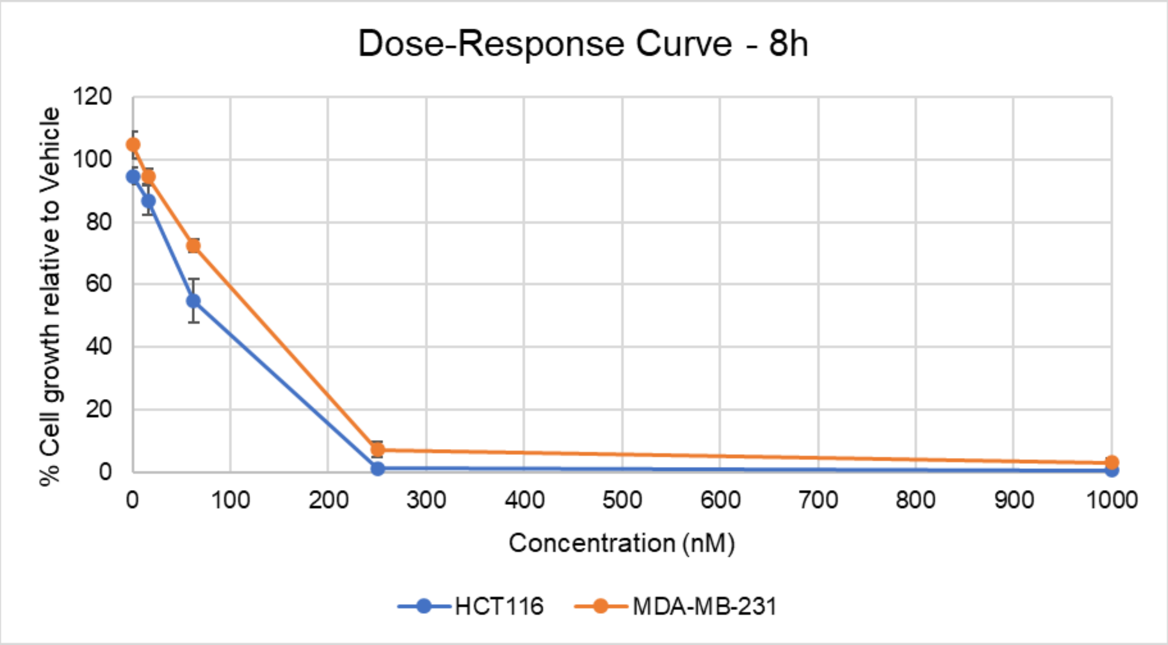

Figure S46: Dose-response curve for anti-proliferative activity induced by **8h** in HCT116 and MDA-MB-231 cell lines. Results are reported as mean ± s.e.m of two individual experiments.

References:

(1) Obydennov, D. L.; Goncharov, A. O.; Sosnovskikh, V. Ya. Preparative Synthesis of Ethyl 5-Acyl-4-Pyrone-2-Carboxylates and 6-Aryl-, 6-Alkyl-, and 5-Acylcomanic Acids on Their Basis. *Russ. Chem. Bull.* **2016**, *65* (9), 2233–2242. https://doi.org/10.1007/s11172-016-1574-x.

(2) Abu-Shanab, F. A.; Hessen, A. M.; Mousa, S. a. S. Dimethylformamide Dimethyl Acetal in Heterocyclic Synthesis: Synthesis of Polyfunctionally Substituted Pyridine Derivatives as Precursors to Bicycles and Polycycles. *J. Heterocycl. Chem.* **2007**, *44* (4), 787–791. https://doi.org/10.1002/jhet.5570440406.

(3) Baraldi, P. G.; Preti, D.; Tabrizi, M. A.; Fruttarolo, F.; Saponaro, G.; Baraldi, S.; Romagnoli, R.; Moorman, A. R.; Gessi, S.; Varani, K.; Borea, P. A. N6-[(Hetero)Aryl/(Cyclo)Alkyl-Carbamoyl-Methoxy-Phenyl]-(2-Chloro)-5′-N-Ethylcarboxamido-Adenosines: The First Example of Adenosine-Related Structures with Potent Agonist Activity at the Human A2B Adenosine Receptor. *Bioorg. Med. Chem.* **2007**, *15* (7), 2514–2527. https://doi.org/10.1016/j.bmc.2007.01.055.

(4) Pace, V.; Castoldi, L.; Holzer, W. Addition of Lithium Carbenoids to Isocyanates: A Direct Access to Synthetically Useful N-Substituted 2-Haloacetamides. *Chem. Commun.* **2013**, *49* (75), 8383–8385. https://doi.org/10.1039/C3CC44255A.

(5) Wang, G.-B.; Wang, L.-F.; Li, C.-Z.; Sun, J.; Zhou, G.-M.; Yang, D.-C. A Facile and Efficient Method for the Selective Deacylation of N-Arylacetamides and 2-Chloro-N-Arylacetamides Catalyzed by SOCl2. *Res. Chem. Intermed.* **2012**, *38* (1), 77–89. https://doi.org/10.1007/s11164-011-0327-6.

(6) Ghosh, K.; Sen, T. Naphthalene Appended 2,5-Diketopiperazine towards Fluorometric Response of Dihydrogenphosphate. *J. Incl. Phenom. Macrocycl. Chem.* **2010**, *68* (3), 447–452. https://doi.org/10.1007/s10847-010-9808-2.
